# Expanding the range of editable targets in the wheat genome using the variants of the Cas12a and Cas9 nucleases

**DOI:** 10.1111/pbi.13669

**Published:** 2021-07-28

**Authors:** Wei Wang, Bin Tian, Qianli Pan, Yueying Chen, Fei He, Guihua Bai, Alina Akhunova, Harold N. Trick, Eduard Akhunov

**Affiliations:** ^1^ Department of Plant Pathology Kansas State University Manhattan KS USA; ^2^ Hard Winter Wheat Genetics Research Unit USDA‐ARS Manhattan KS USA; ^3^ Integrated Genomics Facility Kansas State University Manhattan KS USA

**Keywords:** Wheat, genome editing, Cas12a, Cas9, protospacer‐adjacent motif, multiplex gene editing, grain size

## Abstract

The development of CRISPR‐based editors recognizing distinct protospacer‐adjacent motifs (PAMs), or having different spacer length/structure requirements broadens the range of possible genomic applications. We evaluated the natural and engineered variants of Cas12a (FnCas12a and LbCas12a) and Cas9 for their ability to induce mutations in endogenous genes controlling important agronomic traits in wheat. Unlike FnCas12a, LbCas12a‐induced mutations in the wheat genome, even though with a lower rate than that reported for SpCas9. The eight‐fold improvement in the gene editing efficiency was achieved for LbCas12a by using the guides flanked by ribozymes and driven by the RNA polymerase II promoter from switchgrass. The efficiency of multiplexed genome editing (MGE) using LbCas12a was mostly similar to that obtained using the simplex RNA guides and showed substantial increase after subjecting transgenic plants to high‐temperature treatment. We successfully applied LbCas12a‐MGE for generating heritable mutations in a gene controlling grain size and weight in wheat. We showed that the range of editable loci in the wheat genome could be further expanded by using the engineered variants of Cas12a (LbCas12a‐RVR) and Cas9 (Cas9‐NG and xCas9) that recognize the TATV and NG PAMs, respectively, with the Cas9‐NG showing higher editing efficiency on the targets with atypical PAMs compared to xCas9. In conclusion, our study reports a set of validated natural and engineered variants of Cas12a and Cas9 editors for targeting loci in the wheat genome not amenable to modification using the original SpCas9 nuclease.

## Introduction

Amongst considerations for designing genome editing experiments using an easily customizable CRISPR‐Cas system, one of the important factors is the availability of target sequences with specific protospacer‐adjacent motifs (PAMs). The Cas9‐based editor’s specificity is defined by the 20 nt‐long target sequence located next to G‐rich NGG PAM (Jinek *et al*., 2012), which limits availability of target sequences. The discovery of Cas12a nuclease (Zetsche *et al*., 2015) and engineered SpCas9 variants (Hu *et al*., 2018a; Nishimasu *et al*., 2018) allows to expand the number of editable target sites. The T‐rich PAMs of Cas12a (TTTV or TTV) and the atypical NG PAMs of xCas9 and Cas9‐NG allows for genome editing in the regions that might lack the G‐rich PAMs needed for SpCas9. The Cas12a nucleases isolated from different sources, MbCas12a from *Moraxella bovoculi 237*, FnCas12a from *Francisella novicida* U112, AsCas12a from *Acidaminococcus* sp. *BV3L6* and LbCas12a from *Lachnospiraceae bacterium ND2006* were shown to induce detectable mutations in human and mammalian cells (Toth *et al*., 2018; Tu *et al*., 2017; Zetsche *et al*., 2015). In most cases, LbCas12a demonstrated the highest gene editing efficiency (Toth *et al*., 2018). All four Cas12a orthologues recognize the TTTV PAM (Toth *et al*., 2018), except for FnCpf1, which also recognizes the TTV PAM (Tu *et al*., 2017). The development of engineered AsCas12a (carrying mutations S542R/K607R or S542R/K548V/N552R) and LbCas12a (carrying mutations G532R/K595R or G532R/K538V/Y542R) with altered PAMs (TYCV or TATV) further broadened the utility of the Cas12a system for genome engineering applications (Gao *et al*., 2017).

The application of CRISPR‐Cas12a system has been widely studied in different plant species including *Arabidopsis*, rice, soybean and tobacco (Begemann *et al*., 2017; Endo *et al*., 2016; Hu *et al*., 2017; Kim *et al*., 2017; Tang *et al*., 2017; Xu *et al*., 2017; Yin *et al*., 2017). In rice, the efficiency of genome editing with LbCas12a was comparable to that observed for Cas9, whilst mutations induced by AsCas12a were hardly detectable (Li *et al*., 2018b; Tang *et al*., 2017). In addition, the LbCas12a‐mediated sequence replacement was also achieved in rice (Li *et al*., 2018a, 2019b, 2020). FnCas12a, which has shorter PAM (TTV), was proved to induce robust DNA cleavage in multiple species, including such model species as rice and tobacco (Begemann *et al*., 2017; Endo *et al*., 2016; Wang *et al*., 2017, 2018a; Zhong *et al*., 2018). Recently, the Cas12a orthologues ErCas12a, Lb5Cas12a, BsCas12a, Mb2Cas12a, TsCas12a and MbCas12a were shown to be highly effective for gene editing in rice (Zhang *et al*., 2021). In wheat, until now, only two target sites in the exogenous gene GUS (β‐glucuronidase) were edited using LbCas12a (Liu *et al*., 2020), with one of the targets showing the genome editing efficiency lower than that of Cas9.

The efficiency of multiplex genome editing (MGE) is another important consideration when choosing the CRISPR‐Cas system. To edit multiple targets in a genome using the CRISPR‐Cas9 system, multiple guide RNAs (gRNAs) should either be expressed each from its own independent promoter or be expressed as a long tandem gRNA array with individual units separated by spacers, which should undergo endogenous or exogenous RNA processing to produce functional RNA guides (Wang *et al*., 2016; Xing *et al*., 2014). The ability of Cas12a to process a precursor crRNA array (Fonfara *et al*., 2016; Zetsche *et al*., 2017) permits using it without need to separate each guide by a spacer sequence. Additionally, the short length of crRNAs makes the CRISPR‐Cas12a system an ideal MGE tool, which was successfully applied to editing the genome of different species, including rice (Wang *et al*., 2017; Zhang *et al*., 2021).

To expand the range of editable targets, one of the most broadly used nucleases, SpCas9, was engineered to recognize the NG PAM instead of NGG. The two engineered variants, referred to as xCas9 and Cas9‐NG, were initially tested in human/mammalian cells (Hu *et al*., 2018a; Nishimasu *et al*., 2018), and later in other species, including some crops (Li *et al*., 2019a; Ren *et al*., 2019; Zeng *et al*., 2019; Zhong *et al*., 2019). The xCas9 nuclease was substantially less effective than SpCas9 for editing the exogenous GUS gene in wheat (Liu *et al*., 2020), and less effective than Cas9‐NG for editing the endogenous targets in rice (Zeng *et al*., 2019; Zhong *et al*., 2019). However, the application of both xCas9 and Cas9‐NG for engineering the endogenous gene targets in wheat was not reported.

In this study, we investigated the ability of the natural and engineered variants of Cas12a and Cas9 to target sites with the canonical and non‐canonical PAMs in the wheat genome using the simplex and multiplex gene editing constructs. We showed that high‐temperature treatment of transgenic wheat substantially improves the efficiency of MGE with the Cas12a system. Using Cas12a, we have produced a stable mutant with heritable mutations in a gene affecting grain size and weight in wheat. We sought to improve the performance of the Cas12a system in wheat by evaluating constructs with human and plant codon‐optimized variants of Cas12a expressing guide RNAs under the control of the PvUbip promoter from switchgrass, and assessing the effect of ribozyme‐based guide processing on the efficiency of genome editing. We have demonstrated the ability of the engineered Cas12a and Cas9 nucleases with the altered PAMs to induce mutations at targets inaccessible to Cas9, expanding further the range of editable loci in the wheat genome.

## Results

### Comparison of LbCas12a and FnCas12a genome editing efficiency in wheat

**Figure 1:**
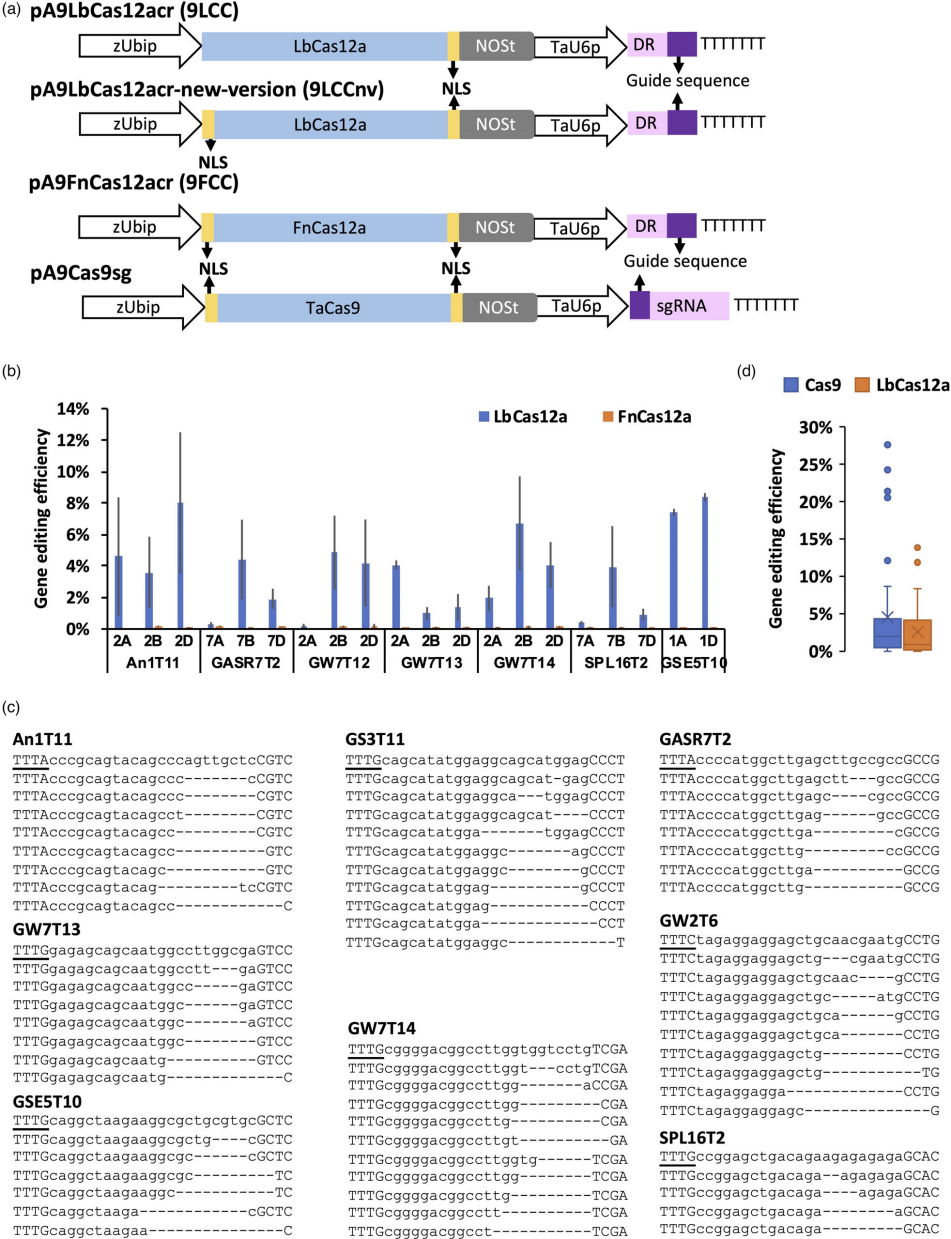
LbCas12a but not FnCas12a induces mutations in the wheat genome. (a) A schematic illustration of plasmids pA9LbCas12aCr (9LCC for short), pA9LbCas12aCr‐new‐version (9LCCnv for short), pA9FnCas12aCr (9FCC for short) and pA9Cas9sg. Both the maize *Ubiquitin* promoter and wheat U6 promoter are shown with open arrows and marked as zUbip and TaU6p, respectively. The coding sequences of *LbCas12a*, *FnCas12a* and wheat codon‐optimized *Cas9* (*TaCas9*) are shown as blue rectangles. The NLS peptide sequences that flank the *Cas12a* coding sequences are shown as yellow rectangles. The NOS poly A terminator is shown as grey rectangle and marked as NOSt. The direct repeat (DR) and guide sequence of crRNA are shown as pink and purple rectangle, respectively. The sequence of seven ‘T’ bases is terminator for TaU6p. (b) The comparison of the normalized gene editing efficiency between LbCas12a (9LCC/9LCCnv) and FnCas12a (9FCC). As the construct 9LCC and 9LCCnv showed no significant difference (*P* > 0.05 by student TTEST) on gene editing efficiency at target GSE5T10 and GS3T11 (Table S2), data of targets gotten assessed by either 9LCC or 9LCCnv were all used for the comparison between LbCas12a and FnCas12a. The 9LCC data of GSE5T10 from Table S2 was selected to prepare this plot because it showed the lower levels of variability in gene editing efficiency compared to 9LCCnv. The bar plots showed the data as mean ± standard error. Each target had three biological repeats. In cultivar Bobwhite, the B genome homoeolog of *TaGSE5* is highly divergent from the A and D genome copies and could not be targeted by the designed gRNA. (c) The representative NGS reads generated for regions targeted by CRISPR‐LbCas12a. The sequences of the wild‐type alleles are shown on the top. The PAM and target sequences are shown as underlined and lower‐case letters, respectively. (d) The comparison of average gene editing efficiency for Cas9 (21 targets) and LbCas12a (12 targets) calculated for genes *TaGS3*, *TaGW7*, *TaPDS* and *TaGSE5* (Table S2 and Table [Link], [Link]). The duplicated target sites in the A, B and D genomes were treated as independent targets. The gene editing efficiency of each target was normalized by protoplast transformation efficiency. The means of two or three biological replicates for each target were used to make the box and whisker plot.

Because AsCas12a showed lower gene editing (GE) efficiency in both rice and tobacco compared to LbCas12a (Bernabe‐Orts *et al*., 2019; Tang *et al*., 2017), in this study, we focussed on evaluating the GE efficiency of LbCas12a and FnCas12a, which recognize the TTTV and TTV PAMs, respectively (Figure 1a). In total, 17 RNA guides were designed for LbCas12a to target eight genes including *TaAn‐1*, *TaGASR7*, *TaGS3*, *TaGSE5*, *TaGW2*, *TaGW7*, *TaPDS* and *TaSPL16* (Table S1). These genes, except *TaPDS*, have orthologues in rice and other crops that control the grain number, grain size or grain weight traits. The *TaPDS* encodes phytoene desaturase, an important enzyme in the carotenoid biosynthesis pathway. Two LbCas12a constructs, 9LCC, which has only the C‐terminal nuclear localization signal (NLS), and 9LCCnv, which has both the N‐ and C‐terminal NLSs on Cas12a (Figure 1a and Figure S1), were used for assessing the GE efficiency. The constructs 9LCC and 9LCCnv showed no significant difference (Student’s *t* test *P* > 0.05) in GE efficiency for targets GSE5T10 and GS3T11 (Table S2). The efficiency of GE for 11 gene targets assessed separately for each homoeologous genome in the wheat protoplast, after correcting for transformation efficiency, was higher than 1% in at least one genome (Figure 1b; Table S2), with the highest mean editing efficiency reaching 23.8%. Most of the mutations induced by LbCas12a were deletions longer than 3 bp located at the 3′ end of protospacers distal from PAMs (Figure 1c).

Due to overlap of PAM sequences, the guides designed for LbCas12a should target the same genes when used with FnCas12a. However, using 16 guide sequences designed for LbCas12a and two guides specifically designed for the targets carrying the TTV PAM, we could not identify FnCas12a‐induced mutations at the target sites in the wheat protoplasts (Figure 1b; Table S3).

We compared the proportion of targets that could be edited by LbCas12a and Cas9 with the efficiency of ≥1%. For this purpose, we designed the CRISPR‐Cas9 gRNAs to target the coding sequences of the *TaGS3*, *TaGSE5* and *TaPDS* genes, subcloned them into the CRISPR‐Cas9 construct pA9Cas9sg (Figure 1a) and evaluated their performance in the protoplasts isolated form cv. Bobwhite. In addition, we used the previously reported GE efficiency data for the CRISPR‐Cas9 RNA guides targeting the *TaGW7* gene (Wang *et al*., 2019) to compare with the performance of the LbCas12a RNA guides targeting the same gene. In total, 21 Cas9 guides targeting 58 loci and 12 LbCas12a guides targeting 34 loci were compared. The results show that 37 of 58 (64%) Cas9‐targeted loci and 14 of 34 (41%) LbCas12a‐targeted loci had mutation rates higher than 1% (Table S2 and S4). The highest GE efficiencies observed for the LbCas12a targets within genes *TaGW7*, *TaGS3*, *TaGSE5* and *TaPDS* were 6.7%, 13.8%, 8.4% and 3.74%, respectively, after 48 h of the protoplast transformation. The highest GE efficiencies for the Cas9 targets within the *TaGW7*, *TaGS3*, *TaGSE5* and *TaPDS* genes were 28.4%, 7.7%, 12.1% and 24.2%, respectively (Table S2 and S4). Overall, Cas9 has higher chance to find better performing guides compared to LbCas12a (Figure 1d).

### Effect of high‐temperature treatment on CRISPR‐Cas12a‐based multiplex gene editing

**Figure 2:**
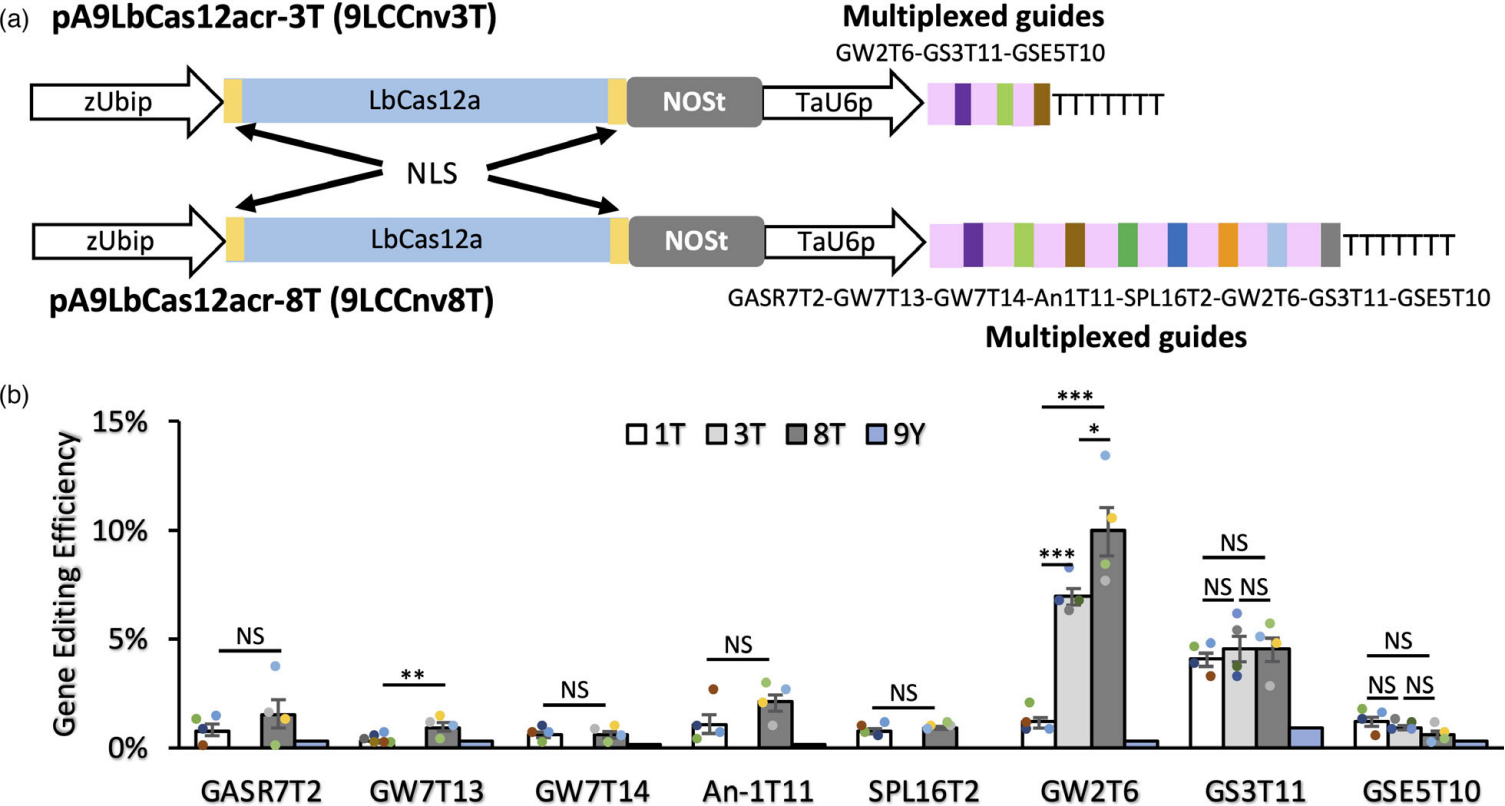
The gene editing efficiency of CRISPR‐LbCas12a constructs with single and multiple crRNA units. (a) Schematic illustration of the CRISPR‐LbCas12a MGE constructs. The MGE constructs targeting three and eight targets are referred to as pA9LbCas12acr‐3T (or 9LCCnv3T) and pA9LbCas12acr‐8T (or 9LCCnv8T), respectively. The order of guide sequences for different targets are shown next to the construct names. (b) The bar plots of gene editing efficiency for the CRISPR‐LbCas12a constructs targeting one (1T), three (3T) and eight (8T) targets. Data are shown as mean ± standard error. The data generated from the protoplasts transformed with construct pA9eYFP that carries the *YFP* gene was used as negative control (marked as 9Y). Because targets located in the A, B and D genomes showed similar LbCas12a‐induced mutation rates, data generated for all three genomes was pooled together for calculating the gene editing efficiency. The analyses of each target site were based on four biological replicates, the result of each replicate is shown as coloured dots on the bar plots. Student’s *t* test was used to assess the significance of differences in gene editing efficiency between guides from the constructs with one, three or eight crRNAs. *P ≤ 0.05; **P ≤ 0.01; ***P ≤ 0.001; ^NS^ *P* > 0.05.

The MGE efficiency of the CRISPR‐Cas12a system was investigated by transforming the wheat protoplasts using the constructs with the tandem arrays of three, four or eight crRNA units (Figure 2a and Figure S2). The LbCas12a‐induced mutations were detected for all multiplexed targets (Table S5). The editing efficiency of the multiplexed guides from the LbCas12a MGE constructs was largely comparable to that of the LbCas12a constructs carrying only one guide (Figure 2b and Figure S2, *t*‐test *P* > 0.05). Compared to constructs expressing single guides, the GE efficiencies of GS3T11 in the construct expressing 4 crRNAs (9LCCnv4T), GW7T13 in the construct expressing 8 crRNAs (9LCCnv8T), and GW2T6 in all tested MGE constructs were significantly increased (Figure 2b and Figure S2, *t*‐test *P* < 0.05). For unknown reasons, we observed increase in the GE efficiency of GW2T6 with increase in the number of crRNA units in the LbCas12a MGE constructs (Figure 2b and Figure S2). No similar increase in editing efficiency was observed for other guides.

**Figure 3:**
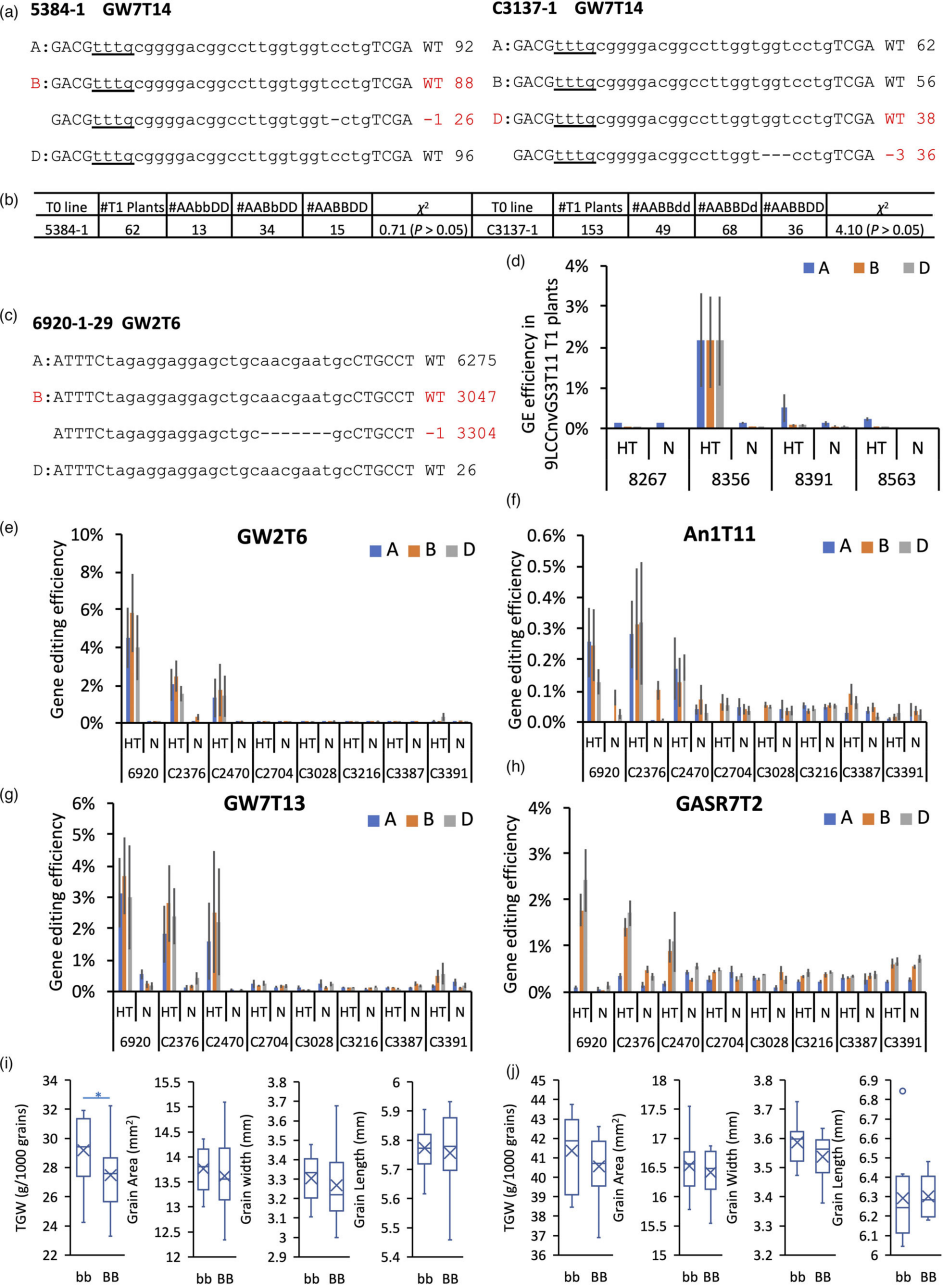
The mutations induced by the *Cas12a* MGE construct in transgenic plants and the phenotypic effects of heritable mutations on *TaGW7* gene. (a) The NGS reads generated for the target site GW7T14 in the T0 lines 5384‐1 and C3137‐1 were aligned to the homoeologous genes from the A, B and D genomes. The level of divergence between the wheat genomes allowed for separating reads to individual homoeologs during alignment. The number of wild‐type (WT) and mutated reads (shown as the number of deleted bases after ‘‐’ sign) aligned to each genome was counted and shown on the right side of each sequence. The deleted nucleotides are shown with red dashed lines. The PAM sequences are underlined. (b) The segregation of Cas12a‐induced alleles in the *TaGW7* gene in T_1_ generation. The capital letters A, B and D and the lower‐case letters a, b and d represent the wild‐type and mutated alleles of *TaGW7* in the A, B and D genomes, respectively. The χ^2^‐test shows that the ratio of mutated and wild‐type alleles is consistent with the expected 1 : 2 : 1 Mendelian segregation ratio. (c) NGS of the target site GW2T6 in the T_1_ plant 6920‐1‐29. The layout of the aligned NGS reads flanking the target site GW2T6 is similar to that in (a). (d) Gene editing efficiency in T_1_ lines carrying 9LCCnvGS3T11 before and after high‐temperature treatment. (e–h) Gene editing efficiency in T_1_ lines carrying 9LCCnv8T before and after high‐temperature treatment. The data are shown in bar plots with mean ± standard error. HT—high‐temperature treatment, N—normal growth conditions. The means are based on 3 to 21 biological replicates. (i) and (j) Box and whisker plots show the trait distribution for TGW, grain area, grain width and grain length in T_1_ (i) and T_2_ (j) progeny of line 5384‐1 with mutation in *TaGW7*. The mean value of each group is shown as an ‘x’ sign within the box plots. Only the B genome genotypes with the *Cas12a*‐induced mutations are shown. The capital letters and the lower‐case letter represent the wild‐type and mutated alleles, respectively. The T_1_ plants were derived from T_0_ line 5384‐1, with 13 plants having genotype *AAbbDD*, and 15 plants having genotype *AABBDD*. All T_2_ plants were derived from T_1_ plant 5384‐1‐3 (genotype *AABbDD*) with 10 plants having genotype *AAbbDD*, and 11 plants having genotype *AABBDD*. Student’s *t* test was applied to compare data between genotypes *AABBDD* and *AAbbDD*; *P ≤ 0.05.

To validate the CRISPR‐Cas12a system in wheat, we created 35 and 51 transgenic plants with the 9LCCnvGS3T11 (guide targeting the GS3T11 site subcloned into 9LCCnv) and 9LCCnv8T constructs, respectively. Genotyping by NGS detected no mutations in the 35 plants with 9LCCnvGS3T11. Amongst 51 with the 9LCCnv8T construct, two lines (5384‐1 and C3137‐1) had mutations at the target site GW7T14, with no mutations detected in the remaining target sites (Table S6). These results indicate the low editing efficiency of CRISPR‐Cas12a in wheat. The T_0_ plant 5384‐1 was heterozygous for 1‐bp deletion in *TaGW7‐B1*, and the T_0_ plant C3137‐1 was heterozygous for 3‐bp deletion in *TaGW7‐D1* (Figure 3a). This was confirmed by the segregation ratio of the mutated alleles in the T_1_ progeny of 5384‐1 and C3137‐1 plants (Figure 3b).

Previously, we reported that due to the transgenerational activity of CRISPR‐Cas9 new mutations could be recovered in the progeny of transgenic plants (Wang *et al*., 2018b, 2019). We genotyped by NGS all remaining seven targets (beside GW7T14) in 62 T_1_ lines of 5384‐1 and 153 T_1_ lines of C3137‐1. No new mutations were detected at any of these seven targets (Table S6).

To assess the effect of the *Cas12a* expression levels on GE in the T_1_ plants, we performed quantitative PCR analysis of *Cas12a* in 47 T_1_ lines derived from 39 T_0_ plants carrying 9LCCnv8T, and 10 T_1_ lines derived from 8 T_0_ plants carrying 9LCCnvGS3T11 (Figure S3). The T_1_ plants showing the high *Cas12a* expression levels were screened for CRISPR‐Cas12a‐induced mutations. In total, we screened 121 T_1_ plants derived from 8 T_0_ plants carrying 9LCCnv8T (*LbCas12a* expression level relative to *TaActin* ≥ 1) and 12 T_1_ plants derived from 4 T_0_ plants carrying 9LCCnvGS3T11 (*LbCas12a* expression level relative to *TaActin* ≥ 0.4). All T_0_ plants had no edited targets detected. Amongst all screened T_1_ lines, only one line derived from T_0_ plant 6920‐1 (*LbCas12a* expression level relative to *TaActin* ˜ 2.5) was shown to carry heterozygous mutation in the *TaGW2*‐*B1* gene targeted by the GW2T6 guide (Figure 3c; Table S7).

Previously, it was shown that high‐temperature treatment increases the Cas12a GE efficiency in rice, maize and Arabidopsis (Malzahn *et al*., 2019). To assess the effect of high‐temperature treatment on the GE efficiency in wheat, the selected set of T_1_ lines with highly expressed *Cas12a* was genotyped before and after the treatment. The 2‐week seedlings were treated for 2 weeks under 35 °C during the day (16 h) and 30 °C during the night (8 h). The T_1_ plants derived from one out of four T_0_ plants carrying 9LCCnvGS3T11 showed substantial improvement in GE efficiency at target site GS3T11 (Figure 3d; Table S7); the average editing efficiency increased from 0.1% before treatment to 2.2% after treatment. Likewise, after high‐temperature treatment, the T_1_ plants derived from three out of eight T_0_ plants carrying 9LCCnv8T showed improvement in GE efficiency at all multiplexed targets, except for GSE5T10 (Figure 3e‐h and Figure S4, Table S7). For example, the GE efficiency of both GW2T6 and GW7T13 targets was ˜0.3% before treatment. After treatment, the GW2T6 site showed the highest average GE efficiency of 5.8% and 2.5% in the T_1_ progeny of two T_0_ plants; GW7T13 showed the highest average GE efficiency of 2.5% in the T_1_ progeny of one T_0_ plant (Figure 3e, g and Figure S4, Table S7).

### A CRISPR‐Cas12a‐induced mutation in the *TaGW7‐B1* gene changed grain shape and weight

The effects of CRISPR‐LbCas12a‐induced mutations in *TaGW7‐B1* on grain size and thousand grain weight (TGW) were assessed in the T_1_ and T_2_ lines derived from 5384‐1. The C3137‐1 was excluded from further analyses because the 3‐bp deletion does not cause frameshift in the coding sequence. In T_1_ lines, the TGW of *TaGW7‐B1* homozygous mutants was increased by 6.2% compared to the wild‐type lines segregated from the same T_0_ plants (*t* test, *P* < 0.05; Figure 3i). This result was confirmed by the 2% increase of TGW in the *TaGW7‐B1* homozygous mutants compared to the wild‐type plants in the T_2_ generation, though difference was not statistically significant (Figure 3j). Whilst the grain length of *TaGW7‐B1* homozygous mutants was not increased compared to the wild‐type plants, the grain width was increased by 1.1% and 1.4% in the T_1_ and T_2_ lines, respectively. This increase of grain width was accompanied by the slight increase of grain area in the T_1_ and T_2_ lines. Though these increases in grain size were not statistically significant in both the T_1_ and T_2_ lines, the direction of phenotypic change in the mutants from both populations was similar, and also was consistent with the changes in the *TaGW7‐B1* mutants previously created by our group using the CRISPR‐Cas9‐based editing (Wang *et al*., 2019).

### Improving the CRISPR‐Cas12a‐based genome editing efficiency in wheat

**Figure 4:**
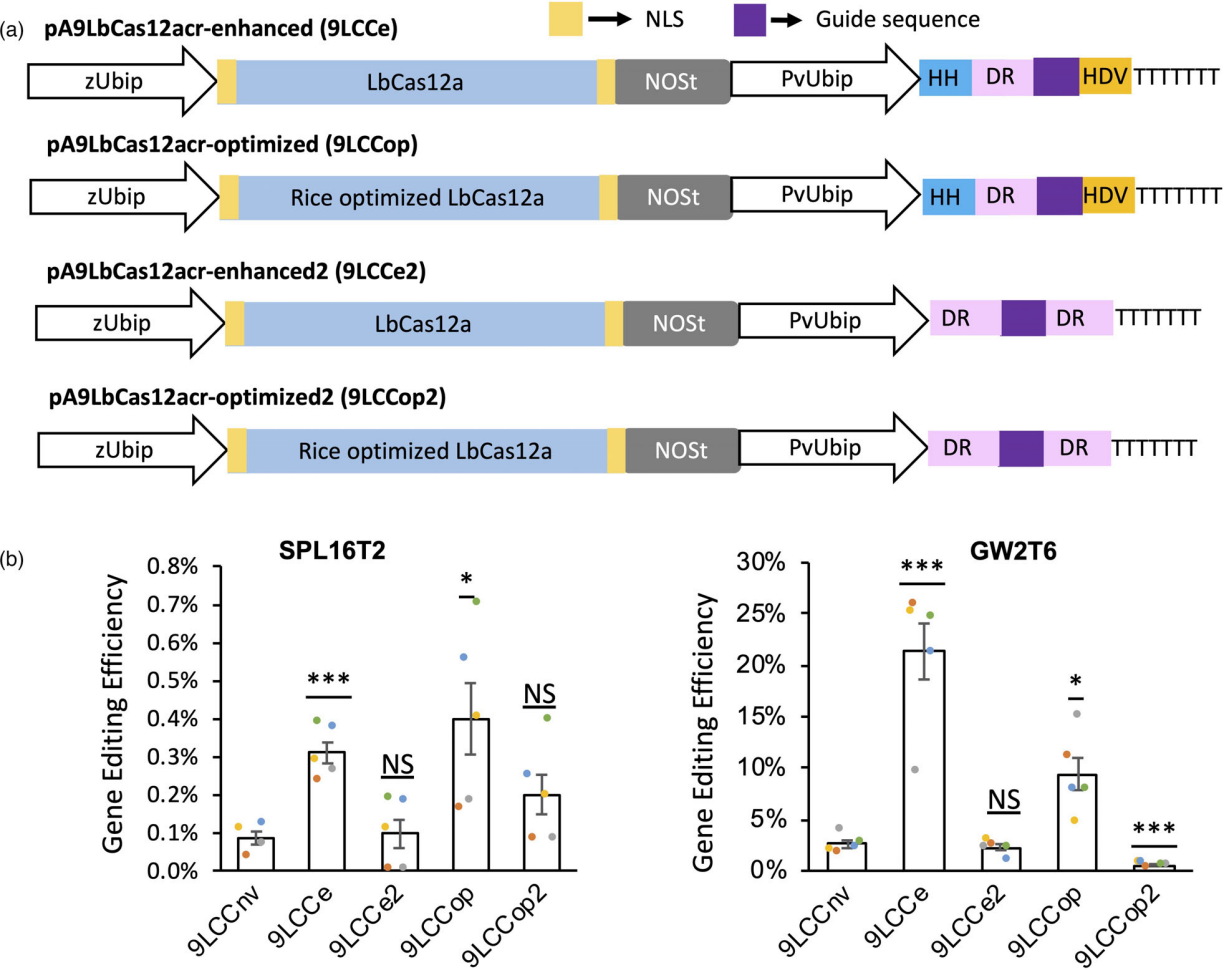
Improving the gene editing efficiency of LbCas12a. (a) Schematic illustration of the modified LbCas12a constructs. Compared to 9LCCnv, in all constructs, the wheat U6 promoter was replaced by the PvUbip promoter. 9LCCe and 9LCCop have hammerhead (HH) and hepatitis delta virus (HDV) ribozymes flanking the 5′ and 3′ ends of crRNA to facilitate its processing; 9Lcce2 and 9LCCop2 have one extra direct repeat (DR) after the 3′ end of the guide sequence; 9LCCe and 9LCCe2 have the human codon‐optimized LbCas12a whilst 9LCCop and 9LCCop2 have the plant codon‐optimized LbCas12a. (b) The comparison of gene editing efficiency amongst the LbCas12a‐based constructs. The performance of these constructs was calculated for the SPL16T2 and GW2T6 targets, which are conserved in all three wheat genomes, using data from four or five biological replicates. The mean ± standard error for each construct is shown on the graphs with the result of each replicate depicted as coloured dots on the bar plots. The gene editing efficiency was normalized by the protoplast transformation efficiency. Student’s *t* test was applied to assess the significance of differences in gene editing efficiency between 9LCCnv and 9LCCe, 9LCCe2, 9LCCop or 9LCCop2; *P ≤ 0.05; **P ≤ 0.01; ***P ≤ 0.001; ^NS^ *P* > 0.05.

The performance of the CRISPR‐LbCas12a construct 9LCCnv for most genes was significantly lower than that of the CRISPR‐Cas9 constructs (Figures 1d and 2b). Here, we explored the effect of various construct designs on the CRISPR‐LbCas12a performance. In rice, the editing efficiency of the LbCas12a system was significantly improved using the maize ubiquitin promoter to express RNA guides flanked by ribozymes (Tang *et al*., 2017). We created a construct pA9LbCas12acr‐enhanced (9LCCe) where the expression of a crRNA unit composed of direct repeat (DR) and spacer flanked by two ribozymes is driven by the switchgrass ubiquitin promoter (PvUbip; Figure 4a). Previously, it was also suggested that reduced GE efficiency of LbCas12a in rice for the last crRNA unit within the multiplex constructs could be associated with the lack of DR flanking the last spacer from the 3′ end (Wang *et al*., 2017). We evaluated whether the addition of DR to the 3′ end of the spacer could further improve GE efficiency using a construct referred to as pA9LbCas12acr‐enhanced2 (9LCCe2; Figure 4a). In addition, we compared human codon‐optimized LbCas12a with the plant codon‐optimized version (Tang *et al*., 2017) using constructs 9LCCop and 9LCCop2 (Figure 4a).

The DR and guides targeting SPL16T2 and GW2T6 were subcloned into these modified constructs (Figure 4a). Compared to the guides driven by the TaU6 promoter (9LCCnv construct), the editing efficiency of the SPL16T2 and GW2T6 crRNA flanked by ribozymes expressed from the PvUbip promoter was improved by 3‐ and 8‐fold, respectively (Figure 4b; Table S8). However, guides driven by the PvUbip promoter without the flanking ribozymes but carrying additional DR from the guides’ 3′ ends did not show improvement in editing efficiency (Figure 4b; Table S8). These results confirm the positive combined impact of the Pol II promoter and ribozyme usage on the performance of LbCas12a‐based GE system recently demonstrated in rice (Zhang *et al*., 2021). The observed improvement in GE efficiency is likely linked with the double ribozyme system rather than with the PvUbip promoter usage, because in our study, the combination of DR addition with the PvUbip promoter had little impact on editing efficiency. The plant codon‐optimized Cas12a slightly improved the GE efficiency at target SPL16T2, but no improvement was observed at target GW2T6 (Figure 4b; Table S8), which suggests that codon optimization did not substantially affect the performance of the CRISPR‐LbCas12a system.

### The LbCas12a variant with the altered PAM induces mutations in the wheat genome

**Figure 5:**
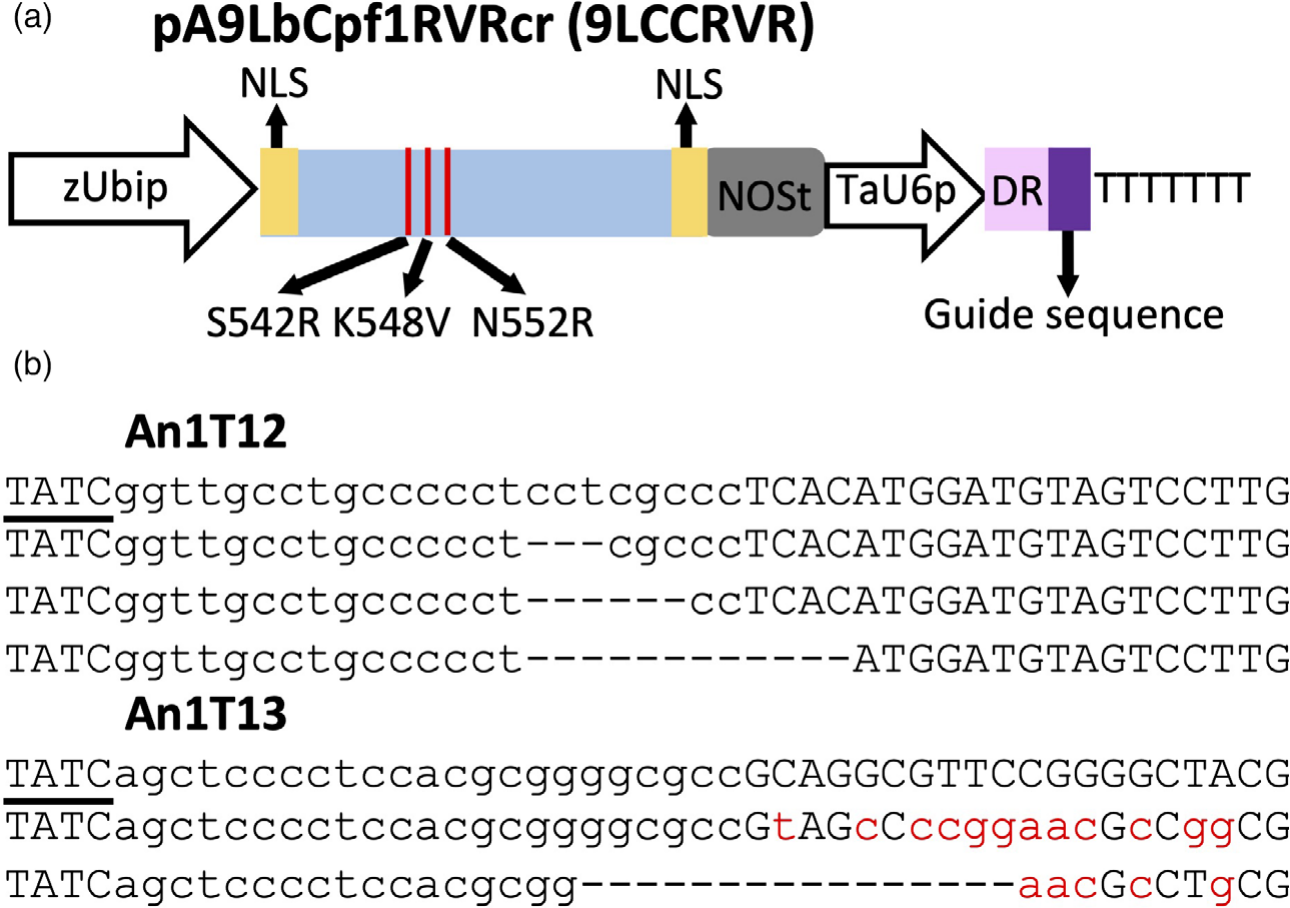
Engineered LbCas12a induces mutations in the wheat genome. (a) Schematic illustration of plasmid pA9LbCas12aRVRCr (9LCCRVR). The modified LbCas12a‐RVR contains three amino acid substitutions, S542R, K548V, and N552R, which change PAM recognition specificity to ‘TATV’. (b) The representative mutated reads of two targets on *TaAn‐1* induced by CRISPR‐ LbCas12a‐RVR. The first row for each target is wild type. The PAM sequence is underlined. The target sequences are shown in lower‐case letters; the SNPs in the mutated reads of the An1T13 site are shown as red lower‐case letters. The deleted nucleotides are shown as ‘‐’.

To broaden the editing capability of LbCas12a, we created a variant carrying mutations G532R, K538V and Y542R (henceforth LbCas12a‐RVR) that could recognize targets sites with the TATV PAMs (Figure 5a). As expected, the GE using LbCas12a‐RVR at three target sites, GSE5T9, GW2T6 and PDST16, each having the TTTV PAM, resulted in low mutation rate (Table S9). On contrary, by using LbCas12a‐RVR in combination with two guides targeting sites with the TATV PAMs in the *TaAn‐1* gene (Table S1), we detected mutations at both sites (Figure 5b) with the highest GE efficiency reaching 3.1% (Table S9).

### Off‐target gene editing activity of LbCas12a and LbCas12a‐RVR

It has been reported that both AsCas12a and LbCas12a show lower off‐target activity in human cells compared to Cas9 (Kim *et al*., 2016), likely due to the long PAM sequence. Here, we identified the possible off‐target sites for the LbCas12a by comparing the target sequences with the reference genome IWGSC RefSeq v1.0 (The International Wheat Genome Sequencing Consortium (IWGSC) 2018). Amongst the analysed 10 targets for LbCas12a and two targets for LbCas12a‐RVR, only six targets had matching PAMs (Figure S5). Most of these possible off‐target sequences were highly divergent within the 8 ˜ 10 bp from the PAM‐distal end, and one of the possible off‐target regions had SNPs located three and ten base‐pairs after PAM (Figure S5). These mutations are expected to prevent LbCas12a from inducing mutations at the off‐target sites. By sequencing these possible off‐target sites, we demonstrated the lack of off‐target editing activity for both CRISPR‐LbCas12a and CRISPR‐ LbCas12a‐RVR constructs (Table S10).

### Cas9‐NG and xCas9 expand the range of genome editing targets in wheat

**Figure 6:**
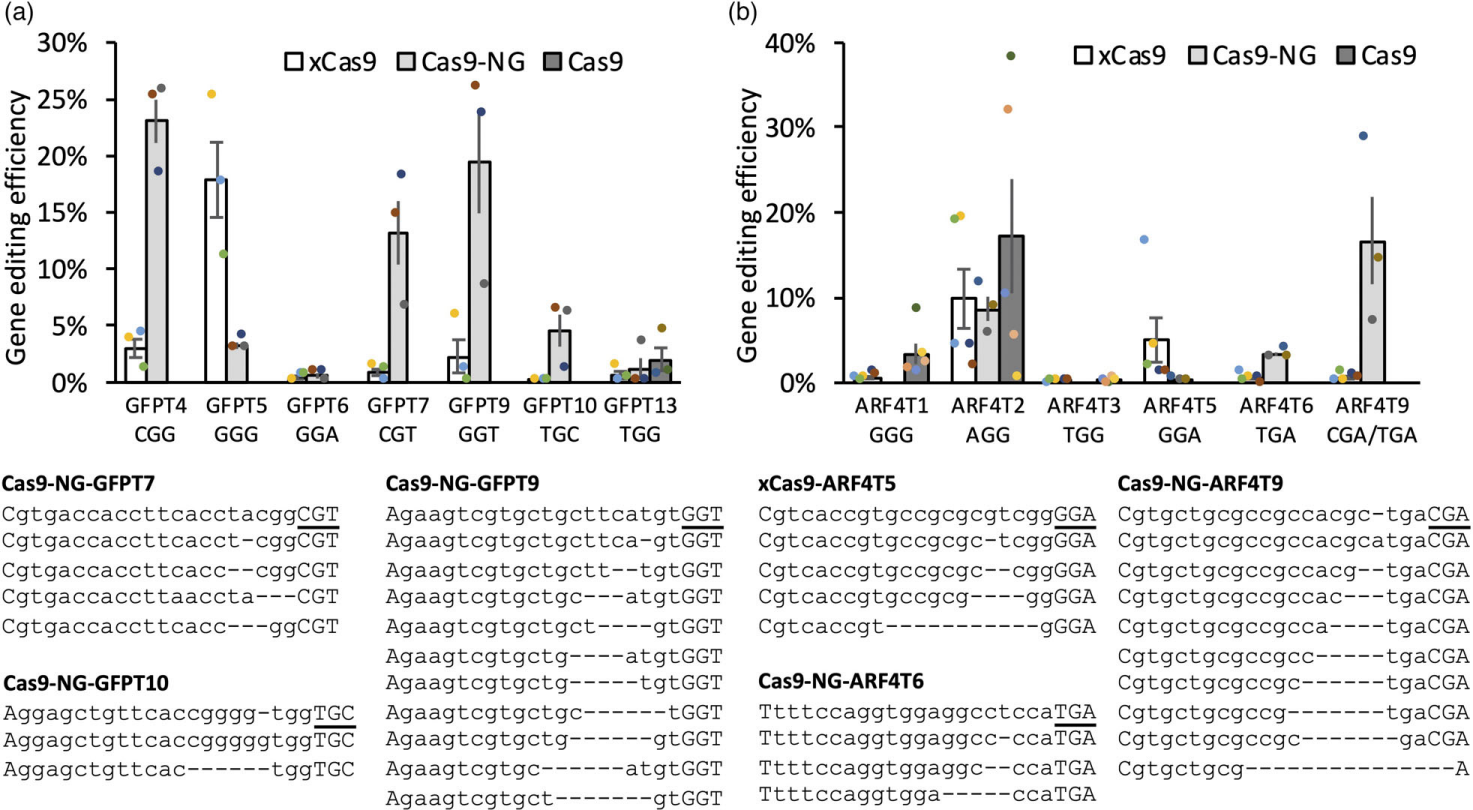
Mutations induced by the Cas9 variants in the wheat genome. (a) Comparison of the xCas9 and Cas9‐NG editing efficiency for targets located within the *GFP* gene. The calculation of gene editing efficiency are based on three biological replicates. (b) Comparison of the xCas9 and Cas9‐NG editing efficiency for targets located within the *TaARF4* genes. Because target sites were conserved across all three wheat genomes, the assessment of gene editing efficiency are based on the mapped reads pooled from all three wheat genomes. The gene editing efficiency was normalized by the protoplast transforming efficiency. The results were based on three to five biological replicates. The data in both (a) and (b) are shown as mean ± standard error, the result from each replicate is shown as coloured dots on the bar plots. The representative mutated reads for the Cas9‐NG’s targets GFPT7, GFPT9, GFPT10, ARF4T6, and ARF4T9, and the xCas9’s target ARFRT5 are shown under the bar plots. The first row for each target is wild type. The PAM sequence is underlined. The target sequences are shown in lower‐case letters. The deleted nucleotides are shown as ‘‐’.

To investigate whether the xCas9 and Cas9‐NG enzymes engineered to recognize NG PAMs could edit genes in the wheat genome, the maize codon‐optimized Cas9 in construct pBUN421 was replaced by the synthesized maize codon‐optimized xCas9 and Cas9‐NG, henceforth referred to as pBUN421x and pBUN421NG, respectively. The GE ability of xCas9 and Cas9‐NG was investigated by transforming them into the wheat protoplasts isolated from a wheat line constitutively expressing green fluorescent protein (GFP). Seven targets with the ‘NGN’ PAMs were designed for the GFP coding sequence (Table S1). Whilst both xCas9 and Cas9‐NG nucleases induced mutations in the targets with the NGG PAMs, they showed preference for different targets. Compared to xCas9, Cas9‐NG showed eight times higher editing efficiency for target GFPT4 (Figure 6a). In contrast, xCas9 showed four times higher editing efficiency than Cas9‐NG for target GFPT5 (Figure 6a). When compared to wild‐type Cas9, both xCas9 and Cas9‐NG had lower editing efficiency for target GFPT13 with the NGG PAM (Figure 6a). For all four targets with the NGH PAMs, xCas9 showed lower editing efficiency than Cas9‐NG (Figure 6a). The highest editing efficiency of 5.8% and 26% was observed for xCas9 and Cas9‐NG on target site GFPT9 (Figure 6a; Table [Link], [Link]). Whilst Cas9‐NG induced mutations in targets GFPT7 and GFPT10 with the editing efficiency of 13.2% and 4.6%, respectively, no mutations were induced by xCas9 in these targets (Figure 6a; Table [Link], [Link]).

The ability of xCas9 and Cas9‐NG to edit the wheat genome was evaluated by targeting three regions with the NGG PAMs and three regions with the NGA PAMs in the *TaARF4* gene (Table S1). Similar to target region GFPT13, both xCas9 and Cas9‐NG showed lower editing efficiency than the wild‐type Cas9 for target regions ARF4T1 and ARF4T2, both with the NGG PAM (Figure 6b; Table [Link], [Link]). Mutations were induced by xCas9 in one of the three targets with the NGA PAM, ARF4T5, with the editing efficiency of 5%. Cas9‐NG induced mutations in the two NGA PAM targets, ARF4T6 and ARF4T9, with the editing efficiency of 16.7% and 3.4%, respectively (Figure 6b; Table [Link], [Link]).

## Discussion

In the current study, we successfully applied the natural and engineered variants of the Cas12a and Cas9 nucleases for genome editing in wheat. Even though FnCas12a was reported to be effective for genome editing in rice (Zhong *et al*., 2018), FnCas12a did not induce detectable mutations in our experiments, making it unsuitable for wheat genome engineering. This observation indicates that further optimization of newly CRISPR‐Cas systems is usually required to improve their portability across species, even for those that belong to the same clade. On contrary, LbCas12a was capable of inducing double strand breaks in the wheat genome, albeit at the lower rates than those previously observed in our studies for Cas9 (Wang *et al*., 2016, 2018b, 2019). Both the LbCas12a and engineered LbCas12a‐RVR variants showed no off‐target activities and ability to support highly specific genome editing in wheat.

The recovery of mutant lines with the loss‐of‐function mutations in the *TaGW7‐B1* gene, demonstrating the expected effects on the grain size and weight traits (Wang *et al*., 2019), suggests that the Cas12a‐based editors are effective tool for modifying the wheat genome, especially in the regions that lack the NGG PAMs recognized by Cas9. To our best knowledge, this is the first report on the usage of Cas12a for producing heritable mutations in the wheat genome with the validated effect on phenotypic traits. We also found that the efficiency of Cas12a‐based MGE assessed in the protoplasts was not directly related to that obtained in the transgenic plants. Consistent with this observation, our prior work based on Cas9 showed that even though the protoplast‐based screening is critical for selecting guide RNAs for robust genome editing, the genome editing efficiencies assessed using this approach show relatively low correlation with the number of transgenic plants carrying mutations at the target loci (Wang *et al*., 2018b).

We show that a crRNA array without spacers in combination with Cas12a could be applied for the multiplexed wheat genome editing. In agreement with our previous study comparing the efficiency of simplex and MGE using CRISPR‐Cas9 in wheat (Wang *et al*., 2018b), most Cas12a targets were edited with the efficiency equal to that of the simplex guide RNAs. In addition, in the wheat protoplasts, we observed increase in the GW2T6 target editing efficiency following the increase in the level of multiplexing. Whilst this trend could be explained by various factors, including the increased Cas12a recruiting capacity of the crRNA arrays with the higher levels of multiplexing, the position of protospacers within the crRNA array or the higher accessibility of this locus in the wheat genome, it is currently hard to discern a specific reason for this observation.

Contrary to earlier work conducted in rice (Xing *et al*., 2014), the codon optimization of LbCas12a did not improve the efficiency of wheat genome editing. However, modifications introduced into the guide RNA‐processing system had a substantial effect on the LbCas12a’s editing ability. Even though the Cas12a nuclease is capable of processing crRNA arrays to produce mature crRNAs (Fonfara *et al*., 2016), its natural crRNA processing capability appears to limit the efficiency of genome editing in wheat, which could be substantially enhanced by supplementing crRNA constructs with ribozymes (Tang *et al*., 2017). Although most Cas12a targets had editing efficiency lower than 5%, some exhibited high mutation rates, as high as 24%, indicating that genome editing efficiency could be further improved by optimizing the CRISPR‐LbCas12a system. Consistent with a previous study (Malzahn *et al*., 2019), our results showed that the low editing activity exhibited by Cas12a under normal plant growth conditions could be overcome by high‐temperature treatment. The improvement of the Cas12a tolerance to low temperature was proposed as another strategy for increasing its editing activity. The potential of this approach was successfully demonstrated for LbCas12a (Schindele and Puchta, 2020) and Mb2Cas12a (Zhang *et al*., 2021). Further studies are warranted to assess the utility of these approaches for editing the wheat genome.

The engineered variants of Cas12a and Cas9 (LbCas12a‐RVR, Cas9‐NG and xCas9) recognizing non‐canonical PAMs (TATV and NG) further expand the scope of editable loci in the wheat genome. The application of engineered Cas12a variants to plant genome editing was until recently limited (Zhong *et al*., 2018). The successful modification of the targets located next to the TATC PAM using the altered LbCas12a‐RVR nuclease provides great addition to the wheat genome editing toolbox and broaden the range of species whose genomes could be modified using LbCas12a. We showed that the xCas9 and Cas9‐NG nucleases targeting minimal NG PAM were effective at generating double strand breaks within the endogenous gene targets that could not be edited using other editors. Though both xCas9 and Cas9‐NG recognized targets with the NG PAMs, in wheat, we observed some bias in the target preference, with Cas9‐NG being more effective at targets followed by the NGH PAM than xCas9 (Zeng *et al*., 2019; Zhong *et al*., 2019). Compared to xCas9 and Cas9‐NG, the recently engineered near‐PAMless SpCas9 variants SpG and SpRY, which recognize sites with non‐canonical NGN and NAN PAMs and many sites with NCN and NTN PAMs, have demonstrated the ability to target even broader range of genomic sites (Walton *et al*., 2020). The optimization of these nucleases for wheat has potential to fully eliminate constraints imposed by PAM recognition in genome editing applications.

## Conclusion

Here, we evaluated the ability of the natural (FnCas12a, LbCas12a) and engineered (LbCas12a‐RVR, xCas9 and Cas9‐NG) variants of the CRISPR‐based DNA editors to induce mutations within the endogenous gene targets with the canonical and altered PAMs in the complex wheat genome. We demonstrated the improved target editing efficiency in the wheat genome using the LbCas12a constructs with crRNA units flanked by ribozymes. We showed that the multiplex gene editing efficiency mediated by LbCas12a substantially increases after subjecting transgenic plants to high‐temperature treatment. By using the LbCas12a nuclease in combination with the multiplexed RNA guides, we created stable wheat mutants with increased grain size and weight. We showed that the scope of editable loci in the wheat genome could be expanded by using the engineered LbCas12a‐RVR, xCas9 and Cas9‐NG nucleases recognizing targets with altered PAMs. Our study also highlights the importance of the systematic testing and optimization of newly developed CRISPR‐Cas‐based genome editing technologies to create a crop‐specific customized toolkits to effectively implement diverse genome editing strategies for improving agronomic traits.

## Experimental procedures

### Plasmid construction

To construct the plasmids for this study, all the DNA oligoes and fragments were synthesized by Integrated DNA Technologies. All the PCR was performed using NEBNext^®^ High‐Fidelity 2× PCR Master Mix (Catalogue number: M0541L, New England Biolabs Inc., Ipswich, MA USA) following the manufacturer’s instructions. All DNA fragments were assembled using the NEBuilder^®^ HiFi DNA Assembly Cloning Kit (Catalogue number: E5520S, New England Biolabs) following the manufacturer’s instructions. All the newly constructed plasmids were confirmed by Sanger sequencing.

A plasmid with AsCas12a crRNA (CRISPR RNA of Cas12a from *Acidaminococcus* sp. *BV3l6*) expression cassette was firstly constructed. The NOS terminator and wheat U6 promoter in plasmid pA9Cas9sg (Wang *et al*., 2016, 2019) were amplified using primers NOS‐F and DR‐AsCas12aF1 (Table S12). The direct repeat sequence of AsCas12a crRNA, two BsaI cutting sites, and seven ‘T’ bases were added by the second round of PCR using the NOS‐F and DR‐AsCas12aF2 primers (Table S12). The final PCR products were subcloned into pA9FeYFP (Figure S1) between the XmaI and SacI cutting sites by replacing the eYFP and NOS terminator. Henceforth, the resulting construct is referred to as pA9Ascr.

To construct plasmid pA9LbCas12acr (9LCC for short) that expressed the human codon‐optimized LbCas12a and crRNA, the plasmid pA9Ascr was amplified using the primer pair LbCrF and LbCrR. The PCR product was self‐ligated using NEBuilder^®^ HiFi DNA Assembly Cloning Kit, and henceforth is referred to as pA9LbCr. The human codon‐optimized LbCas12a CDS and the 3′ end NLS were amplified using the LbCas12aF and LbCas12aR primers from plasmid pY016 (pcDNA3.1‐hLbCas12a). The PCR product was subcloned into pA9Lbcr to create the plasmid 9LCC (Figure S1). To add one more NLS peptide to the 5′ end of human codon‐optimized LbCas12a CDS, a primer pair SV40NLS_LbCas12a_F and LbCas12aR was used to amplify the human codon‐optimized LbCas12a CDS and the 3′ end NLS. Then the resulting PCR product was amplified again using a primer pair pA9_SV40NLS_F and LbCas12aR, followed by subcloning into pA9Lbcr to create a plasmid pA9LbCas12acr‐new‐version, or 9LCCnv for short (Figure S1). To create plasmid pA9 LbCas12aRVRcr, the plasmid pA9LbCas12acr was amplified using a primer pair pA9_SV40NLS_F and LbCas12aRVR‐R, and a primer pair LbCas12aRVR‐F and LbCas12aR. The resulting PCR products were ligated and amplified using a primer pair pA9_SV40NLS_F and LbCas12aR followed by subcloning into KpnI and XmaI digested pA9LbCas12acr‐new‐version to replace the wild‐type Cas12a.

To construct pA9FnCas12acr (9FCC for short) expressing a wheat codon‐optimized FnCas12a and its crRNA, the FnCas12a coding sequence with NLS and 3× HA tag on the 3′ ends was synthesized (Figure S1), and amplified using a primer pair SV40NLS_FnCas12a_F and FnCas12aR (Table S12). The resulting DNA fragment was amplified again using a primer pair pA9_SV40NLS_F and FnCas12aR to finalize the addition of SV40 NLSs. The PCR product was subcloned into pA9Ascr between KpnI and XmaI cut sites, resulting in plasmid pA9FnCas12aAscr. To change the crRNA direct repeat sequence from AsCas12a to FnCas12a, the plasmid was amplified using two primer pairs: NOS‐F and FnCrR, and FnCrF and FnCas12aR. The resulting two PCR products were assembled. This created the plasmid 9FCC.

To construct the pA9LbCas12a‐enhanced (9LCCe for short), the hammerhead ribozyme and hepatitis delta virus ribozyme sequences were added to the 5′ and 3′ ends of LbCas12a crRNA direct repeat, respectively, using the two rounds of PCR. Two BsaI cutting sites were embedded between the crRNA direct repeat and delta virus ribozyme. The first round of PCR was conducted using the primer pair Hammer‐crF and HDVribo‐crR with plasmid 9LCC as a template. The second PCR was done using the primer pair Hammer‐F and HDVriboR. The PCR product was assembled with PstI + XhoI digested pMOD_B2312 (Cermak *et al*., 2017). The new construct, designated as pMODLBcr, was then amplified using the primer pair PvUbi1pF4 and 35SterR2, and the 9LCC was amplified using the primer pair NOS‐F5 and NOS‐R followed by digestion with BamHI. The first PCR product and the digested product were assembled and amplified again using primer NOS‐F5 and 35SterR2. The new PCR product was assembled together with the 9LCC construct digested with XmaI and SacI to generate plasmid 9LCCe, which was then amplified using the two primer pairs, PvUbi1pF4 and PvUbi1pR1, and 35SterF2 and 35SterR2. These two PCR products were assembled and amplified using the primer pair PvUbi1pF4 and 35SterR2. The new PCR product was assembled with SpeI and SacI digested 9LCCe to form the plasmid pA9LbCas12a‐enhanced2 (9LCCe2 for short). Then the plant codon‐optimized LbCas12a was amplified from plasmid pYPQ230 (Tang *et al*., 2017) and used to replace the human codon‐optimized LbCas12a in 9LCCe and 9LCCe2, henceforth pA9LbCas12a‐optimized (9LCCop for short) and pA9LbCas12a‐optimized2 (9LCCop2 for short), respectively.

To obtain xCas9 and Cas9‐NG constructs, mutations were introduced into the maize codon‐optimized zCas9 in plasmid pBUN421 (Xing *et al*., 2014), resulting in the pBUN421x and pBUN421NG constructs, respectively. To construct pBUN421x, zCas9 was amplified with the two primer pairs, zCas9F3 and zCas9seq4, and zCas9R3 and zCas9seq5. Both PCR products were assembled along with the synthesized DNA fragment zxCas9_741‐3740. The assembled DNA fragment was amplified using the pair of primers zCas9F3 and zCas9R3, followed by assembling the resulting PCR product with XmaI and StuI digested pBUN421 to replace the wild‐type Cas9. To construct pBUN421NG, zCas9 was amplified using the primers zCas9F3 and zCas9seq19, and the resulting product was assembled along with the synthesized DNA fragment zCas9‐NG_R. The assembled product was amplified using the primers zCas9F3 and zCas9R3, followed by assembling the PCR product with XmaI and StuI digested pBUN421 to replace the wild‐type Cas9.

The sequences of the wheat orthologues of six rice genes, including *OsGW2* (Song *et al*., 2007), *OsGS3* (Mao *et al*., 2010), *OsGSE5* (Duan *et al*., 2017), *OsAn‐1* (Luo *et al*., 2013), *OsSPL16* (Wang *et al*., 2012), *OsARF4* (Hu *et al*., 2018b), were identified by comparing with the IWGSC RefSeq v2.0 reference genome on Ensembl Plants (https://plants.ensembl.org/index.html). The crRNA protospacers were selected from the cDNA sequences of the *TaPDS*, *TaGASR7* (Ling *et al*., 2013), *TaGW2*, *TaGS3*, *TaGSE5*, *TaAn‐1*, *TaSPL16* and *TaGW7* genes (Wang *et al*., 2019). Both forward and reverse sequences of the protospacers along with the 4‐nucleotide 5′ overhangs were synthesized and subcloned into pA9FnCas12acr, pA9LbCas12acr, pA9 LbCas12aRVRcr, pBUN421x and pBUN421NG, as previously described (Wang *et al*., 2018b). To create the MGE constructs with three, four or eight tandem crRNA units, we synthesized one or two ultra‐DNA oligonucleotides (Table S12). These oligonucleotides were assembled and amplified by PCR, and resulting amplicons were subcloned into plasmid 9LCCnv.

### Protoplast transformation

The wheat protoplast transient expression assay was performed as previously described with some modifications (Wang *et al*., 2018b). About 100 seedlings of wheat cultivar Bobwhite were grown in the dark for 2 weeks, shoot tissues were finely sliced and vacuumed at −600 mbar for 30 min in a 30 mL of W5 solution (0.1% glucose, 0.08% KCl, 0.9% NaCl, 1.84% CaCl_2_·2H_2_O, 2 mM MES‐KOH, pH 5.7). Then the tissues were digested for 2.5 h in a 30 mL enzymic mix containing 1.5% Cellulase R10 (from *Trichoderma viride*, 7.5 U/mg), 0.75% Macerozyme R10 (from *Rhizopus* sp.), 0.6 m mannitol, 10 mm MES pH 5.7, 10 mm CaCl_2_ and 0.1% BSA. After digestion, protoplasts were filtered through 40 μm nylon meshes. The remaining tissues were washed with the 30 mL of W5 solution followed by filtering through the nylon meshes. The resulting cell suspension was mixed gently, and protoplasts were collected by centrifugation at 100 ***g*** for 5 min, and then washed twice with the 10 mL of W5 solution. The final protoplast pellet was re‐suspended in the 5 mL of W5 solution, cell was counted using a haemocytometer. The re‐suspended protoplasts were kept on ice for 30 min to allow for the natural sedimentation. Then protoplast cell count was adjusted to 10^6^ cells/mL in MMG solution (0.4 m mannitol, 15 mm MgCl_2_, 4 mm MES, pH 5.7).

The 10 µg of plasmid DNA and 100 µL of protoplasts were mixed with the 130 µL of PEG solution (40% (W/V) PEG 4000, 0.2 m mannitol and 0.1 m CaCl_2_). After 30‐min incubation at room temperature in the dark, 500 µL of W5 solution was added. The protoplasts were collected by centrifugation at 100 ***g*** for 2 min, re‐suspended in 1 mL of W5 solution and incubated in the dark at room temperature. The transformation efficiency was assessed by counting the fraction of fluorescent‐positive protoplasts transformed with pA9mRFP. Protoplasts were collected 48 h after transformation and DNA was isolated with PureLink Genomic DNA Mini Kit (Thermo Fisher Scientific, Catalogue number: K182002) following the manufacture’s protocol.

### Gene editing efficiency calculation by the next generation sequencing (NGS) of PCR amplicon library

To detect mutations induced by the CRISPR‐Cas12a or CRISPR‐Cas9 variants, genomic regions harbouring the crRNA targets were amplified by PCR. The Illumina’s TruSeq adaptors on both ends of the amplicons were added using two rounds of PCR as described (Wang *et al*., 2016). PCR products were purified with MinElute PCR Purification Kit (Qiagen), pooled in equimolar ratio, and sequenced on a MiSeq Sequencer using the MiSeq Reagent Nano Kit v2 (500 cycles, 2 × 250 bp run) at the K‐State Integrated Genomics Facility. The Illumina reads passing quality control were aligned to the wild‐type reference sequences of targeted genes from each homoeologous chromosome separately. The GE efficiency was calculated for each homoeologous gene target by dividing the total number of mutated reads by the total number of all aligned reads.

### Transgenic plants regeneration and genotyping

Wheat immature embryo transformation and plant regeneration were performed as previously described (Saintenac *et al*., 2013). To isolate DNA, leaf tissues were sampled and homogenized in 500 μL of TPS buffer, then incubated for 20 min at 75 °C. After centrifugation for 5 min, 140 μL of the supernatant was mixed with 140 μL isopropanol and incubated for 20 min at room temperature. DNA was precipitated, washed with 70% ethanol and re‐suspended in 100 μL of deionized water.

The presence of CRISPR/Cas12a or CRISPR‐Cas9 variants constructs in the transgenic plants was validated by PCR using four pairs of primers amplifying different regions of the Cas12a and crRNA expression cassettes (Table S12). The CRISPR/Cas12a‐induced mutations were examined only in the plants showing the presence of three PCR products. The mutations were detected using the NGS‐based procedure described above.

### Plant growth and high‐temperature treatment

The CRISPR‐Cas12a induced mutant plants were grown and phenotyped as described previously with slight modifications (Wang *et al*., 2018c). Briefly, the T1 generation plants were grown in a growth chamber under 16‐h light/8‐h dark cycle. The temperature was set to 24 °C during the day and 20 °C during the night. The T_2_ generation plants were grown in the Kansas State University’s greenhouses under natural conditions supplemented by additional light sources to maintain 16‐h light/8‐h dark cycle. The room temperature was set as 24 °C during the day and 20 °C during the night.

To better evaluate the GE activity of CRISPR‐Cas12a in the transgenic lines, five or more seeds derived from each tiller of T_0_ plants carrying 9LCCnvGS3T11 and 9LCCnv8T were planted into the 16 × 8 well plastic tray (277.9 mm × 544.6 mm × 32.0 mm). Plants were grown under normal conditions as described above. The DNA and RNA were isolated from the leaves collected from the 2‐week seedlings (Wang *et al*., 2019). The presence of CRISPR‐Cas12a was tested by PCR using primers LB_25_F and LB_197_R. The RNA of one Cas12a positive T_1_ line from each T_0_ plant tiller was isolated and used to assess the expression levels of *Cas12a* by qPCR using primers LB_25_F/LB_197_R and Power SYBR™ Green PCR Master Mix (Thermo Fisher Scientific, catalogue# 4368706). T_1_ plants with the highest *Cas12a* expression level derived from 4 T_0_ plants carrying 9LCCnvGS3T11 and 8 T_0_ plants carrying 9LCCnv8T were selected for high‐temperature treatment. The plants were transplanted into the 1 L square pots and grown for 2 weeks in the growth chamber at 35 °C during the day (16 h) and 30 °C during the night (8 h). The NGS‐based genotyping of CRISPR‐Cas12a targets was performed with DNA isolated from the leaves of high‐temperature treated plants.

### Grain morphometric data collection

To collect the grain morphometric data, three main spikes from each plant were harvested separately. A MARVIN seed analyzer (GTA Sensorik GmbH, Germany) was used to measure the grain morphometric traits (grain width, length, area), and TGW for each spike. The mean of three spikes from each plant was calculated and used for further analyses.

### Statistical analysis

The two‐tailed Student’s *t* test was applied to assess the significance of differences between the simplex and multiplex GE efficiencies in the wheat protoplasts.

## Conflict of interest

The authors declared that they do not have conflict of interests.

## Author contributions

W.W. designed and conducted gene editing experiments, genotyped and phenotyped transgenic plants, collected and analysed phenotyping data and drafted the manuscript; B.T. conducted plant transformation experiments, provided the transgenic wheat with GFP, helped to design the gene editing experiments; Q.P. analysed gene editing events using NGS; Y.C. performed biolistic transformation of wheat embryos; F.H. developed NGS analysis pipeline; G.B. performed Sanger sequencing; A.A. designed and conducted NGS experiments; H.T. coordinated biolistic transformation part of the project; E.A. conceived idea, interpreted results, coordinated project and wrote the manuscript. All authors read the manuscript and approved the final version.

## Supporting information


**Figure S1** The sequences of plasmids used in this study.

**Figure S2** Gene editing efficiency comparison between the single‐target and multiple‐target CRISPR‐LbCas12a constructs.

**Figure S3** LbCas12a expression level in T_1_ progeny of (a) 9LCCnv8T and (b) 9LCCnvGS3T11 transgenic plants.

**Figure S4** The gene editing efficiency in MGE transgenic plants with and without high‐temperature treatment.

**Figure S5** The alignment of off‐target sequences with 12 LbCpf1 targets selected based on Chinese Spring RefSeqv1.0.

**Table S1** The targets of Cas12a and Cas9 variants designed in this study

**Table S2** The NGS‐based gene editing efficiency analyses of CRISPR‐LbCas12a constructs in wheat protoplasts

**Table S3** The NGS‐based gene editing efficiency analyses of CRISPR‐FnCas12a constructs in wheat protoplasts

**Table S4** The NGS‐based gene editing efficiency analyses of CRISPR‐Cas9 constructs in wheat protoplasts

**Table S5** The NGS‐based gene editing efficiency analyses LbCas12a multiplex constructs in wheat protoplasts

**Table S6** The NGS‐based genotyping of T0 and T1 plant of 5384‐1 and C3137‐1

**Table S7** The NGS‐based genotyping of T1 plants grown under normal room temperature

**Table S8** The NGS‐based gene editing efficiency analyses of improved LbCas12a constructs in wheat protoplasts

**Table S9** The NGS‐based gene editing efficiency analyses of LbCas12a‐RVR constructs in wheat protoplasts

**Table S10** The NGS‐based off‐target analyses of LbCas12a and LbCas12a‐RVR constructs

**Table S11** The NGS‐based gene editing efficiency analyses of Cas9 variants in wheat protoplasts

**Table S12** The primers and DNA oligoes used in this study

Legends S1
