# Supplementary material for: Expanding the range of editable targets in the wheat genome using the variants of the Cas12a and Cas9 nucleases
Source: Plant Biotechnol J. 2021 Jul 28;19(12):2428–41. doi: 10.1111/pbi.13669 (PMC8633491; doi:10.1111/pbi.13669)
Supplement: Supplementary file 1 — Figure S1 The sequences of plasmids used in this study. [file PBI-19-2428-s010.docx]

**pA9FeYFP (9Y for short)**

GTAATCATGTCATAGCTGTTTCCTGTGTGAAATTGTTATCCGCTCACAATTCCACACAACATACGAGCCGGAAGCATAAAGTGTAAAGCCTGGGGTGCCTAATGAGTGAGCTAACTCACATTAATTGCGTTGCGCTCACTGCCCGCTTTCCAGTCGGGAAACCTGTCGTGCCAGCTGCATTAATGAATCGGCCAACGCGCGGGGAGAGGCGGTTTGCGTATTGGGCGCTCTTCCGCTTCCTCGCTCACTGACTCGCTGCGCTCGGTCGTTCGGCTGCGGCGAGCGGTATCAGCTCACTCAAAGGCGGTAATACGGTTATCCACAGAATCAGGGGATAACGCAGGAAAGAACATGTGAGCAAAAGGCCAGCAAAAGGCCAGGAACCGTAAAAAGGCCGCGTTGCTGGCGTTTTTCCATAGGCTCCGCCCCCCTGACGAGCATCACAAAAATCGACGCTCAAGTCAGAGGTGGCGAAACCCGACAGGACTATAAAGATACCAGGCGTTTCCCCCTGGAAGCTCCCTCGTGCGCTCTCCTGTTCCGACCCTGCCGCTTACCGGATACCTGTCCGCCTTTCTCCCTTCGGGAAGCGTGGCGCTTTCTCATAGCTCACGCTGTAGGTATCTCAGTTCGGTGTAGGTCGTTCGCTCCAAGCTGGGCTGTGTGCACGAACCCCCCGTTCAGCCCGACCGCTGCGCCTTATCCGGTAACTATCGTCTTGAGTCCAACCCGGTAAGACACGACTTATCGCCACTGGCAGCAGCCACTGGTAACAGGATTAGCAGAGCGAGGTATGTAGGCGGTGCTACAGAGTTCTTGAAGTGGTGGCCTAACTACGGCTACACTAGAAGGACAGTATTTGGTATCTGCGCTCTGCTGAAGCCAGTTACCTTCGGAAAAAGAGTTGGTAGCTCTTGATCCGGCAAACAAACCACCGCTGGTAGCGGTGGTTTTTTTGTTTGCAAGCAGCAGATTACGCGCAGAAAAAAAGGATCTCAAGAAGATCCTTTGATCTTTTCTACGGGGTCTGACGCTCAGTGGAACGAAAACTCACGTTAAGGGATTTTGGTCATGAGATTATCAAAAAGGATCTTCACCTAGATCCTTTTAAATTAAAAATGAAGTTTTAAATCAATCTAAAGTATATATGAGTAAACTTGGTCTGACAGTTACCAATGCTTAATCAGTGAGGCACCTATCTCAGCGATCTGTCTATTTCGTTCATCCATAGTTGCCTGACTCCCCGTCGTGTAGATAACTACGATACGGGAGGGCTTACCATCTGGCCCCAGTGCTGCAATGATACCGCGAGACCCACGCTCACCGGCTCCAGATTTATCAGCAATAAACCAGCCAGCCGGAAGGGCCGAGCGCAGAAGTGGTCCTGCAACTTTATCCGCCTCCATCCAGTCTATTAATTGTTGCCGGGAAGCTAGAGTAAGTAGTTCGCCAGTTAATAGTTTGCGCAACGTTGTTGCCATTGCTACAGGCATCGTGGTGTCACGCTCGTCGTTTGGTATGGCTTCATTCAGCTCCGGTTCCCAACGATCAAGGCGAGTTACATGATCCCCCATGTTGTGCAAAAAAGCGGTTAGCTCCTTCGGTCCTCCGATCGTTGTCAGAAGTAAGTTGGCCGCAGTGTTATCACTCATGGTTATGGCAGCACTGCATAATTCTCTTACTGTCATGCCATCCGTAAGATGCTTTTCTGTGACTGGTGAGTACTCAACCAAGTCATTCTGAGAATAGTGTATGCGGCGACCGAGTTGCTCTTGCCCGGCGTCAATACGGGATAATACCGCGCCACATAGCAGAACTTTAAAAGTGCTCATCATTGGAAAACGTTCTTCGGGGCGAAAACTCTCAAGGATCTTACCGCTGTTGAGATCCAGTTCGATGTAACCCACTCGTGCACCCAACTGATCTTCAGCATCTTTTACTTTCACCAGCGTTTCTGGGTGAGCAAAAACAGGAAGGCAAAATGCCGCAAAAAAGGGAATAAGGGCGACACGGAAATGTTGAATACTCATACTCTTCCTTTTTCAATATTATTGAAGCATTTATCAGGGTTATTGTCTCATGAGCGGATACATATTTGAATGTATTTAGAAAAATAAACAAATAGGGGTTCCGCGCACATTTCCCCGAAAAGTGCCACCTGACGTCTAAGAAACCATTATTATCATGACATTAACCTATAAAAATAGGCGTATCACGAGGCCCTTTCGTCTCGCGCGTTTCGGTGATGACGGTGAAAACCTCTGACACATGCAGCTCCCGGAGACGGTCACAGCTTGTCTGTAAGCGGATGCCGGGAGCAGACAAGCCCGTCAGGGCGCGTCAGCGGGTGTTGGCGGGTGTCGGGGCTGGCTTAACTATGCGGCATCAGAGCAGATTGTACTGAGAGTGCACCATATGCGGTGTGAAATACCGCACAGATGCGTAAGGAGAAAATACCGCATCAGGCGCCATTCGCCATTCAGGCTGCGCAACTGTTGGGAAGGGCGATCGGTGCGGGCCTCTTCGCTATTACGCCAGCTGGCGAAAGGGGGATGTGCTGCAAGGCGATTAAGTTGGGTAACGCCAGGGTTTTCCCAGTCACGACGTTGTAAAACGACGGCCAGTGCC**aagcttGCATGCCTGCAG**tgcagcgtgacccggtcgtgcccctctctagagataatgagcattgcatgtctaagttataaaaaattaccacatattttttttgtcacacttgtttgaagtgcagtttatctatctttatacatatatttaaactttactctacgaataatataatctatagtactacaataatatcagtgttttagagaatcatataaatgaacagttagacatggtctaaaggacaattgagtattttgacaacaggactctacagttttatctttttagtgtgcatgtgttctcctttttttttgcaaatagcttcacctatataatacttcatccattttattagtacatccatttagggtttagggttaatggtttttatagactaatttttttagtacatctattttattctattttagcctctaaattaagaaaactaaaactctattttagtttttttatttaataatttagatataaaatagaataaaataaagtgactaaaaattaaacaaataccctttaagaaattaaaaaaactaaggaaacatttttcttgtttcgagtagataatgccagcctgttaaacgccgtcgacgagtctaacggacaccaaccagcgaaccagcagcgtcgcgtcgggccaagcgaagcagacggcacggcatctctgtcgctgcctctggacccctctcgagagttccgctccaccgttggacttgctccgctgtcggcatccagaaattgcgtggcggagcggcagacgtgagccsgcacggcaggcggcctcctcctcctctcacggcaccggcagctacgggggattcctttcccaccgctccttcgctttcccttcctcgcccgccgtaataaatagacaccccctccacaccctctttccccaacctcgtgttgttcggagcgcacacacacacaaccagatctcccccaaatccacccgtcggcacctccgcttcaaggtacgccgctcgtcctccccccccccccctctctaccttctctagatcggcgttccggtccatggttagggcccggtagttctacttctgttcatgtttgtgttagatccgtgtttgtgttagatccgtgctgctagcgttcgtacacggatgcgacctgtacgtcagacacgttctgattgctaacttgccagtgtttctctttggggaatcctgggatggctctagccgttccgcagacgggatcgatttcatgattttttttgtttcgttgcatagggtttggtttgcccttttcctttatttcaatatatgccgtgcacttgtttgtcgggtcatcttttcatgcttttttttgtcttggttgtgatgatgtggtctggttgggcggtcgttctagatcggagtagaattctgtttcaaactacctggtggatttattaattttggatctgtatgtgtgtgccatacatattcatagttacgaattgaagatgatggatggaaatatcgatctaggataggtatacatgttgatgcgggttttactgatgcatatacagagatgctttttgttcgcttggttgtgatgatgtggtgtggttgggcggtcgttcattcgttctagatcggagtagaatactgtttcaaactacctggtgtatttattaattttggaactgtatgtgtgtgtcatacatcttcatagttacgagtttaagatggatggaaatatcgatctaggataggtatacatgttgatgtgggttttactgatgcatatacatgatggcatatgcagcatctattcatatgctctaaccttgagtacctatctattataataaacaagtatgttttataattattttgatcttgatatacttggatgatggcatatgcagcagctatatgtggatttttttagccctgccttcatacgctatttatttgcttggtactgtttcttttgtcgatgctcaccctgttgtttggtgttacttctgcaggtcgacTCTAGAggatccTAggtaccGGTTAACGCGTTAATTAAcccgggTTTAAACACTAGTATGGTGAGCAAGGGCGAGGAGCTGTTCACCGGGGTGGTGCCCATCCTGGTCGAGCTGGACGGCGACGTAAACGGCCACAAGTTCAGCGTGTCCGGCGAGGGCGAGGGCGATGCCACCTACGGCAAGCTGACCCTGAAGTTCATCTGCACCACCGGCAAGCTGCCCGTGCCCTGGCCCACCCTCGTGACCACCTTCGGCTACGGCCTGCAGTGCTTCGCCCGCTACCCCGACCACATGAAGCAGCACGACTTCTTCAAGTCCGCCATGCCCGAAGGCTACGTCCAGGAGCGCACCATCTTCTTCAAGGACGACGGCAACTACAAGACCCGCGCCGAGGTGAAGTTCGAGGGCGACACCCTGGTGAACCGCATCGAGCTGAAGGGCATCGACTTCAAGGAGGACGGCAACATCCTGGGGCACAAGCTGGAGTACAACTACAACAGCCACAACGTCTATATCATGGCCGACAAGCAGAAGAACGGCATCAAGGTGAACTTCAAGATCCGCCACAACATCGAGGACGGCAGCGTGCAGCTCGCCGACCACTACCAGCAGAACACCCCCATCGGCGACGGCCCCGTGCTGCTGCCCGACAACCACTACCTGAGCTACCAGTCCGCCCTGAGCAAAGACCCCAACGAGAAGCGCGATCACATGGTCCTGCTGGAGTTCGTGACCGCCGCCGGGATCACTCTCGGCATGGACGAGCTGTACAAGTAAgagctc*GAATTTCCCCGATCGTTCAAACATTTGGCAATAAAGTTTCTTAAGATTGAATCCTGTTGCCGGTCTTGCGATGATTATCATATAATTTCTGTTGAATTACGTTAAGCATGTAATAATTAACATGTAATGCATGACGTTATTTATGAGATGGGTTTTTATGATTAGAGTCCCGCAATTATACATTTAATACGCGATAGAAAACAAAATATAGCGCGCAAACTAGGATAAATTATCGCGCGCGGTGTCATCTATGTTACTAGATCGG*gaattc

All the plasmids with a name started with “pA9” were constructed using the pUC19 as a backbone. The maize ubiquitin promoter is shown as underlined **lowercase** letters. The CDS of enhanced Yellow Fluorescent Signal shown as yellow capital letters. The NOS terminator is shown as italic capital letters. The enzyme cut sites are all shown in lower case. KpnI is shown in red, EcoRI is shown in purple, XmaI is shown in yellow, SacI is shown in green, BamHI is shown in light blue.

**pA9Cas9sg**

aagctt**GCATGCCTGCAG**tgcagcgtgacccggtcgtgcccctctctagagataatgagcattgcatgtctaagttataaaaaattaccacatattttttttgtcacacttgtttgaagtgcagtttatctatctttatacatatatttaaactttactctacgaataatataatctatagtactacaataatatcagtgttttagagaatcatataaatgaacagttagacatggtctaaaggacaattgagtattttgacaacaggactctacagttttatctttttagtgtgcatgtgttctcctttttttttgcaaatagcttcacctatataatacttcatccattttattagtacatccatttagggtttagggttaatggtttttatagactaatttttttagtacatctattttattctattttagcctctaaattaagaaaactaaaactctattttagtttttttatttaataatttagatataaaatagaataaaataaagtgactaaaaattaaacaaataccctttaagaaattaaaaaaactaaggaaacatttttcttgtttcgagtagataatgccagcctgttaaacgccgtcgacgagtctaacggacaccaaccagcgaaccagcagcgtcgcgtcgggccaagcgaagcagacggcacggcatctctgtcgctgcctctggacccctctcgagagttccgctccaccgttggacttgctccgctgtcggcatccagaaattgcgtggcggagcggcagacgtgagccsgcacggcaggcggcctcctcctcctctcacggcaccggcagctacgggggattcctttcccaccgctccttcgctttcccttcctcgcccgccgtaataaatagacaccccctccacaccctctttccccaacctcgtgttgttcggagcgcacacacacacaaccagatctcccccaaatccacccgtcggcacctccgcttcaaggtacgccgctcgtcctccccccccccccctctctaccttctctagatcggcgttccggtccatggttagggcccggtagttctacttctgttcatgtttgtgttagatccgtgtttgtgttagatccgtgctgctagcgttcgtacacggatgcgacctgtacgtcagacacgttctgattgctaacttgccagtgtttctctttggggaatcctgggatggctctagccgttccgcagacgggatcgatttcatgattttttttgtttcgttgcatagggtttggtttgcccttttcctttatttcaatatatgccgtgcacttgtttgtcgggtcatcttttcatgcttttttttgtcttggttgtgatgatgtggtctggttgggcggtcgttctagatcggagtagaattctgtttcaaactacctggtggatttattaattttggatctgtatgtgtgtgccatacatattcatagttacgaattgaagatgatggatggaaatatcgatctagcataggtatacatgttgatgcgggttttactgatgcatatacagagatgctttttgttcgcttggttgtgatgatgtggtgtggttgggcggtcgttcattcgttctagatcggagtagaatactgtttcaaactacctggtgtatttattaattttggaactgtatgtgtgtgtcatacatcttcatagttacgagtttaagatggatggaaatatcgatctaggataggtatacatgttgatgtgggttttactgatgcatatacatgatggcatatgcagcatctattcatatgctctaaccttgagtacctatctattataataaacaagtatgttttataattattttgatcttgatatacttggatgatggcatatgcagcagctatatgtggatttttttagccctgccttcatacgctatttatttgcttggtactgtttcttttgtcgatgctcaccctgttgtttggtgttactt**CTGCAGGTCGACTCTAGAGGATCTCGAGatggcacctaaaaaaaagaggaaggtggggatacatggggtgccagctgcaatggataaaaagtactctatcgggctcgatatcggcactaactccgtggggtgggccgtcatcacagacgaatacaaggtcccatcgaaaaaatttaaagttttgggcaataccgacagacactctatcaaaaagaacctgattggagcgcttttgtttgattctggagaaacggccgaagccacccggctcaagcggaccgcccgtcggcgctatactcgtcggaagaaccgcatttgttaccttcaggagatcttttcaaatgagatggctaaggtggacgattctttctttcaccggctggaggagtccttccttgtcgaagaggacaaaaaacacgagaggcatccaatattcggcaacattgtggatgaagtggcatatcacgagaagtaccccaccatataccatcttagaaagaaacttgtcgactccacggacaaggccgaccttcgtctgatttacctcgcgctcgcccatatgatcaagtttcggggccatttcctcatcgagggagacttgaatcctgacaattctgacgtcgacaaattgtttatccaactcgtgcaaacgtacaaccaactctttgaggagaacccaataaacgcctccggagttgatgcaaaagctatcctgagcgcgcgcctttcgaagtcccggaggctggagaacttgatcgcccaactcccaggcgaaaagaagaacggcttgtttggcaatcttattgccctgagcctcgggcttacacccaactttaagtcaaacttcgacctggcagaggatgctaagctccagctctcgaaggacacctacgacgacgatttggacaacctcttggcccaaatcggcgatcagtatgccgatttgttcctcgccgccaaaaacctctcggatgcgattctgctctctgacatcttgcgcgtgaacaccgagataacgaaggcgccgctgtctgcgtccatgatcaagcggtacgacgagcaccatcaggacctgaccctccttaaggcgttggtgcgccaacaattgcccgagaagtataaggaaatcttcttcgatcaatccaagaacggctacgccggatacatagacggaggggcctcccaggaggagttctataagtttatcaagcctattcttgaaaagatggatggcaccgaggagctcctcgttaaacttaacagggaggaccttctccgtaagcaaagaaccttcgataatgggtcaataccccaccaaatccacctcggagagctccacgcgatcctcagacggcaggaggatttttatccattcctcaaagataaccgtgagaaaatcgaaaagatcctgaccttccgcatcccatactacgtcgggcctttggcgcggggtaacagcagattcgcctggatgaccaggaaatcagaagagacaataaccccgtggaacttcgaagaggttgttgacaagggtgcgtccgcacagtctttcatcgaaaggatgactaactttgacaaaaaccttcccaatgagaaagtgctccctaagcattccctgttgtatgagtacttcaccgtctacaacgagttgaccaaagtcaagtacgtcacagaggggatgcgcaagccagcctttctcagcggcgagcaaaagaaggctattgttgacctcttgttcaagacgaacaggaaggtcaccgtgaagcagctgaaggaggattactttaagaagatagagtgcttcgactcggtggagatttcaggggtcgaggaccgcttcaatgcctcacttgggacataccatgacctgcttaagattatcaaggacaaggactttctggacaatgaagagaacgaggacatcttggaggacattgtcctcacactcacgctcttcgaagaccgggaaatgattgaagagagactcaagacttatgcgcacttgtttgatgacaaggtgatgaagcaactcaagaggcgcaggtacacaggctggggccgcctctcccgtaagctcatcaatggcatccgtgacaagcagtccggaaagactatattggactttctgaagtccgacggtttcgccaaccggaatttcatgcaacttatccacgatgactctctgacattcaaggaagatatccaaaaggcccaggtgagcggacagggcgactctctgcatgagcacatcgcgaaccttgccggatccccggccatcaagaagggaatccttcagaccgtgaaggttgtcgatgagcttgttaaggtgatgggcaggcacaagcctgagaacattgtcatagagatggcccgtgagaatcagacaacgcagaagggccagaagaactccagagagcgtatgaaaagaattgaagaagggattaaggagttgggctcacaaatcctgaaggagcacccagtcgagaatacgcagctgcagaacgagaaactgtatctgtattacctgcagaacggacgcgacatgtatgtcgaccaggaactcgatatcaatagactcagcgattatgacgtcgaccatatcgtgcctcaatcgttcctcaaggatgactccatcgataacaaggtgctcacgagatcggacaagaatcgcggcaaatccgacaacgtcccgagcgaggaggttgtcaagaaaatgaagaattactggaggcaactgcttaacgcaaagctgatcacccagagaaagttcgacaatctcacgaaggcagagcggggtggactttctgaactggataaggcagggtttattaaacgccagctggtggagacacgtcagatcacaaagcatgtggcgcagattctggacagccggatgaatacgaagtatgacgagaacgataagctgatcagggaggtgaaagttatcacattgaagtccaagctcgtgtccgattttcggaaggacttccagttttataaggttagggagattaacaactatcaccacgctcacgacgcctatctgaatgcggttgttggcacggcactcattaaaaagtaccccaagctggagtccgagttcgtgtacggtgactacaaggtgtacgacgtgcgcaagatgattgctaagtctgaacaagagataggcaaggcaaccgccaagtactttttctactccaacataatgaactttttcaaaaccgagataactcttgcgaatggcgagatccgcaaacggccacttatcgagacgaatggtgagactggagagatcgtgtgggacaagggacgggattttgcgactgttaggaaggtgctttccatgccgcaagtgaacatagtgaagaaaacagaggttcaaacgggaggctttagcaaggaatccatcctccccaagaggaattccgataagctcatagctagaaaaaaggactgggacccaaagaagtatggcgggtttgactcaccgaccgtcgcgtactcggttctcgtcgttgcgaaggttgaaaagggtaagtctaaaaagctcaaatcagtgaaggagctcctgggcattaccatcatggagaggtcatcatttgagaagaatccaatcgatttccttgaggccaaggggtacaaggaggtgaagaaggacctgataatcaagctgcctaagtattcactcttcgaactcgaaaacgggaggaagaggatgctggcatccgctggagagcttcagaagggaaatgaactcgcccttccatccaagtacgtcaacttcctttacctcgcctcgcattacgagaagctcaagggttcccctgaagacaatgagcaaaaacagctcttcgtggagcaacataagcattacctggacgagattatcgaacagatctccgagttttccaagcgtgtcatcctcgctgacgctaatctcgacaaggttctctccgcctacaacaaacaccgggacaagccaatccgggagcaagccgaaaatattatccacctcttcacccttactaatctcggggcacctgcggcgtttaaatactttgacaccacgatagaccgcaagcgctatacgtccaccaaggaggttcttgacgcgaccctcattcaccagagcatcacaggcctctacgaaacgaggatcgacctcagccagctgggtggggataaaagacccgcggcaactaagaaagccggccaagcgaaaaagaagaaatga**cccggg**GAATTTCCCCGATCGTTCAAACATTTGGCAATAAAGTTTCTTAAGATTGAATCCTGTTGCCGGTCTTGCGATGATTATCATATAATTTCTGTTGAATTACGTTAAGCATGTAATAATTAACATGTAATGCATGACGTTATTTATGAGATGGGTTTTTATGATTAGAGTCCCGCAATTATACATTTAATACGCGATAGAAAACAAAATATAGCGCGCAAACTAGGATAAATTATCGCGCGCGGTGTCATCTATGTTACTAGATCGGGGATCCGACCAAGCCCGTTATTCTGACAGTTCTGGTGCTCAACACATTTATATTTATCAAGGAGCACATTGTTACTCACTGCTAGGAGGGAATCGAACTAGGAATATTGATCAGAGGAACTACGAGAGAGCTGAAGATAACTGCCCTCTAGCTCTCACTGATCTGGGTCGCATAGTGAGATGCAGCCCACGTGAGTTCAGCAACGGTCTAGCGCTGGGCTTTTAGGCCCGCATGATCGGGCTTTTGTCGGGTGGTCGACGTGTTCACGATTGGGGAGAGCAACGCAGCAGTTCCTCTTAGTTTAGTCCCACCTCGCCTGTCCAGCAGAGTTCTGACCGGTTTATAAACTCGCTTGCTGCATCAGACTTGAGAGACCGAGAGAGGGTCTCAGTTTTAGAGCTAGAAATAGCAAGTTAAAATAAGGCTAGTCCGTTATCAACTTGAAAAAGTGGCACCGAGTCGGTGCTTTTTTT**gagctcgaattcactggccgtcgttttacaacgtcgtgactgggaaaaccctggcgttacccaacttaatcgccttgcagcacatccccctttcgccagctggcgtaatagcgaagaggcccgcaccgatcgcccttcccaacagttgcgcagcctgaatggcgaatggcgcctgatgcggtattttctccttacgcatctgtgcggtatttcacaccgcatatggtgcactctcagtacaatctgctctgatgccgcatagttaagccagccccgacacccgccaacacccgctgacgcgccctgacgggcttgtctgctcccggcatccgcttacagacaagctgtgaccgtctccgggagctgcatgtgtcagaggttttcaccgtcatcaccgaaacgcgcgagacgaaagggcctcgtgatacgcctatttttataggttaatgtcatgataataatggtttcttagacgtcaggtggcacttttcggggaaatgtgcgcggaacccctatttgtttatttttctaaatacattcaaatatgtatccgctcatgagacaataaccctgataaatgcttcaataatattgaaaaaggaagagtatgagtattcaacatttccgtgtcgcccttattcccttttttgcggcattttgccttcctgtttttgctcacccagaaacgctggtgaaagtaaaagatgctgaagatcagttgggtgcacgagtgggttacatcgaactggatctcaacagcggtaagatccttgagagttttcgccccgaagaacgttttccaatgatgagcacttttaaagttctgctatgtggcgcggtattatcccgtattgacgccgggcaagagcaactcggtcgccgcatacactattctcagaatgacttggttgagtactcaccagtcacagaaaagcatcttacggatggcatgacagtaagagaattatgcagtgctgccataaccatgagtgataacactgcggccaacttacttctgacaacgatcggaggaccgaaggagctaaccgcttttttgcacaacatgggggatcatgtaactcgccttgatcgttgggaaccggagctgaatgaagccataccaaacgacgagcgtgacaccacgatgcctgtagcaatggcaacaacgttgcgcaaactattaactggcgaactacttactctagcttcccggcaacaattaatagactggatggaggcggataaagttgcaggaccacttctgcgctcggcccttccggctggctggtttattgctgataaatctggagccggtgagcgtggctctcgcggtatcattgcagcactggggccagatggtaagccctcccgtatcgtagttatctacacgacggggagtcaggcaactatggatgaacgaaatagacagatcgctgagataggtgcctcactgattaagcattggtaactgtcagaccaagtttactcatatatactttagattgatttaaaacttcatttttaatttaaaaggatctaggtgaagatcctttttgataatctcatgaccaaaatcccttaacgtgagttttcgttccactgagcgtcagaccccgtagaaaagatcaaaggatcttcttgagatcctttttttctgcgcgtaatctgctgcttgcaaacaaaaaaaccaccgctaccagcggtggtttgtttgccggatcaagagctaccaactctttttccgaaggtaactggcttcagcagagcgcagataccaaatactgttcttctagtgtagccgtagttaggccaccacttcaagaactctgtagcaccgcctacatacctcgctctgctaatcctgttaccagtggctgctgccagtggcgataagtcgtgtcttaccgggttggactcaagacgatagttaccggataaggcgcagcggtcgggctgaacggggggttcgtgcacacagcccagcttggagcgaacgacctacaccgaactgagatacctacagcgtgagctatgagaaagcgccacgcttcccgaagggagaaaggcggacaggtatccggtaagcggcagggtcggaacaggagagcgcacgagggagcttccagggggaaacgcctggtatctttatagtcctgtcgggtttcgccacctctgacttgagcgtcgatttttgtgatgctcgtcaggggggcggagcctatggaaaaacgccagcaacgcggcctttttacggttcctggccttttgctggccttttgctcacatgttctttcctgcgttatcccctgattctgtggataaccgtattaccgcctttgagtgagctgataccgctcgccgcagccgaacgaccgagcgcagcgagtcagtgagcgaggaagcggaagagcgcccaatacgcaaaccgcctctccccgcgcgttggccgattcattaatgcagctggcacgacaggtttcccgactggaaagcgggcagtgagcgcaacgcaattagctcactcattaggcaccccaggctttacactttatgcttccggctcgtatgttgtgtggaattgtgagcggataacaatttcacacaggaaacagctatgaccatgattacgcc

The maize ubiquitin promoter is shown as underlined **lowercase** letters, the wheat codon optimized Cas9 CDS is highlighted in yellow, the nuclear localization signal sequences flanking Cas9 from both ends are shown in red, the NOS poly A terminator is underlined. The wheat U6 promoter (TaU6p) is shown in blue color; the transcription start site at nucleotide G is highlighted by the light green background; the gRNA sequence is shown in red color. Two BsaI cut sites for target insertion are underlined, the ploy-T for transcription termination are shown in purple.

**pA9LbCas12acr (9LCC for short)**

aagcttGCATGCCTGCAGtgcagcgtgacccggtcgtgcccctctctagagataatgagcattgcatgtctaagttataaaaaattaccacatattttttttgtcacacttgtttgaagtgcagtttatctatctttatacatatatttaaactttactctacgaataatataatctatagtactacaataatatcagtgttttagagaatcatataaatgaacagttagacatggtctaaaggacaattgagtattttgacaacaggactctacagttttatctttttagtgtgcatgtgttctcctttttttttgcaaatagcttcacctatataatacttcatccattttattagtacatccatttagggtttagggttaatggtttttatagactaatttttttagtacatctattttattctattttagcctctaaattaagaaaactaaaactctattttagtttttttatttaataatttagatataaaatagaataaaataaagtgactaaaaattaaacaaataccctttaagaaattaaaaaaactaaggaaacatttttcttgtttcgagtagataatgccagcctgttaaacgccgtcgacgagtctaacggacaccaaccagcgaaccagcagcgtcgcgtcgggccaagcgaagcagacggcacggcatctctgtcgctgcctctggacccctctcgagagttccgctccaccgttggacttgctccgctgtcggcatccagaaattgcgtggcggagcggcagacgtgagccggcacggcaggcggcctcctcctcctctcacggcaccggcagctacgggggattcctttcccaccgctccttcgctttcccttcctcgcccgccgtaataaatagacaccccctccacaccctctttccccaacctcgtgttgttcggagcgcacacacacacaaccagatctcccccaaatccacccgtcggcacctccgcttcaaggtacgccgctcgtcctccccccccccccctctctaccttctctagatcggcgttccggtccatggttagggcccggtagttctacttctgttcatgtttgtgttagatccgtgtttgtgttagatccgtgctgctagcgttcgtacacggatgcgacctgtacgtcagacacgttctgattgctaacttgccagtgtttctctttggggaatcctgggatggctctagccgttccgcagacgggatcgatttcatgattttttttgtttcgttgcatagggtttggtttgcccttttcctttatttcaatatatgccgtgcacttgtttgtcgggtcatcttttcatgcttttttttgtcttggttgtgatgatgtggtctggttgggcggtcgttctagatcggagtagaattctgtttcaaactacctggtggatttattaattttggatctgtatgtgtgtgccatacatattcatagttacgaattgaagatgatggatggaaatatcgatctaggataggtatacatgttgatgcgggttttactgatgcatatacagagatgctttttgttcgcttggttgtgatgatgtggtgtggttgggcggtcgttcattcgttctagatcggagtagaatactgtttcaaactacctggtgtatttattaattttggaactgtatgtgtgtgtcatacatcttcatagttacgagtttaagatggatggaaatatcgatctaggataggtatacatgttgatgtgggttttactgatgcatatacatgatggcatatgcagcatctattcatatgctctaaccttgagtacctatctattataataaacaagtatgttttataattattttgatcttgatatacttggatgatggcatatgcagcagctatatgtggatttttttagccctgccttcatacgctatttatttgcttggtactgtttcttttgtcgatgctcaccctgttgtttggtgttacttctgcaggtcgacTCTAGAggatccTAggtaccgccacc*ATGAGCAAGCTGGAGAAGTTTACAAACTGCTACTCCCTGTCTAAGACCCTGAGGTTCAAGGCCATCCCTGTGGGCAAGACCCAGGAGAACATCGACAATAAGCGGCTGCTGGTGGAGGACGAGAAGAGAGCCGAGGATTATAAGGGCGTGAAGAAGCTGCTGGATCGCTACTATCTGTCTTTTATCAACGACGTGCTGCACAGCATCAAGCTGAAGAATCTGAACAATTACATCAGCCTGTTCCGGAAGAAAACCAGAACCGAGAAGGAGAATAAGGAGCTGGAGAACCTGGAGATCAATCTGCGGAAGGAGATCGCCAAGGCCTTCAAGGGCAACGAGGGCTACAAGTCCCTGTTTAAGAAGGATATCATCGAGACAATCCTGCCAGAGTTCCTGGACGATAAGGACGAGATCGCCCTGGTGAACAGCTTCAATGGCTTTACCACAGCCTTCACCGGCTTCTTTGATAACAGAGAGAATATGTTTTCCGAGGAGGCCAAGAGCACATCCATCGCCTTCAGGTGTATCAACGAGAATCTGACCCGCTACATCTCTAATATGGACATCTTCGAGAAGGTGGACGCCATCTTTGATAAGCACGAGGTGCAGGAGATCAAGGAGAAGATCCTGAACAGCGACTATGATGTGGAGGATTTCTTTGAGGGCGAGTTCTTTAACTTTGTGCTGACACAGGAGGGCATCGACGTGTATAACGCCATCATCGGCGGCTTCGTGACCGAGAGCGGCGAGAAGATCAAGGGCCTGAACGAGTACATCAACCTGTATAATCAGAAAACCAAGCAGAAGCTGCCTAAGTTTAAGCCACTGTATAAGCAGGTGCTGAGCGATCGGGAGTCTCTGAGCTTCTACGGCGAGGGCTATACATCCGATGAGGAGGTGCTGGAGGTGTTTAGAAACACCCTGAACAAGAACAGCGAGATCTTCAGCTCCATCAAGAAGCTGGAGAAGCTGTTCAAGAATTTTGACGAGTACTCTAGCGCCGGCATCTTTGTGAAGAACGGCCCCGCCATCAGCACAATCTCCAAGGATATCTTCGGCGAGTGGAACGTGATCCGGGACAAGTGGAATGCCGAGTATGACGATATCCACCTGAAGAAGAAGGCCGTGGTGACCGAGAAGTACGAGGACGATCGGAGAAAGTCCTTCAAGAAGATCGGCTCCTTTTCTCTGGAGCAGCTGCAGGAGTACGCCGACGCCGATCTGTCTGTGGTGGAGAAGCTGAAGGAGATCATCATCCAGAAGGTGGATGAGATCTACAAGGTGTATGGCTCCTCTGAGAAGCTGTTCGACGCCGATTTTGTGCTGGAGAAGAGCCTGAAGAAGAACGACGCCGTGGTGGCCATCATGAAGGACCTGCTGGATTCTGTGAAGAGCTTCGAGAATTACATCAAGGCCTTCTTTGGCGAGGGCAAGGAGACAAACAGGGACGAGTCCTTCTATGGCGATTTTGTGCTGGCCTACGACATCCTGCTGAAGGTGGACCACATCTACGATGCCATCCGCAATTATGTGACCCAGAAGCCCTACTCTAAGGATAAGTTCAAGCTGTATTTTCAGAACCCTCAGTTCATGGGCGGCTGGGACAAGGATAAGGAGACAGACTATCGGGCCACCATCCTGAGATACGGCTCCAAGTACTATCTGGCCATCATGGATAAGAAGTACGCCAAGTGCCTGCAGAAGATCGACAAGGACGATGTGAACGGCAATTACGAGAAGATCAACTATAAGCTGCTGCCCGGCCCTAATAAGATGCTGCCAAAGGTGTTCTTTTCTAAGAAGTGGATGGCCTACTATAACCCCAGCGAGGACATCCAGAAGATCTACAAGAATGGCACATTCAAGAAGGGCGATATGTTTAACCTGAATGACTGTCACAAGCTGATCGACTTCTTTAAGGATAGCATCTCCCGGTATCCAAAGTGGTCCAATGCCTACGATTTCAACTTTTCTGAGACAGAGAAGTATAAGGACATCGCCGGCTTTTACAGAGAGGTGGAGGAGCAGGGCTATAAGGTGAGCTTCGAGTCTGCCAGCAAGAAGGAGGTGGATAAGCTGGTGGAGGAGGGCAAGCTGTATATGTTCCAGATCTATAACAAGGACTTTTCCGATAAGTCTCACGGCACACCCAATCTGCACACCATGTACTTCAAGCTGCTGTTTGACGAGAACAATCACGGACAGATCAGGCTGAGCGGAGGAGCAGAGCTGTTCATGAGGCGCGCCTCCCTGAAGAAGGAGGAGCTGGTGGTGCACCCAGCCAACTCCCCTATCGCCAACAAGAATCCAGATAATCCCAAGAAAACCACAACCCTGTCCTACGACGTGTATAAGGATAAGAGGTTTTCTGAGGACCAGTACGAGCTGCACATCCCAATCGCCATCAATAAGTGCCCCAAGAACATCTTCAAGATCAATACAGAGGTGCGCGTGCTGCTGAAGCACGACGATAACCCCTATGTGATCGGCATCGATAGGGGCGAGCGCAATCTGCTGTATATCGTGGTGGTGGACGGCAAGGGCAACATCGTGGAGCAGTATTCCCTGAACGAGATCATCAACAACTTCAACGGCATCAGGATCAAGACAGATTACCACTCTCTGCTGGACAAGAAGGAGAAGGAGAGGTTCGAGGCCCGCCAGAACTGGACCTCCATCGAGAATATCAAGGAGCTGAAGGCCGGCTATATCTCTCAGGTGGTGCACAAGATCTGCGAGCTGGTGGAGAAGTACGATGCCGTGATCGCCCTGGAGGACCTGAACTCTGGCTTTAAGAATAGCCGCGTGAAGGTGGAGAAGCAGGTGTATCAGAAGTTCGAGAAGATGCTGATCGATAAGCTGAACTACATGGTGGACAAGAAGTCTAATCCTTGTGCAACAGGCGGCGCCCTGAAGGGCTATCAGATCACCAATAAGTTCGAGAGCTTTAAGTCCATGTCTACCCAGAACGGCTTCATCTTTTACATCCCTGCCTGGCTGACATCCAAGATCGATCCATCTACCGGCTTTGTGAACCTGCTGAAAACCAAGTATACCAGCATCGCCGATTCCAAGAAGTTCATCAGCTCCTTTGACAGGATCATGTACGTGCCCGAGGAGGATCTGTTCGAGTTTGCCCTGGACTATAAGAACTTCTCTCGCACAGACGCCGATTACATCAAGAAGTGGAAGCTGTACTCCTACGGCAACCGGATCAGAATCTTCCGGAATCCTAAGAAGAACAACGTGTTCGACTGGGAGGAGGTGTGCCTGACCAGCGCCTATAAGGAGCTGTTCAACAAGTACGGCATCAATTATCAGCAGGGCGATATCAGAGCCCTGCTGTGCGAGCAGTCCGACAAGGCCTTCTACTCTAGCTTTATGGCCCTGATGAGCCTGATGCTGCAGATGCGGAACAGCATCACAGGCCGCACCGACGTGGATTTTCTGATCAGCCCTGTGAAGAACTCCGACGGCATCTTCTACGATAGCCGGAACTATGAGGCCCAGGAGAATGCCATCCTGCCAAAGAACGCCGACGCCAATGGCGCCTATAACATCGCCAGAAAGGTGCTGTGGGCCATCGGCCAGTTCAAGAAGGCCGAGGACGAGAAGCTGGATAAGGTGAAGATCGCCATCTCTAACAAGGAGTGGCTGGAGTACGCCCAGACCAGCGTGAAGCACAAAAGGCCGGCGGCCACGAAAAAGGCCGGCCAGGCAAAAAAGAAAAAGGGATCCtacccatacgatgttccagattacgcttatccctacgacgtgcctgattatgcatacccatatgatgtccccgactatgccTAA*cccggg**GAATTTCCCCGATCGTTCAAACATTTGGCAATAAAGTTTCTTAAGATTGAATCCTGTTGCCGGTCTTGCGATGATTATCATATAATTTCTGTTGAATTACGTTAAGCATGTAATAATTAACATGTAATGCATGACGTTATTTATGAGATGGGTTTTTATGATTAGAGTCCCGCAATTATACATTTAATACGCGATAGAAAACAAAATATAGCGCGCAAACTAGGATAAATTATCGCGCGCGGTGTCATCTATGTTACTAGATCGGGGATCCGACCAAGCCCGTTATTCTGACAGTTCTGGTGCTCAACACATTTATATTTATCAAGGAGCACATTGTTACTCACTGCTAGGAGGGAATCGAACTAGGAATATTGATCAGAGGAACTACGAGAGAGCTGAAGATAACTGCCCTCTAGCTCTCACTGATCTGGGTCGCATAGTGAGATGCAGCCCACGTGAGTTCAGCAACGGTCTAGCGCTGGGCTTTTAGGCCCGCATGATCGGGCTTTTGTCGGGTGGTCGACGTGTTCACGATTGGGGAGAGCAACGCAGCAGTTCCTCTTAGTTTAGTCCCACCTCGCCTGTCCAGCAGAGTTCTGACCGGTTTATAAACTCGCTTGCTGCATCAGACTTG**TAATTTCTACTAAGTGTAGAT**AGAGACCGAGAGAGGGTCTCATTTTTTT**gagctcgaattcactggccgtcgttttacaacgtcgtgactgggaaaaccctggcgttacccaacttaatcgccttgcagcacatccccctttcgccagctggcgtaatagcgaagaggcccgcaccgatcgcccttcccaacagttgcgcagcctgaatggcgaatggcgcctgatgcggtattttctccttacgcatctgtgcggtatttcacaccgcatatggtgcactctcagtacaatctgctctgatgccgcatagttaagccagccccgacacccgccaacacccgctgacgcgccctgacgggcttgtctgctcccggcatccgcttacagacaagctgtgaccgtctccgggagctgcatgtgtcagaggttttcaccgtcatcaccgaaacgcgcgagacgaaagggcctcgtgatacgcctatttttataggttaatgtcatgataataatggtttcttagacgtcaggtggcacttttcggggaaatgtgcgcggaacccctatttgtttatttttctaaatacattcaaatatgtatccgctcatgagacaataaccctgataaatgcttcaataatattgaaaaaggaagagtatgagtattcaacatttccgtgtcgcccttattcccttttttgcggcattttgccttcctgtttttgctcacccagaaacgctggtgaaagtaaaagatgctgaagatcagttgggtgcacgagtgggttacatcgaactggatctcaacagcggtaagatccttgagagttttcgccccgaagaacgttttccaatgatgagcacttttaaagttctgctatgtggcgcggtattatcccgtattgacgccgggcaagagcaactcggtcgccgcatacactattctcagaatgacttggttgagtactcaccagtcacagaaaagcatcttacggatggcatgacagtaagagaattatgcagtgctgccataaccatgagtgataacactgcggccaacttacttctgacaacgatcggaggaccgaaggagctaaccgcttttttgcacaacatgggggatcatgtaactcgccttgatcgttgggaaccggagctgaatgaagccataccaaacgacgagcgtgacaccacgatgcctgtagcaatggcaacaacgttgcgcaaactattaactggcgaactacttactctagcttcccggcaacaattaatagactggatggaggcggataaagttgcaggaccacttctgcgctcggcccttccggctggctggtttattgctgataaatctggagccggtgagcgtggctctcgcggtatcattgcagcactggggccagatggtaagccctcccgtatcgtagttatctacacgacggggagtcaggcaactatggatgaacgaaatagacagatcgctgagataggtgcctcactgattaagcattggtaactgtcagaccaagtttactcatatatactttagattgatttaaaacttcatttttaatttaaaaggatctaggtgaagatcctttttgataatctcatgaccaaaatcccttaacgtgagttttcgttccactgagcgtcagaccccgtagaaaagatcaaaggatcttcttgagatcctttttttctgcgcgtaatctgctgcttgcaaacaaaaaaaccaccgctaccagcggtggtttgtttgccggatcaagagctaccaactctttttccgaaggtaactggcttcagcagagcgcagataccaaatactgttcttctagtgtagccgtagttaggccaccacttcaagaactctgtagcaccgcctacatacctcgctctgctaatcctgttaccagtggctgctgccagtggcgataagtcgtgtcttaccgggttggactcaagacgatagttaccggataaggcgcagcggtcgggctgaacggggggttcgtgcacacagcccagcttggagcgaacgacctacaccgaactgagatacctacagcgtgagctatgagaaagcgccacgcttcccgaagggagaaaggcggacaggtatccggtaagcggcagggtcggaacaggagagcgcacgagggagcttccagggggaaacgcctggtatctttatagtcctgtcgggtttcgccacctctgacttgagcgtcgatttttgtgatgctcgtcaggggggcggagcctatggaaaaacgccagcaacgcggcctttttacggttcctggccttttgctggccttttgctcacatgttctttcctgcgttatcccctgattctgtggataaccgtattaccgcctttgagtgagctgataccgctcgccgcagccgaacgaccgagcgcagcgagtcagtgagcgaggaagcggaagagcgcccaatacgcaaaccgcctctccccgcgcgttggccgattcattaatgcagctggcacgacaggtttcccgactggaaagcgggcagtgagcgcaacgcaattagctcactcattaggcaccccaggctttacactttatgcttccggctcgtatgttgtgtggaattgtgagcggataacaatttcacacaggaaacagctatgaccatgattacgcc

The maize ubiquitin promoter is shown as underlined **lowercase** letters. The CDS of humanized LbCas12a is shown as italic capital letters. The nucleus signal peptides coding sequences are shown in italic **lowercase** letters. The NOS terminator is shown as underlined capital letters. The U6 promoter is shown as blue capital letters. The directed repeat of the LbCas12a/FnCas12a crRNA are shown in red capital letters. The two BsaI cut sites for annealed guide sequence oligoes subcloning are shown as underlined blue capital letters. The 7 Ts to terminate the transcription of U6 promoter are shown as magenta capital letters. The enzyme cut sites are all shown in lower case. KpnI is shown in red, EcoRI is shown in purple, XmaI is shown in yellow, SacI is shown in green, BamHI is shown in light blue.

**pA9LbCas12acr-new-version (9LCCnv for short)**

aagcttGCATGCCTGCAGtgcagcgtgacccggtcgtgcccctctctagagataatgagcattgcatgtctaagttataaaaaattaccacatattttttttgtcacacttgtttgaagtgcagtttatctatctttatacatatatttaaactttactctacgaataatataatctatagtactacaataatatcagtgttttagagaatcatataaatgaacagttagacatggtctaaaggacaattgagtattttgacaacaggactctacagttttatctttttagtgtgcatgtgttctcctttttttttgcaaatagcttcacctatataatacttcatccattttattagtacatccatttagggtttagggttaatggtttttatagactaatttttttagtacatctattttattctattttagcctctaaattaagaaaactaaaactctattttagtttttttatttaataatttagatataaaatagaataaaataaagtgactaaaaattaaacaaataccctttaagaaattaaaaaaactaaggaaacatttttcttgtttcgagtagataatgccagcctgttaaacgccgtcgacgagtctaacggacaccaaccagcgaaccagcagcgtcgcgtcgggccaagcgaagcagacggcacggcatctctgtcgctgcctctggacccctctcgagagttccgctccaccgttggacttgctccgctgtcggcatccagaaattgcgtggcggagcggcagacgtgagccggcacggcaggcggcctcctcctcctctcacggcaccggcagctacgggggattcctttcccaccgctccttcgctttcccttcctcgcccgccgtaataaatagacaccccctccacaccctctttccccaacctcgtgttgttcggagcgcacacacacacaaccagatctcccccaaatccacccgtcggcacctccgcttcaaggtacgccgctcgtcctccccccccccccctctctaccttctctagatcggcgttccggtccatggttagggcccggtagttctacttctgttcatgtttgtgttagatccgtgtttgtgttagatccgtgctgctagcgttcgtacacggatgcgacctgtacgtcagacacgttctgattgctaacttgccagtgtttctctttggggaatcctgggatggctctagccgttccgcagacgggatcgatttcatgattttttttgtttcgttgcatagggtttggtttgcccttttcctttatttcaatatatgccgtgcacttgtttgtcgggtcatcttttcatgcttttttttgtcttggttgtgatgatgtggtctggttgggcggtcgttctagatcggagtagaattctgtttcaaactacctggtggatttattaattttggatctgtatgtgtgtgccatacatattcatagttacgaattgaagatgatggatggaaatatcgatctaggataggtatacatgttgatgcgggttttactgatgcatatacagagatgctttttgttcgcttggttgtgatgatgtggtgtggttgggcggtcgttcattcgttctagatcggagtagaatactgtttcaaactacctggtgtatttattaattttggaactgtatgtgtgtgtcatacatcttcatagttacgagtttaagatggatggaaatatcgatctaggataggtatacatgttgatgtgggttttactgatgcatatacatgatggcatatgcagcatctattcatatgctctaaccttgagtacctatctattataataaacaagtatgttttataattattttgatcttgatatacttggatgatggcatatgcagcagctatatgtggatttttttagccctgccttcatacgctatttatttgcttggtactgtttcttttgtcgatgctcaccctgttgtttggtgttacttctgcaggtcgacTCTAGAggatccTAggtaccgccaccATG*gccccaaagaagaagcggaaggtcggtatccacggagtcccagcagccAGCAAGCTGGAGAAGTTTACAAACTGCTACTCCCTGTCTAAGACCCTGAGGTTCAAGGCCATCCCTGTGGGCAAGACCCAGGAGAACATCGACAATAAGCGGCTGCTGGTGGAGGACGAGAAGAGAGCCGAGGATTATAAGGGCGTGAAGAAGCTGCTGGATCGCTACTATCTGTCTTTTATCAACGACGTGCTGCACAGCATCAAGCTGAAGAATCTGAACAATTACATCAGCCTGTTCCGGAAGAAAACCAGAACCGAGAAGGAGAATAAGGAGCTGGAGAACCTGGAGATCAATCTGCGGAAGGAGATCGCCAAGGCCTTCAAGGGCAACGAGGGCTACAAGTCCCTGTTTAAGAAGGATATCATCGAGACAATCCTGCCAGAGTTCCTGGACGATAAGGACGAGATCGCCCTGGTGAACAGCTTCAATGGCTTTACCACAGCCTTCACCGGCTTCTTTGATAACAGAGAGAATATGTTTTCCGAGGAGGCCAAGAGCACATCCATCGCCTTCAGGTGTATCAACGAGAATCTGACCCGCTACATCTCTAATATGGACATCTTCGAGAAGGTGGACGCCATCTTTGATAAGCACGAGGTGCAGGAGATCAAGGAGAAGATCCTGAACAGCGACTATGATGTGGAGGATTTCTTTGAGGGCGAGTTCTTTAACTTTGTGCTGACACAGGAGGGCATCGACGTGTATAACGCCATCATCGGCGGCTTCGTGACCGAGAGCGGCGAGAAGATCAAGGGCCTGAACGAGTACATCAACCTGTATAATCAGAAAACCAAGCAGAAGCTGCCTAAGTTTAAGCCACTGTATAAGCAGGTGCTGAGCGATCGGGAGTCTCTGAGCTTCTACGGCGAGGGCTATACATCCGATGAGGAGGTGCTGGAGGTGTTTAGAAACACCCTGAACAAGAACAGCGAGATCTTCAGCTCCATCAAGAAGCTGGAGAAGCTGTTCAAGAATTTTGACGAGTACTCTAGCGCCGGCATCTTTGTGAAGAACGGCCCCGCCATCAGCACAATCTCCAAGGATATCTTCGGCGAGTGGAACGTGATCCGGGACAAGTGGAATGCCGAGTATGACGATATCCACCTGAAGAAGAAGGCCGTGGTGACCGAGAAGTACGAGGACGATCGGAGAAAGTCCTTCAAGAAGATCGGCTCCTTTTCTCTGGAGCAGCTGCAGGAGTACGCCGACGCCGATCTGTCTGTGGTGGAGAAGCTGAAGGAGATCATCATCCAGAAGGTGGATGAGATCTACAAGGTGTATGGCTCCTCTGAGAAGCTGTTCGACGCCGATTTTGTGCTGGAGAAGAGCCTGAAGAAGAACGACGCCGTGGTGGCCATCATGAAGGACCTGCTGGATTCTGTGAAGAGCTTCGAGAATTACATCAAGGCCTTCTTTGGCGAGGGCAAGGAGACAAACAGGGACGAGTCCTTCTATGGCGATTTTGTGCTGGCCTACGACATCCTGCTGAAGGTGGACCACATCTACGATGCCATCCGCAATTATGTGACCCAGAAGCCCTACTCTAAGGATAAGTTCAAGCTGTATTTTCAGAACCCTCAGTTCATGGGCGGCTGGGACAAGGATAAGGAGACAGACTATCGGGCCACCATCCTGAGATACGGCTCCAAGTACTATCTGGCCATCATGGATAAGAAGTACGCCAAGTGCCTGCAGAAGATCGACAAGGACGATGTGAACGGCAATTACGAGAAGATCAACTATAAGCTGCTGCCCGGCCCTAATAAGATGCTGCCAAAGGTGTTCTTTTCTAAGAAGTGGATGGCCTACTATAACCCCAGCGAGGACATCCAGAAGATCTACAAGAATGGCACATTCAAGAAGGGCGATATGTTTAACCTGAATGACTGTCACAAGCTGATCGACTTCTTTAAGGATAGCATCTCCCGGTATCCAAAGTGGTCCAATGCCTACGATTTCAACTTTTCTGAGACAGAGAAGTATAAGGACATCGCCGGCTTTTACAGAGAGGTGGAGGAGCAGGGCTATAAGGTGAGCTTCGAGTCTGCCAGCAAGAAGGAGGTGGATAAGCTGGTGGAGGAGGGCAAGCTGTATATGTTCCAGATCTATAACAAGGACTTTTCCGATAAGTCTCACGGCACACCCAATCTGCACACCATGTACTTCAAGCTGCTGTTTGACGAGAACAATCACGGACAGATCAGGCTGAGCGGAGGAGCAGAGCTGTTCATGAGGCGCGCCTCCCTGAAGAAGGAGGAGCTGGTGGTGCACCCAGCCAACTCCCCTATCGCCAACAAGAATCCAGATAATCCCAAGAAAACCACAACCCTGTCCTACGACGTGTATAAGGATAAGAGGTTTTCTGAGGACCAGTACGAGCTGCACATCCCAATCGCCATCAATAAGTGCCCCAAGAACATCTTCAAGATCAATACAGAGGTGCGCGTGCTGCTGAAGCACGACGATAACCCCTATGTGATCGGCATCGATAGGGGCGAGCGCAATCTGCTGTATATCGTGGTGGTGGACGGCAAGGGCAACATCGTGGAGCAGTATTCCCTGAACGAGATCATCAACAACTTCAACGGCATCAGGATCAAGACAGATTACCACTCTCTGCTGGACAAGAAGGAGAAGGAGAGGTTCGAGGCCCGCCAGAACTGGACCTCCATCGAGAATATCAAGGAGCTGAAGGCCGGCTATATCTCTCAGGTGGTGCACAAGATCTGCGAGCTGGTGGAGAAGTACGATGCCGTGATCGCCCTGGAGGACCTGAACTCTGGCTTTAAGAATAGCCGCGTGAAGGTGGAGAAGCAGGTGTATCAGAAGTTCGAGAAGATGCTGATCGATAAGCTGAACTACATGGTGGACAAGAAGTCTAATCCTTGTGCAACAGGCGGCGCCCTGAAGGGCTATCAGATCACCAATAAGTTCGAGAGCTTTAAGTCCATGTCTACCCAGAACGGCTTCATCTTTTACATCCCTGCCTGGCTGACATCCAAGATCGATCCATCTACCGGCTTTGTGAACCTGCTGAAAACCAAGTATACCAGCATCGCCGATTCCAAGAAGTTCATCAGCTCCTTTGACAGGATCATGTACGTGCCCGAGGAGGATCTGTTCGAGTTTGCCCTGGACTATAAGAACTTCTCTCGCACAGACGCCGATTACATCAAGAAGTGGAAGCTGTACTCCTACGGCAACCGGATCAGAATCTTCCGGAATCCTAAGAAGAACAACGTGTTCGACTGGGAGGAGGTGTGCCTGACCAGCGCCTATAAGGAGCTGTTCAACAAGTACGGCATCAATTATCAGCAGGGCGATATCAGAGCCCTGCTGTGCGAGCAGTCCGACAAGGCCTTCTACTCTAGCTTTATGGCCCTGATGAGCCTGATGCTGCAGATGCGGAACAGCATCACAGGCCGCACCGACGTGGATTTTCTGATCAGCCCTGTGAAGAACTCCGACGGCATCTTCTACGATAGCCGGAACTATGAGGCCCAGGAGAATGCCATCCTGCCAAAGAACGCCGACGCCAATGGCGCCTATAACATCGCCAGAAAGGTGCTGTGGGCCATCGGCCAGTTCAAGAAGGCCGAGGACGAGAAGCTGGATAAGGTGAAGATCGCCATCTCTAACAAGGAGTGGCTGGAGTACGCCCAGACCAGCGTGAAGCACAAAAGGCCGGCGGCCACGAAAAAGGCCGGCCAGGCAAAAAAGAAAAAGGGATCCtacccatacgatgttccagattacgcttatccctacgacgtgcctgattatgcatacccatatgatgtccccgactatgccTAA*cccggg**GAATTTCCCCGATCGTTCAAACATTTGGCAATAAAGTTTCTTAAGATTGAATCCTGTTGCCGGTCTTGCGATGATTATCATATAATTTCTGTTGAATTACGTTAAGCATGTAATAATTAACATGTAATGCATGACGTTATTTATGAGATGGGTTTTTATGATTAGAGTCCCGCAATTATACATTTAATACGCGATAGAAAACAAAATATAGCGCGCAAACTAGGATAAATTATCGCGCGCGGTGTCATCTATGTTACTAGATCGGGGATCCGACCAAGCCCGTTATTCTGACAGTTCTGGTGCTCAACACATTTATATTTATCAAGGAGCACATTGTTACTCACTGCTAGGAGGGAATCGAACTAGGAATATTGATCAGAGGAACTACGAGAGAGCTGAAGATAACTGCCCTCTAGCTCTCACTGATCTGGGTCGCATAGTGAGATGCAGCCCACGTGAGTTCAGCAACGGTCTAGCGCTGGGCTTTTAGGCCCGCATGATCGGGCTTTTGTCGGGTGGTCGACGTGTTCACGATTGGGGAGAGCAACGCAGCAGTTCCTCTTAGTTTAGTCCCACCTCGCCTGTCCAGCAGAGTTCTGACCGGTTTATAAACTCGCTTGCTGCATCAGACTTG**TAATTTCTACTAAGTGTAGAT**AGAGACCGAGAGAGGGTCTCATTTTTTT**gagctcgaattcactggccgtcgttttacaacgtcgtgactgggaaaaccctggcgttacccaacttaatcgccttgcagcacatccccctttcgccagctggcgtaatagcgaagaggcccgcaccgatcgcccttcccaacagttgcgcagcctgaatggcgaatggcgcctgatgcggtattttctccttacgcatctgtgcggtatttcacaccgcatatggtgcactctcagtacaatctgctctgatgccgcatagttaagccagccccgacacccgccaacacccgctgacgcgccctgacgggcttgtctgctcccggcatccgcttacagacaagctgtgaccgtctccgggagctgcatgtgtcagaggttttcaccgtcatcaccgaaacgcgcgagacgaaagggcctcgtgatacgcctatttttataggttaatgtcatgataataatggtttcttagacgtcaggtggcacttttcggggaaatgtgcgcggaacccctatttgtttatttttctaaatacattcaaatatgtatccgctcatgagacaataaccctgataaatgcttcaataatattgaaaaaggaagagtatgagtattcaacatttccgtgtcgcccttattcccttttttgcggcattttgccttcctgtttttgctcacccagaaacgctggtgaaagtaaaagatgctgaagatcagttgggtgcacgagtgggttacatcgaactggatctcaacagcggtaagatccttgagagttttcgccccgaagaacgttttccaatgatgagcacttttaaagttctgctatgtggcgcggtattatcccgtattgacgccgggcaagagcaactcggtcgccgcatacactattctcagaatgacttggttgagtactcaccagtcacagaaaagcatcttacggatggcatgacagtaagagaattatgcagtgctgccataaccatgagtgataacactgcggccaacttacttctgacaacgatcggaggaccgaaggagctaaccgcttttttgcacaacatgggggatcatgtaactcgccttgatcgttgggaaccggagctgaatgaagccataccaaacgacgagcgtgacaccacgatgcctgtagcaatggcaacaacgttgcgcaaactattaactggcgaactacttactctagcttcccggcaacaattaatagactggatggaggcggataaagttgcaggaccacttctgcgctcggcccttccggctggctggtttattgctgataaatctggagccggtgagcgtggctctcgcggtatcattgcagcactggggccagatggtaagccctcccgtatcgtagttatctacacgacggggagtcaggcaactatggatgaacgaaatagacagatcgctgagataggtgcctcactgattaagcattggtaactgtcagaccaagtttactcatatatactttagattgatttaaaacttcatttttaatttaaaaggatctaggtgaagatcctttttgataatctcatgaccaaaatcccttaacgtgagttttcgttccactgagcgtcagaccccgtagaaaagatcaaaggatcttcttgagatcctttttttctgcgcgtaatctgctgcttgcaaacaaaaaaaccaccgctaccagcggtggtttgtttgccggatcaagagctaccaactctttttccgaaggtaactggcttcagcagagcgcagataccaaatactgttcttctagtgtagccgtagttaggccaccacttcaagaactctgtagcaccgcctacatacctcgctctgctaatcctgttaccagtggctgctgccagtggcgataagtcgtgtcttaccgggttggactcaagacgatagttaccggataaggcgcagcggtcgggctgaacggggggttcgtgcacacagcccagcttggagcgaacgacctacaccgaactgagatacctacagcgtgagctatgagaaagcgccacgcttcccgaagggagaaaggcggacaggtatccggtaagcggcagggtcggaacaggagagcgcacgagggagcttccagggggaaacgcctggtatctttatagtcctgtcgggtttcgccacctctgacttgagcgtcgatttttgtgatgctcgtcaggggggcggagcctatggaaaaacgccagcaacgcggcctttttacggttcctggccttttgctggccttttgctcacatgttctttcctgcgttatcccctgattctgtggataaccgtattaccgcctttgagtgagctgataccgctcgccgcagccgaacgaccgagcgcagcgagtcagtgagcgaggaagcggaagagcgcccaatacgcaaaccgcctctccccgcgcgttggccgattcattaatgcagctggcacgacaggtttcccgactggaaagcgggcagtgagcgcaacgcaattagctcactcattaggcaccccaggctttacactttatgcttccggctcgtatgttgtgtggaattgtgagcggataacaatttcacacaggaaacagctatgaccatgattacgcc

The maize ubiquitin promoter is shown as underlined **lowercase** letters. The CDS of humanized LbCas12a is shown as italic capital letters. The nucleus signal peptides coding sequences are shown in italic **lowercase** letters. The NOS terminator is shown as underlined capital letters. The U6 promoter is shown as blue capital letters. The directed repeat of the LbCas12a/FnCas12a crRNA are shown in red capital letters. The two BsaI cut sites for annealed guide sequence oligoes subcloning are shown as underlined blue capital letters. The 7 Ts to terminate the transcription of U6 promoter are shown as magenta capital letters. The enzyme cut sites are all shown in lower case. KpnI is shown in red, EcoRI is shown in purple, XmaI is shown in yellow, SacI is shown in green, BamHI is shown in light blue.

**pA9FnCas12acr (9FCC for short)**

aagctt**GCATGCCTGCAG**tgcagcgtgacccggtcgtgcccctctctagagataatgagcattgcatgtctaagttataaaaaattaccacatattttttttgtcacacttgtttgaagtgcagtttatctatctttatacatatatttaaactttactctacgaataatataatctatagtactacaataatatcagtgttttagagaatcatataaatgaacagttagacatggtctaaaggacaattgagtattttgacaacaggactctacagttttatctttttagtgtgcatgtgttctcctttttttttgcaaatagcttcacctatataatacttcatccattttattagtacatccatttagggtttagggttaatggtttttatagactaatttttttagtacatctattttattctattttagcctctaaattaagaaaactaaaactctattttagtttttttatttaataatttagatataaaatagaataaaataaagtgactaaaaattaaacaaataccctttaagaaattaaaaaaactaaggaaacatttttcttgtttcgagtagataatgccagcctgttaaacgccgtcgacgagtctaacggacaccaaccagcgaaccagcagcgtcgcgtcgggccaagcgaagcagacggcacggcatctctgtcgctgcctctggacccctctcgagagttccgctccaccgttggacttgctccgctgtcggcatccagaaattgcgtggcggagcggcagacgtgagccsgcacggcaggcggcctcctcctcctctcacggcaccggcagctacgggggattcctttcccaccgctccttcgctttcccttcctcgcccgccgtaataaatagacaccccctccacaccctctttccccaacctcgtgttgttcggagcgcacacacacacaaccagatctcccccaaatccacccgtcggcacctccgcttcaaggtacgccgctcgtcctccccccccccccctctctaccttctctagatcggcgttccggtccatggttagggcccggtagttctacttctgttcatgtttgtgttagatccgtgtttgtgttagatccgtgctgctagcgttcgtacacggatgcgacctgtacgtcagacacgttctgattgctaacttgccagtgtttctctttggggaatcctgggatggctctagccgttccgcagacgggatcgatttcatgattttttttgtttcgttgcatagggtttggtttgcccttttcctttatttcaatatatgccgtgcacttgtttgtcgggtcatcttttcatgcttttttttgtcttggttgtgatgatgtggtctggttgggcggtcgttctagatcggagtagaattctgtttcaaactacctggtggatttattaattttggatctgtatgtgtgtgccatacatattcatagttacgaattgaagatgatggatggaaatatcgatctaggataggtatacatgttgatgcgggttttactgatgcatatacagagatgctttttgttcgcttggttgtgatgatgtggtgtggttgggcggtcgttcattcgttctagatcggagtagaatactgtttcaaactacctggtgtatttattaattttggaactgtatgtgtgtgtcatacatcttcatagttacgagtttaagatggatggaaatatcgatctaggataggtatacatgttgatgtgggttttactgatgcatatacatgatggcatatgcagcatctattcatatgctctaaccttgagtacctatctattataataaacaagtatgttttataattattttgatcttgatatacttggatgatggcatatgcagcagctatatgtggatttttttagccctgccttcatacgctatttatttgcttggtactgtttcttttgtcgatgctcaccctgttgtttggtgttacttctgcaggtcgacTCTAGAggatcctaggtaccgccaccATG*gccccaaagaagaagcggaaggtcggtatccacggagtcccagcagccAGCATTTATCAGGAGTTCGTCAATAAGTACTCTTTGTCGAAGACACTGCGGTTCGAATTGATTCCCCAAGGTAAGACCCTCGAAAACATAAAGGCTAGGGGTCTGATTCTGGATGACGAGAAACGGGCGAAGGATTATAAGAAAGCTAAACAAATTATTGATAAGTATCACCAGTTCTTTATCGAAGAAATCCTTAGCTCAGTTTGTATCTCCGAAGACTTGCTTCAAAACTATTCCGATGTGTACTTCAAACTTAAAAAGAGCGATGATGACAATCTGCAAAAAGACTTTAAGTCAGCGAAGGACACAATTAAAAAACAAATATCCGAGTACATTAAAGATTCTGAGAAGTTCAAGAACCTCTTCAACCAAAACCTGATCGACGCTAAAAAGGGGCAAGAATCAGATCTGATACTCTGGTTGAAGCAATCGAAGGATAATGGAATTGAATTGTTCAAAGCGAACTCCGACATAACGGACATTGACGAGGCTTTGGAGATCATCAAGTCTTTTAAAGGCTGGACTACATACTTCAAAGGATTTCACGAAAATCGTAAGAATGTGTACAGCTCGAATGACATCCCTACTTCCATTATCTACAGGATAGTGGACGACAATCTCCCGAAATTTCTCGAAAACAAAGCGAAGTACGAGTCACTGAAGGACAAAGCACCGGAGGCGATCAACTATGAACAAATCAAGAAGGATCTTGCGGAAGAACTTACGTTCGATATTGATTACAAAACTTCTGAGGTTAACCAGCGTGTGTTTTCACTCGATGAAGTGTTTGAAATAGCTAACTTTAACAACTACCTTAATCAGTCAGGCATAACCAAATTCAATACGATTATTGGAGGTAAGTTTGTCAACGGTGAGAACACTAAGCGTAAGGGAATAAATGAATATATTAACTTGTACTCTCAACAAATAAACGATAAAACACTCAAGAAATACAAGATGTCCGTTCTCTTTAAGCAGATACTCAGCGATACTGAGTCCAAATCGTTCGTGATCGACAAGCTGGAGGACGATAGCGACGTTGTCACTACCATGCAATCCTTCTATGAACAGATTGCAGCCTTTAAAACCGTCGAGGAAAAATCTATCAAGGAGACGTTGTCTCTCCTCTTTGACGATCTGAAAGCCCAGAAGCTGGATCTCAGCAAGATCTACTTTAAAAATGACAAGTCCCTTACGGACCTGTCGCAACAAGTTTTCGACGACTATTCGGTGATCGGTACGGCCGTCCTGGAATACATTACTCAACAGATCGCACCCAAAAATCTGGACAATCCTTCTAAGAAGGAGCAGGAGCTGATCGCCAAAAAGACGGAAAAGGCTAAGTACCTTTCGCTGGAGACAATAAAACTTGCGCTGGAGGAGTTTAATAAACATCGGGACATTGACAAACAGTGCAGGTTCGAAGAAATCCTGGCCAATTTTGCTGCGATTCCGATGATCTTCGACGAGATTGCGCAGAACAAAGATAATTTGGCTCAAATAAGCATAAAGTACCAAAATCAAGGGAAAAAAGACCTCTTGCAAGCAAGCGCGGAGGACGACGTGAAGGCCATTAAGGACCTCCTGGATCAGACGAATAATCTCCTTCATAAACTCAAAATATTTCATATTAGCCAAAGCGAGGACAAAGCGAATATCTTGGACAAAGACGAACATTTCTACCTTGTTTTCGAGGAGTGTTACTTTGAGCTGGCGAACATTGTGCCACTCTATAATAAGATCCGGAACTATATAACCCAAAAGCCCTACTCGGATGAAAAGTTTAAACTCAATTTCGAAAATTCTACGTTGGCTAATGGATGGGACAAGAATAAGGAACCCGATAATACTGCGATTCTTTTCATTAAAGACGATAAGTATTATTTGGGGGTGATGAACAAAAAAAACAACAAAATCTTTGATGACAAAGCGATAAAGGAGAACAAGGGAGAAGGATACAAGAAGATCGTTTACAAACTCCTGCCAGGTGCAAATAAGATGCTCCCCAAAGTCTTCTTCTCGGCCAAGTCAATTAAATTTTACAACCCCAGCGAGGACATCCTTCGGATTAGGAATCATTCTACTCATACAAAAAACGGAAGCCCACAGAAGGGATACGAGAAGTTCGAGTTTAACATTGAGGACTGCCGTAAGTTTATTGATTTCTATAAGCAGTCAATTAGCAAGCACCCAGAATGGAAGGACTTCGGATTCCGGTTTTCTGATACTCAACGTTATAATTCAATAGACGAATTTTATCGTGAAGTGGAGAATCAGGGCTATAAGCTGACCTTCGAGAACATTTCTGAATCGTATATTGATTCGGTTGTCAACCAAGGTAAGTTGTACCTTTTCCAAATATACAACAAGGATTTTTCAGCGTACTCGAAAGGACGCCCTAACCTCCATACCTTGTACTGGAAAGCCTTGTTTGACGAGCGTAACCTTCAGGATGTTGTTTACAAACTCAATGGCGAAGCCGAGTTGTTTTACCGTAAGCAAAGCATACCAAAGAAGATAACTCATCCAGCTAAAGAAGCTATTGCGAACAAGAACAAAGACAATCCCAAGAAAGAATCCGTGTTCGAGTACGACTTGATAAAGGATAAGAGATTCACGGAAGACAAATTTTTCTTCCACTGCCCCATAACGATAAACTTCAAGTCGTCGGGTGCGAATAAATTTAACGACGAAATCAACCTTCTGCTTAAGGAAAAAGCTAATGACGTTCATATTCTTTCAATAGACAGAGGCGAAAGGCACTTGGCGTATTACACGTTGGTCGATGGTAAGGGCAACATCATTAAACAGGATACTTTTAATATTATCGGTAACGACCGGATGAAGACTAATTACCACGATAAACTGGCAGCAATAGAGAAGGACCGTGACAGCGCTAGGAAAGACTGGAAGAAGATTAATAATATCAAAGAGATGAAAGAAGGATACCTGAGCCAAGTGGTCCACGAAATAGCAAAACTCGTCATTGAGTATAATGCGATCGTTGTTTTCGAGGATTTGAACTTCGGATTTAAGCGCGGCAGATTTAAAGTCGAAAAACAGGTTTACCAAAAGCTGGAGAAAATGCTGATTGAAAAACTGAATTATCTTGTCTTCAAAGATAATGAGTTTGACAAAACAGGAGGGGTTCTTAGAGCCTATCAGCTGACGGCTCCTTTTGAGACGTTTAAAAAGATGGGAAAACAAACTGGAATCATTTACTACGTCCCCGCAGGCTTTACTTCGAAGATTTGTCCTGTCACCGGGTTTGTTAACCAGCTGTACCCCAAGTACGAATCTGTTTCTAAGTCGCAAGAGTTCTTTTCAAAGTTTGATAAGATTTGCTACAATCTCGACAAGGGATATTTCGAGTTCTCCTTTGACTATAAGAACTTCGGCGACAAAGCAGCGAAGGGGAAATGGACTATTGCCTCTTTCGGTTCCAGGCTTATAAACTTCCGGAACAGCGATAAAAATCACAATTGGGACACCCGTGAAGTCTACCCCACGAAGGAGCTTGAGAAACTCCTCAAGGATTATAGCATTGAATATGGACATGGTGAATGTATTAAGGCTGCAATCTGTGGAGAGTCAGACAAGAAATTTTTTGCAAAACTGACTTCGGTTCTTAACACGATACTTCAGATGCGCAATTCAAAAACAGGTACGGAGCTGGACTATTTGATTTCCCCTGTCGCAGATGTGAATGGTAACTTTTTCGACTCCAGACAGGCGCCTAAAAACATGCCGCAAGATGCCGACGCGAATGGCGCGTATCACATAGGACTCAAAGGCTTGATGCTCTTGGGGCGGATCAAAAACAACCAGGAGGGGAAAAAGTTGAACCTGGTGATAAAGAATGAAGAGTACTTTGAGTTCGTCCAAAATCGTAATAATAAACGCCCTGCTGCAACAAAGAAAGCAGGTCAGGCTAAAAAAAAGAAAGGGAGCtacccatatgacgtgcccgactatgcatatccgtatgatgttccagattacgcctatccttatgacgtcccagattatgctTAA*cccggg**GAATTTCCCCGATCGTTCAAACATTTGGCAATAAAGTTTCTTAAGATTGAATCCTGTTGCCGGTCTTGCGATGATTATCATATAATTTCTGTTGAATTACGTTAAGCATGTAATAATTAACATGTAATGCATGACGTTATTTATGAGATGGGTTTTTATGATTAGAGTCCCGCAATTATACATTTAATACGCGATAGAAAACAAAATATAGCGCGCAAACTAGGATAAATTATCGCGCGCGGTGTCATCTATGTTACTAGATCGGGGATCCGACCAAGCCCGTTATTCTGACAGTTCTGGTGCTCAACACATTTATATTTATCAAGGAGCACATTGTTACTCACTGCTAGGAGGGAATCGAACTAGGAATATTGATCAGAGGAACTACGAGAGAGCTGAAGATAACTGCCCTCTAGCTCTCACTGATCTGGGTCGCATAGTGAGATGCAGCCCACGTGAGTTCAGCAACGGTCTAGCGCTGGGCTTTTAGGCCCGCATGATCGGGCTTTTGTCGGGTGGTCGACGTGTTCACGATTGGGGAGAGCAACGCAGCAGTTCCTCTTAGTTTAGTCCCACCTCGCCTGTCCAGCAGAGTTCTGACCGGTTTATAAACTCGCTTGCTGCATCAGACTTG**TAATTTCTACTGTTGTAGAT**AGAGACCGAGAGAGGGTCTCATTTTTTT**gagctcgaattcactggccgtcgttttacaacgtcgtgactgggaaaaccctggcgttacccaacttaatcgccttgcagcacatccccctttcgccagctggcgtaatagcgaagaggcccgcaccgatcgcccttcccaacagttgcgcagcctgaatggcgaatggcgcctgatgcggtattttctccttacgcatctgtgcggtatttcacaccgcatatggtgcactctcagtacaatctgctctgatgccgcatagttaagccagccccgacacccgccaacacccgctgacgcgccctgacgggcttgtctgctcccggcatccgcttacagacaagctgtgaccgtctccgggagctgcatgtgtcagaggttttcaccgtcatcaccgaaacgcgcgagacgaaagggcctcgtgatacgcctatttttataggttaatgtcatgataataatggtttcttagacgtcaggtggcacttttcggggaaatgtgcgcggaacccctatttgtttatttttctaaatacattcaaatatgtatccgctcatgagacaataaccctgataaatgcttcaataatattgaaaaaggaagagtatgagtattcaacatttccgtgtcgcccttattcccttttttgcggcattttgccttcctgtttttgctcacccagaaacgctggtgaaagtaaaagatgctgaagatcagttgggtgcacgagtgggttacatcgaactggatctcaacagcggtaagatccttgagagttttcgccccgaagaacgttttccaatgatgagcacttttaaagttctgctatgtggcgcggtattatcccgtattgacgccgggcaagagcaactcggtcgccgcatacactattctcagaatgacttggttgagtactcaccagtcacagaaaagcatcttacggatggcatgacagtaagagaattatgcagtgctgccataaccatgagtgataacactgcggccaacttacttctgacaacgatcggaggaccgaaggagctaaccgcttttttgcacaacatgggggatcatgtaactcgccttgatcgttgggaaccggagctgaatgaagccataccaaacgacgagcgtgacaccacgatgcctgtagcaatggcaacaacgttgcgcaaactattaactggcgaactacttactctagcttcccggcaacaattaatagactggatggaggcggataaagttgcaggaccacttctgcgctcggcccttccggctggctggtttattgctgataaatctggagccggtgagcgtggctctcgcggtatcattgcagcactggggccagatggtaagccctcccgtatcgtagttatctacacgacggggagtcaggcaactatggatgaacgaaatagacagatcgctgagataggtgcctcactgattaagcattggtaactgtcagaccaagtttactcatatatactttagattgatttaaaacttcatttttaatttaaaaggatctaggtgaagatcctttttgataatctcatgaccaaaatcccttaacgtgagttttcgttccactgagcgtcagaccccgtagaaaagatcaaaggatcttcttgagatcctttttttctgcgcgtaatctgctgcttgcaaacaaaaaaaccaccgctaccagcggtggtttgtttgccggatcaagagctaccaactctttttccgaaggtaactggcttcagcagagcgcagataccaaatactgttcttctagtgtagccgtagttaggccaccacttcaagaactctgtagcaccgcctacatacctcgctctgctaatcctgttaccagtggctgctgccagtggcgataagtcgtgtcttaccgggttggactcaagacgatagttaccggataaggcgcagcggtcgggctgaacggggggttcgtgcacacagcccagcttggagcgaacgacctacaccgaactgagatacctacagcgtgagctatgagaaagcgccacgcttcccgaagggagaaaggcggacaggtatccggtaagcggcagggtcggaacaggagagcgcacgagggagcttccagggggaaacgcctggtatctttatagtcctgtcgggtttcgccacctctgacttgagcgtcgatttttgtgatgctcgtcaggggggcggagcctatggaaaaacgccagcaacgcggcctttttacggttcctggccttttgctggccttttgctcacatgttctttcctgcgttatcccctgattctgtggataaccgtattaccgcctttgagtgagctgataccgctcgccgcagccgaacgaccgagcgcagcgagtcagtgagcgaggaagcggaagagcgcccaatacgcaaaccgcctctccccgcgcgttggccgattcattaatgcagctggcacgacaggtttcccgactggaaagcgggcagtgagcgcaacgcaattagctcactcattaggcaccccaggctttacactttatgcttccggctcgtatgttgtgtggaattgtgagcggataacaatttcacacaggaaacagctatgaccatgattacgcc

The maize ubiquitin promoter is shown as underlined **lowercase** letters. The CDS of wheat codon optimized FnCas12a is shown as italic capital letters. The nucleus signal peptides coding sequences are shown in italic **lowercase** letters. The NOS terminator is shown as underlined capital letters. The U6 promoter is shown as blue capital letters. The directed repeat of the LbCas12a/FnCas12a crRNA are shown in red capital letters. The two BsaI cut sites for annealed guide sequence oligoes subcloning are shown as underlined blue capital letters. The 7 Ts to terminate the transcription of U6 promoter are shown as magenta capital letters. The enzyme cut sites are all shown in lower case. KpnI is shown in red, EcoRI is shown in purple, XmaI is shown in yellow, SacI is shown in green, BamHI is shown in light blue.

**pA9LbCas12acr-RVR (9LCCRVR for short)**

aagcttGCATGCCTGCAGtgcagcgtgacccggtcgtgcccctctctagagataatgagcattgcatgtctaagttataaaaaattaccacatattttttttgtcacacttgtttgaagtgcagtttatctatctttatacatatatttaaactttactctacgaataatataatctatagtactacaataatatcagtgttttagagaatcatataaatgaacagttagacatggtctaaaggacaattgagtattttgacaacaggactctacagttttatctttttagtgtgcatgtgttctcctttttttttgcaaatagcttcacctatataatacttcatccattttattagtacatccatttagggtttagggttaatggtttttatagactaatttttttagtacatctattttattctattttagcctctaaattaagaaaactaaaactctattttagtttttttatttaataatttagatataaaatagaataaaataaagtgactaaaaattaaacaaataccctttaagaaattaaaaaaactaaggaaacatttttcttgtttcgagtagataatgccagcctgttaaacgccgtcgacgagtctaacggacaccaaccagcgaaccagcagcgtcgcgtcgggccaagcgaagcagacggcacggcatctctgtcgctgcctctggacccctctcgagagttccgctccaccgttggacttgctccgctgtcggcatccagaaattgcgtggcggagcggcagacgtgagccggcacggcaggcggcctcctcctcctctcacggcaccggcagctacgggggattcctttcccaccgctccttcgctttcccttcctcgcccgccgtaataaatagacaccccctccacaccctctttccccaacctcgtgttgttcggagcgcacacacacacaaccagatctcccccaaatccacccgtcggcacctccgcttcaaggtacgccgctcgtcctccccccccccccctctctaccttctctagatcggcgttccggtccatggttagggcccggtagttctacttctgttcatgtttgtgttagatccgtgtttgtgttagatccgtgctgctagcgttcgtacacggatgcgacctgtacgtcagacacgttctgattgctaacttgccagtgtttctctttggggaatcctgggatggctctagccgttccgcagacgggatcgatttcatgattttttttgtttcgttgcatagggtttggtttgcccttttcctttatttcaatatatgccgtgcacttgtttgtcgggtcatcttttcatgcttttttttgtcttggttgtgatgatgtggtctggttgggcggtcgttctagatcggagtagaattctgtttcaaactacctggtggatttattaattttggatctgtatgtgtgtgccatacatattcatagttacgaattgaagatgatggatggaaatatcgatctaggataggtatacatgttgatgcgggttttactgatgcatatacagagatgctttttgttcgcttggttgtgatgatgtggtgtggttgggcggtcgttcattcgttctagatcggagtagaatactgtttcaaactacctggtgtatttattaattttggaactgtatgtgtgtgtcatacatcttcatagttacgagtttaagatggatggaaatatcgatctaggataggtatacatgttgatgtgggttttactgatgcatatacatgatggcatatgcagcatctattcatatgctctaaccttgagtacctatctattataataaacaagtatgttttataattattttgatcttgatatacttggatgatggcatatgcagcagctatatgtggatttttttagccctgccttcatacgctatttatttgcttggtactgtttcttttgtcgatgctcaccctgttgtttggtgttacttctgcaggtcgacTCTAGAggatccTAggtaccgccaccATG*gccccaaagaagaagcggaaggtcggtatccacggagtcccagcagccAGCAAGCTGGAGAAGTTTACAAACTGCTACTCCCTGTCTAAGACCCTGAGGTTCAAGGCCATCCCTGTGGGCAAGACCCAGGAGAACATCGACAATAAGCGGCTGCTGGTGGAGGACGAGAAGAGAGCCGAGGATTATAAGGGCGTGAAGAAGCTGCTGGATCGCTACTATCTGTCTTTTATCAACGACGTGCTGCACAGCATCAAGCTGAAGAATCTGAACAATTACATCAGCCTGTTCCGGAAGAAAACCAGAACCGAGAAGGAGAATAAGGAGCTGGAGAACCTGGAGATCAATCTGCGGAAGGAGATCGCCAAGGCCTTCAAGGGCAACGAGGGCTACAAGTCCCTGTTTAAGAAGGATATCATCGAGACAATCCTGCCAGAGTTCCTGGACGATAAGGACGAGATCGCCCTGGTGAACAGCTTCAATGGCTTTACCACAGCCTTCACCGGCTTCTTTGATAACAGAGAGAATATGTTTTCCGAGGAGGCCAAGAGCACATCCATCGCCTTCAGGTGTATCAACGAGAATCTGACCCGCTACATCTCTAATATGGACATCTTCGAGAAGGTGGACGCCATCTTTGATAAGCACGAGGTGCAGGAGATCAAGGAGAAGATCCTGAACAGCGACTATGATGTGGAGGATTTCTTTGAGGGCGAGTTCTTTAACTTTGTGCTGACACAGGAGGGCATCGACGTGTATAACGCCATCATCGGCGGCTTCGTGACCGAGAGCGGCGAGAAGATCAAGGGCCTGAACGAGTACATCAACCTGTATAATCAGAAAACCAAGCAGAAGCTGCCTAAGTTTAAGCCACTGTATAAGCAGGTGCTGAGCGATCGGGAGTCTCTGAGCTTCTACGGCGAGGGCTATACATCCGATGAGGAGGTGCTGGAGGTGTTTAGAAACACCCTGAACAAGAACAGCGAGATCTTCAGCTCCATCAAGAAGCTGGAGAAGCTGTTCAAGAATTTTGACGAGTACTCTAGCGCCGGCATCTTTGTGAAGAACGGCCCCGCCATCAGCACAATCTCCAAGGATATCTTCGGCGAGTGGAACGTGATCCGGGACAAGTGGAATGCCGAGTATGACGATATCCACCTGAAGAAGAAGGCCGTGGTGACCGAGAAGTACGAGGACGATCGGAGAAAGTCCTTCAAGAAGATCGGCTCCTTTTCTCTGGAGCAGCTGCAGGAGTACGCCGACGCCGATCTGTCTGTGGTGGAGAAGCTGAAGGAGATCATCATCCAGAAGGTGGATGAGATCTACAAGGTGTATGGCTCCTCTGAGAAGCTGTTCGACGCCGATTTTGTGCTGGAGAAGAGCCTGAAGAAGAACGACGCCGTGGTGGCCATCATGAAGGACCTGCTGGATTCTGTGAAGAGCTTCGAGAATTACATCAAGGCCTTCTTTGGCGAGGGCAAGGAGACAAACAGGGACGAGTCCTTCTATGGCGATTTTGTGCTGGCCTACGACATCCTGCTGAAGGTGGACCACATCTACGATGCCATCCGCAATTATGTGACCCAGAAGCCCTACTCTAAGGATAAGTTCAAGCTGTATTTTCAGAACCCTCAGTTCATGcGCGGCTGGGACAAGGATgtGGAGACAGACagaCGGGCCACCATCCTGAGATACGGCTCCAAGTACTATCTGGCCATCATGGATAAGAAGTACGCCAAGTGCCTGCAGAAGATCGACAAGGACGATGTGAACGGCAATTACGAGAAGATCAACTATAAGCTGCTGCCCGGCCCTAATAAGATGCTGCCAAAGGTGTTCTTTTCTAAGAAGTGGATGGCCTACTATAACCCCAGCGAGGACATCCAGAAGATCTACAAGAATGGCACATTCAAGAAGGGCGATATGTTTAACCTGAATGACTGTCACAAGCTGATCGACTTCTTTAAGGATAGCATCTCCCGGTATCCAAAGTGGTCCAATGCCTACGATTTCAACTTTTCTGAGACAGAGAAGTATAAGGACATCGCCGGCTTTTACAGAGAGGTGGAGGAGCAGGGCTATAAGGTGAGCTTCGAGTCTGCCAGCAAGAAGGAGGTGGATAAGCTGGTGGAGGAGGGCAAGCTGTATATGTTCCAGATCTATAACAAGGACTTTTCCGATAAGTCTCACGGCACACCCAATCTGCACACCATGTACTTCAAGCTGCTGTTTGACGAGAACAATCACGGACAGATCAGGCTGAGCGGAGGAGCAGAGCTGTTCATGAGGCGCGCCTCCCTGAAGAAGGAGGAGCTGGTGGTGCACCCAGCCAACTCCCCTATCGCCAACAAGAATCCAGATAATCCCAAGAAAACCACAACCCTGTCCTACGACGTGTATAAGGATAAGAGGTTTTCTGAGGACCAGTACGAGCTGCACATCCCAATCGCCATCAATAAGTGCCCCAAGAACATCTTCAAGATCAATACAGAGGTGCGCGTGCTGCTGAAGCACGACGATAACCCCTATGTGATCGGCATCGATAGGGGCGAGCGCAATCTGCTGTATATCGTGGTGGTGGACGGCAAGGGCAACATCGTGGAGCAGTATTCCCTGAACGAGATCATCAACAACTTCAACGGCATCAGGATCAAGACAGATTACCACTCTCTGCTGGACAAGAAGGAGAAGGAGAGGTTCGAGGCCCGCCAGAACTGGACCTCCATCGAGAATATCAAGGAGCTGAAGGCCGGCTATATCTCTCAGGTGGTGCACAAGATCTGCGAGCTGGTGGAGAAGTACGATGCCGTGATCGCCCTGGAGGACCTGAACTCTGGCTTTAAGAATAGCCGCGTGAAGGTGGAGAAGCAGGTGTATCAGAAGTTCGAGAAGATGCTGATCGATAAGCTGAACTACATGGTGGACAAGAAGTCTAATCCTTGTGCAACAGGCGGCGCCCTGAAGGGCTATCAGATCACCAATAAGTTCGAGAGCTTTAAGTCCATGTCTACCCAGAACGGCTTCATCTTTTACATCCCTGCCTGGCTGACATCCAAGATCGATCCATCTACCGGCTTTGTGAACCTGCTGAAAACCAAGTATACCAGCATCGCCGATTCCAAGAAGTTCATCAGCTCCTTTGACAGGATCATGTACGTGCCCGAGGAGGATCTGTTCGAGTTTGCCCTGGACTATAAGAACTTCTCTCGCACAGACGCCGATTACATCAAGAAGTGGAAGCTGTACTCCTACGGCAACCGGATCAGAATCTTCCGGAATCCTAAGAAGAACAACGTGTTCGACTGGGAGGAGGTGTGCCTGACCAGCGCCTATAAGGAGCTGTTCAACAAGTACGGCATCAATTATCAGCAGGGCGATATCAGAGCCCTGCTGTGCGAGCAGTCCGACAAGGCCTTCTACTCTAGCTTTATGGCCCTGATGAGCCTGATGCTGCAGATGCGGAACAGCATCACAGGCCGCACCGACGTGGATTTTCTGATCAGCCCTGTGAAGAACTCCGACGGCATCTTCTACGATAGCCGGAACTATGAGGCCCAGGAGAATGCCATCCTGCCAAAGAACGCCGACGCCAATGGCGCCTATAACATCGCCAGAAAGGTGCTGTGGGCCATCGGCCAGTTCAAGAAGGCCGAGGACGAGAAGCTGGATAAGGTGAAGATCGCCATCTCTAACAAGGAGTGGCTGGAGTACGCCCAGACCAGCGTGAAGCACAAAAGGCCGGCGGCCACGAAAAAGGCCGGCCAGGCAAAAAAGAAAAAGGGATCCtacccatacgatgttccagattacgcttatccctacgacgtgcctgattatgcatacccatatgatgtccccgactatgccTAA*cccggg**GAATTTCCCCGATCGTTCAAACATTTGGCAATAAAGTTTCTTAAGATTGAATCCTGTTGCCGGTCTTGCGATGATTATCATATAATTTCTGTTGAATTACGTTAAGCATGTAATAATTAACATGTAATGCATGACGTTATTTATGAGATGGGTTTTTATGATTAGAGTCCCGCAATTATACATTTAATACGCGATAGAAAACAAAATATAGCGCGCAAACTAGGATAAATTATCGCGCGCGGTGTCATCTATGTTACTAGATCGGGGATCCGACCAAGCCCGTTATTCTGACAGTTCTGGTGCTCAACACATTTATATTTATCAAGGAGCACATTGTTACTCACTGCTAGGAGGGAATCGAACTAGGAATATTGATCAGAGGAACTACGAGAGAGCTGAAGATAACTGCCCTCTAGCTCTCACTGATCTGGGTCGCATAGTGAGATGCAGCCCACGTGAGTTCAGCAACGGTCTAGCGCTGGGCTTTTAGGCCCGCATGATCGGGCTTTTGTCGGGTGGTCGACGTGTTCACGATTGGGGAGAGCAACGCAGCAGTTCCTCTTAGTTTAGTCCCACCTCGCCTGTCCAGCAGAGTTCTGACCGGTTTATAAACTCGCTTGCTGCATCAGACTTG**TAATTTCTACTAAGTGTAGAT**AGAGACCGAGAGAGGGTCTCATTTTTTT**gagctcgaattcactggccgtcgttttacaacgtcgtgactgggaaaaccctggcgttacccaacttaatcgccttgcagcacatccccctttcgccagctggcgtaatagcgaagaggcccgcaccgatcgcccttcccaacagttgcgcagcctgaatggcgaatggcgcctgatgcggtattttctccttacgcatctgtgcggtatttcacaccgcatatggtgcactctcagtacaatctgctctgatgccgcatagttaagccagccccgacacccgccaacacccgctgacgcgccctgacgggcttgtctgctcccggcatccgcttacagacaagctgtgaccgtctccgggagctgcatgtgtcagaggttttcaccgtcatcaccgaaacgcgcgagacgaaagggcctcgtgatacgcctatttttataggttaatgtcatgataataatggtttcttagacgtcaggtggcacttttcggggaaatgtgcgcggaacccctatttgtttatttttctaaatacattcaaatatgtatccgctcatgagacaataaccctgataaatgcttcaataatattgaaaaaggaagagtatgagtattcaacatttccgtgtcgcccttattcccttttttgcggcattttgccttcctgtttttgctcacccagaaacgctggtgaaagtaaaagatgctgaagatcagttgggtgcacgagtgggttacatcgaactggatctcaacagcggtaagatccttgagagttttcgccccgaagaacgttttccaatgatgagcacttttaaagttctgctatgtggcgcggtattatcccgtattgacgccgggcaagagcaactcggtcgccgcatacactattctcagaatgacttggttgagtactcaccagtcacagaaaagcatcttacggatggcatgacagtaagagaattatgcagtgctgccataaccatgagtgataacactgcggccaacttacttctgacaacgatcggaggaccgaaggagctaaccgcttttttgcacaacatgggggatcatgtaactcgccttgatcgttgggaaccggagctgaatgaagccataccaaacgacgagcgtgacaccacgatgcctgtagcaatggcaacaacgttgcgcaaactattaactggcgaactacttactctagcttcccggcaacaattaatagactggatggaggcggataaagttgcaggaccacttctgcgctcggcccttccggctggctggtttattgctgataaatctggagccggtgagcgtggctctcgcggtatcattgcagcactggggccagatggtaagccctcccgtatcgtagttatctacacgacggggagtcaggcaactatggatgaacgaaatagacagatcgctgagataggtgcctcactgattaagcattggtaactgtcagaccaagtttactcatatatactttagattgatttaaaacttcatttttaatttaaaaggatctaggtgaagatcctttttgataatctcatgaccaaaatcccttaacgtgagttttcgttccactgagcgtcagaccccgtagaaaagatcaaaggatcttcttgagatcctttttttctgcgcgtaatctgctgcttgcaaacaaaaaaaccaccgctaccagcggtggtttgtttgccggatcaagagctaccaactctttttccgaaggtaactggcttcagcagagcgcagataccaaatactgttcttctagtgtagccgtagttaggccaccacttcaagaactctgtagcaccgcctacatacctcgctctgctaatcctgttaccagtggctgctgccagtggcgataagtcgtgtcttaccgggttggactcaagacgatagttaccggataaggcgcagcggtcgggctgaacggggggttcgtgcacacagcccagcttggagcgaacgacctacaccgaactgagatacctacagcgtgagctatgagaaagcgccacgcttcccgaagggagaaaggcggacaggtatccggtaagcggcagggtcggaacaggagagcgcacgagggagcttccagggggaaacgcctggtatctttatagtcctgtcgggtttcgccacctctgacttgagcgtcgatttttgtgatgctcgtcaggggggcggagcctatggaaaaacgccagcaacgcggcctttttacggttcctggccttttgctggccttttgctcacatgttctttcctgcgttatcccctgattctgtggataaccgtattaccgcctttgagtgagctgataccgctcgccgcagccgaacgaccgagcgcagcgagtcagtgagcgaggaagcggaagagcgcccaatacgcaaaccgcctctccccgcgcgttggccgattcattaatgcagctggcacgacaggtttcccgactggaaagcgggcagtgagcgcaacgcaattagctcactcattaggcaccccaggctttacactttatgcttccggctcgtatgttgtgtggaattgtgagcggataacaatttcacacaggaaacagctatgaccatgattacgcc

The maize ubiquitin promoter is shown as underlined **lowercase** letters. The CDS of humanized LbCas12a-RVR is shown as italic capital letters, except for those mutated nucleotides compared to wild type LbCas12a, which are shown as red italic **lowercase** letter. The nucleus signal peptides coding sequences are shown in italic **lowercase** letters. The NOS terminator is shown as underlined capital letters. The U6 promoter is shown as blue capital letters. The directed repeat of the LbCas12a crRNA are shown in red capital letters. The two BsaI cut sites for annealed guide sequence oligoes subcloning are shown as underlined blue capital letters. The 7 Ts to terminate the transcription of U6 promoter are shown as magenta capital letters. The enzyme cut sites are all shown in lower case. KpnI is shown in red, EcoRI is shown in purple, XmaI is shown in yellow, SacI is shown in green, BamHI is shown in light blue.

**pA9LbCas12acr-enhanced (9lcce)**

aagcttGCATGCCTGCAGtgcagcgtgacccggtcgtgcccctctctagagataatgagcattgcatgtctaagttataaaaaattaccacatattttttttgtcacacttgtttgaagtgcagtttatctatctttatacatatatttaaactttactctacgaataatataatctatagtactacaataatatcagtgttttagagaatcatataaatgaacagttagacatggtctaaaggacaattgagtattttgacaacaggactctacagttttatctttttagtgtgcatgtgttctcctttttttttgcaaatagcttcacctatataatacttcatccattttattagtacatccatttagggtttagggttaatggtttttatagactaatttttttagtacatctattttattctattttagcctctaaattaagaaaactaaaactctattttagtttttttatttaataatttagatataaaatagaataaaataaagtgactaaaaattaaacaaataccctttaagaaattaaaaaaactaaggaaacatttttcttgtttcgagtagataatgccagcctgttaaacgccgtcgacgagtctaacggacaccaaccagcgaaccagcagcgtcgcgtcgggccaagcgaagcagacggcacggcatctctgtcgctgcctctggacccctctcgagagttccgctccaccgttggacttgctccgctgtcggcatccagaaattgcgtggcggagcggcagacgtgagccggcacggcaggcggcctcctcctcctctcacggcaccggcagctacgggggattcctttcccaccgctccttcgctttcccttcctcgcccgccgtaataaatagacaccccctccacaccctctttccccaacctcgtgttgttcggagcgcacacacacacaaccagatctcccccaaatccacccgtcggcacctccgcttcaaggtacgccgctcgtcctccccccccccccctctctaccttctctagatcggcgttccggtccatggttagggcccggtagttctacttctgttcatgtttgtgttagatccgtgtttgtgttagatccgtgctgctagcgttcgtacacggatgcgacctgtacgtcagacacgttctgattgctaacttgccagtgtttctctttggggaatcctgggatggctctagccgttccgcagacgggatcgatttcatgattttttttgtttcgttgcatagggtttggtttgcccttttcctttatttcaatatatgccgtgcacttgtttgtcgggtcatcttttcatgcttttttttgtcttggttgtgatgatgtggtctggttgggcggtcgttctagatcggagtagaattctgtttcaaactacctggtggatttattaattttggatctgtatgtgtgtgccatacatattcatagttacgaattgaagatgatggatggaaatatcgatctaggataggtatacatgttgatgcgggttttactgatgcatatacagagatgctttttgttcgcttggttgtgatgatgtggtgtggttgggcggtcgttcattcgttctagatcggagtagaatactgtttcaaactacctggtgtatttattaattttggaactgtatgtgtgtgtcatacatcttcatagttacgagtttaagatggatggaaatatcgatctaggataggtatacatgttgatgtgggttttactgatgcatatacatgatggcatatgcagcatctattcatatgctctaaccttgagtacctatctattataataaacaagtatgttttataattattttgatcttgatatacttggatgatggcatatgcagcagctatatgtggatttttttagccctgccttcatacgctatttatttgcttggtactgtttcttttgtcgatgctcaccctgttgtttggtgttacttctgcaggtcgacTCTAGAggatccTAggtaccgccaccATG*gccccaaagaagaagcggaaggtcggtatccacggagtcccagcagccAGCAAGCTGGAGAAGTTTACAAACTGCTACTCCCTGTCTAAGACCCTGAGGTTCAAGGCCATCCCTGTGGGCAAGACCCAGGAGAACATCGACAATAAGCGGCTGCTGGTGGAGGACGAGAAGAGAGCCGAGGATTATAAGGGCGTGAAGAAGCTGCTGGATCGCTACTATCTGTCTTTTATCAACGACGTGCTGCACAGCATCAAGCTGAAGAATCTGAACAATTACATCAGCCTGTTCCGGAAGAAAACCAGAACCGAGAAGGAGAATAAGGAGCTGGAGAACCTGGAGATCAATCTGCGGAAGGAGATCGCCAAGGCCTTCAAGGGCAACGAGGGCTACAAGTCCCTGTTTAAGAAGGATATCATCGAGACAATCCTGCCAGAGTTCCTGGACGATAAGGACGAGATCGCCCTGGTGAACAGCTTCAATGGCTTTACCACAGCCTTCACCGGCTTCTTTGATAACAGAGAGAATATGTTTTCCGAGGAGGCCAAGAGCACATCCATCGCCTTCAGGTGTATCAACGAGAATCTGACCCGCTACATCTCTAATATGGACATCTTCGAGAAGGTGGACGCCATCTTTGATAAGCACGAGGTGCAGGAGATCAAGGAGAAGATCCTGAACAGCGACTATGATGTGGAGGATTTCTTTGAGGGCGAGTTCTTTAACTTTGTGCTGACACAGGAGGGCATCGACGTGTATAACGCCATCATCGGCGGCTTCGTGACCGAGAGCGGCGAGAAGATCAAGGGCCTGAACGAGTACATCAACCTGTATAATCAGAAAACCAAGCAGAAGCTGCCTAAGTTTAAGCCACTGTATAAGCAGGTGCTGAGCGATCGGGAGTCTCTGAGCTTCTACGGCGAGGGCTATACATCCGATGAGGAGGTGCTGGAGGTGTTTAGAAACACCCTGAACAAGAACAGCGAGATCTTCAGCTCCATCAAGAAGCTGGAGAAGCTGTTCAAGAATTTTGACGAGTACTCTAGCGCCGGCATCTTTGTGAAGAACGGCCCCGCCATCAGCACAATCTCCAAGGATATCTTCGGCGAGTGGAACGTGATCCGGGACAAGTGGAATGCCGAGTATGACGATATCCACCTGAAGAAGAAGGCCGTGGTGACCGAGAAGTACGAGGACGATCGGAGAAAGTCCTTCAAGAAGATCGGCTCCTTTTCTCTGGAGCAGCTGCAGGAGTACGCCGACGCCGATCTGTCTGTGGTGGAGAAGCTGAAGGAGATCATCATCCAGAAGGTGGATGAGATCTACAAGGTGTATGGCTCCTCTGAGAAGCTGTTCGACGCCGATTTTGTGCTGGAGAAGAGCCTGAAGAAGAACGACGCCGTGGTGGCCATCATGAAGGACCTGCTGGATTCTGTGAAGAGCTTCGAGAATTACATCAAGGCCTTCTTTGGCGAGGGCAAGGAGACAAACAGGGACGAGTCCTTCTATGGCGATTTTGTGCTGGCCTACGACATCCTGCTGAAGGTGGACCACATCTACGATGCCATCCGCAATTATGTGACCCAGAAGCCCTACTCTAAGGATAAGTTCAAGCTGTATTTTCAGAACCCTCAGTTCATGGGCGGCTGGGACAAGGATAAGGAGACAGACTATCGGGCCACCATCCTGAGATACGGCTCCAAGTACTATCTGGCCATCATGGATAAGAAGTACGCCAAGTGCCTGCAGAAGATCGACAAGGACGATGTGAACGGCAATTACGAGAAGATCAACTATAAGCTGCTGCCCGGCCCTAATAAGATGCTGCCAAAGGTGTTCTTTTCTAAGAAGTGGATGGCCTACTATAACCCCAGCGAGGACATCCAGAAGATCTACAAGAATGGCACATTCAAGAAGGGCGATATGTTTAACCTGAATGACTGTCACAAGCTGATCGACTTCTTTAAGGATAGCATCTCCCGGTATCCAAAGTGGTCCAATGCCTACGATTTCAACTTTTCTGAGACAGAGAAGTATAAGGACATCGCCGGCTTTTACAGAGAGGTGGAGGAGCAGGGCTATAAGGTGAGCTTCGAGTCTGCCAGCAAGAAGGAGGTGGATAAGCTGGTGGAGGAGGGCAAGCTGTATATGTTCCAGATCTATAACAAGGACTTTTCCGATAAGTCTCACGGCACACCCAATCTGCACACCATGTACTTCAAGCTGCTGTTTGACGAGAACAATCACGGACAGATCAGGCTGAGCGGAGGAGCAGAGCTGTTCATGAGGCGCGCCTCCCTGAAGAAGGAGGAGCTGGTGGTGCACCCAGCCAACTCCCCTATCGCCAACAAGAATCCAGATAATCCCAAGAAAACCACAACCCTGTCCTACGACGTGTATAAGGATAAGAGGTTTTCTGAGGACCAGTACGAGCTGCACATCCCAATCGCCATCAATAAGTGCCCCAAGAACATCTTCAAGATCAATACAGAGGTGCGCGTGCTGCTGAAGCACGACGATAACCCCTATGTGATCGGCATCGATAGGGGCGAGCGCAATCTGCTGTATATCGTGGTGGTGGACGGCAAGGGCAACATCGTGGAGCAGTATTCCCTGAACGAGATCATCAACAACTTCAACGGCATCAGGATCAAGACAGATTACCACTCTCTGCTGGACAAGAAGGAGAAGGAGAGGTTCGAGGCCCGCCAGAACTGGACCTCCATCGAGAATATCAAGGAGCTGAAGGCCGGCTATATCTCTCAGGTGGTGCACAAGATCTGCGAGCTGGTGGAGAAGTACGATGCCGTGATCGCCCTGGAGGACCTGAACTCTGGCTTTAAGAATAGCCGCGTGAAGGTGGAGAAGCAGGTGTATCAGAAGTTCGAGAAGATGCTGATCGATAAGCTGAACTACATGGTGGACAAGAAGTCTAATCCTTGTGCAACAGGCGGCGCCCTGAAGGGCTATCAGATCACCAATAAGTTCGAGAGCTTTAAGTCCATGTCTACCCAGAACGGCTTCATCTTTTACATCCCTGCCTGGCTGACATCCAAGATCGATCCATCTACCGGCTTTGTGAACCTGCTGAAAACCAAGTATACCAGCATCGCCGATTCCAAGAAGTTCATCAGCTCCTTTGACAGGATCATGTACGTGCCCGAGGAGGATCTGTTCGAGTTTGCCCTGGACTATAAGAACTTCTCTCGCACAGACGCCGATTACATCAAGAAGTGGAAGCTGTACTCCTACGGCAACCGGATCAGAATCTTCCGGAATCCTAAGAAGAACAACGTGTTCGACTGGGAGGAGGTGTGCCTGACCAGCGCCTATAAGGAGCTGTTCAACAAGTACGGCATCAATTATCAGCAGGGCGATATCAGAGCCCTGCTGTGCGAGCAGTCCGACAAGGCCTTCTACTCTAGCTTTATGGCCCTGATGAGCCTGATGCTGCAGATGCGGAACAGCATCACAGGCCGCACCGACGTGGATTTTCTGATCAGCCCTGTGAAGAACTCCGACGGCATCTTCTACGATAGCCGGAACTATGAGGCCCAGGAGAATGCCATCCTGCCAAAGAACGCCGACGCCAATGGCGCCTATAACATCGCCAGAAAGGTGCTGTGGGCCATCGGCCAGTTCAAGAAGGCCGAGGACGAGAAGCTGGATAAGGTGAAGATCGCCATCTCTAACAAGGAGTGGCTGGAGTACGCCCAGACCAGCGTGAAGCACAAAAGGCCGGCGGCCACGAAAAAGGCCGGCCAGGCAAAAAAGAAAAAGGGATCCtacccatacgatgttccagattacgcttatccctacgacgtgcctgattatgcatacccatatgatgtccccgactatgccTAA*cccggg**GAATTTCCCCGATCGTTCAAACATTTGGCAATAAAGTTTCTTAAGATTGAATCCTGTTGCCGGTCTTGCGATGATTATCATATAATTTCTGTTGAATTACGTTAAGCATGTAATAATTAACATGTAATGCATGACGTTATTTATGAGATGGGTTTTTATGATTAGAGTCCCGCAATTATACATTTAATACGCGATAGAAAACAAAATATAGCGCGCAAACTAGGATAAATTATCGCGCGCGGTGTCATCTATGTTACTAGATCGG**actagtCACGTCAGTGTTTGGTTTCCACTAGCACGAGTAGCGCAATCAGAAAATTTTCAATGCATGAAGTACTAAACGAAGTTTATTTAGAAATTTTTTTAAGAAATGAGTGTAATTTTTTGCGACGAATTTAATGACAATAATTAATCGATGATTGCCTACAGTAATGCTACAGTAACCAACCTCTAATCATGCGTCGAATGCGTCATTAGATTCGTCTCGCAAAATAGCACAAGAATTATGAAATTAATTTTACAAACTATTTTTATTTAATACTAATAATTAACTGTCAAAGTTTGTGCTACTCGCAAGAGTAGCGCGAACCAAACACGGCCTGGAGGAGCACGGTAACGGCGTCGACAAACTAACGGCCACCACCCGCCAACGCAAAGGAGACGGATGAGAGTTGACTTCTTGACGGTTCTCCACCCCTCTGTCTCTCTGTCACTGGGCCCTGGGTCCCCCTCTCGAAAGTTCCTCTGGCCGAAATTGCGCGGCGGAGACGAGGCGGGCGGAACCGTCACGGCAGAGGATTCCTTCCCCACCCTGCCTGGCCCGGCCATATATAAACAGCCACCGCCCCTCCCCGTTCCCCatcgcgtctcgtctcgtgttgttcccagaacacaaccaaaatccaaatcctcctcctcctcccgagcctcgtcgatccctcacccgcttcaagGTACGGCGATCCTCCTCTCCCTTCTCCCCTCGATCGATTATGCGTGTTCCGTTTCCGTTTCCGATCGAGCGAATCGATGGTTAGGACCCATGGGGGACCCATGGGGTGTCGTGTGGTGGTCTGGTTTGATCCGCGATATTTCTCCGTTCGTAGTGTAGATCTGATCGAATCCCTGGTGAAATCGTTGATCGTGCTATTCGTGTGAGGGTTCTTAGGTTTGGAGTTGTGGAGGTAGTTCTGATCGGTTTGTAGGTGAGATTTTCCCCATGATTTTGCTTGGCTCGTTTGTCTTGGTTAGATTAGATCTGCCCGCATTTTGTTCGATATTTCTGATGCAGATATGATGAATAATTTCGTCCTTGTATCCCGCGTCCGTATGTGTATTAAGTTTGCAGGTCCTAGTTAGGTTTTTCCTACTGATTTGTCTTATCCATTCTGTTTAGCTTGCAAGGTTTGGTAATGGTCCGGCATGTTTGTCTCTATAGATTAGAGTAGAATAAGATTATCTCAACAAGCTGTTGGCTTATCAATTTTGGATCTGCATGTGTTTCGCATCTATATCTTTGCAATTAAGATGGTAGATGGACATATGCTCCTGTTGAGTTGATGTTGTACCTTTTACCTGAGGTCTGAGGAACATGCATCCTCCTGCTACTTTGTGCTTATACAGATCATCAAGATTATGTAGCTAATATTCGATCAGTTTCTAGTATCTACATGGTAAACTTGCATGCACTTGCTACTTATTTTTGATATACTTGGATGATAACATATGCTGCTGGTTGATTCCTACCTACATGATGAACATTTTACAGGCCATTAGTGTCTGTCTGTATGTGTTGTTCCTGTTTGCTTCAGTCTATTTCTGTTTCATTCCTAGTTTATTGGTTCTCTGCTAGATACTTACCCTGCTGGGCTTAGTTATCATCTTATCTCGAATGCATTTTCATGTTTATAGATGAATATACACTCAGATAGGTGTAGATGTATGCTACTGTTTCTCTACGTTGCTGTAGGTTTTACCTGTGGCAACTGCATACTCCTGTTGCTTCGCTAGATATGTATGTGCTTATATAGATTAAGATATGTGTGATGGTTCTTTAGTATATCTGATGATCATGTATGCTCTTTTAACTTCTTGCTACACTTGGTAACATGCTGTGATGCTGTTTGTTGATTCTGTAGCACTACCAATGATGACCTTATCTCTCTTTGTATATGATGTTTCTGTTTGTTTGAGGCTTGTGTTACTGCTAGTTACTTACCCTGTTGCCTGGCTAATCTTCTGCAGaaattactgatgagtccgtgaggacgaaacgagtaagctcgtcTAATTTCTACTAAGTGTAGAT**AGAGACCGAGAGAGGGTCTCA**ggccggcatggtcccagcctcctcgctggcgccggctgggcaacatgcttcggcatggcgaatgggac**TCGAGTTTCTCCATAATAATGTGTGAGTAGTTCCCAGATAAGGGAATTAGGGTTCCTATAGGGTTTCGCTCATGTGTTGAGCATATAAGAAACCCTTAGTATGTATTTGTATTTGTAAAATACTTCTATCAATAAAATTTCTAATTCCTAAAACCAAAATCCAGTACTAAAATCCAGATCCCCCGAATTA**gagctcgaattcactggccgtcgttttacaacgtcgtgactgggaaaaccctggcgttacccaacttaatcgccttgcagcacatccccctttcgccagctggcgtaatagcgaagaggcccgcaccgatcgcccttcccaacagttgcgcagcctgaatggcgaatggcgcctgatgcggtattttctccttacgcatctgtgcggtatttcacaccgcatatggtgcactctcagtacaatctgctctgatgccgcatagttaagccagccccgacacccgccaacacccgctgacgcgccctgacgggcttgtctgctcccggcatccgcttacagacaagctgtgaccgtctccgggagctgcatgtgtcagaggttttcaccgtcatcaccgaaacgcgcgagacgaaagggcctcgtgatacgcctatttttataggttaatgtcatgataataatggtttcttagacgtcaggtggcacttttcggggaaatgtgcgcggaacccctatttgtttatttttctaaatacattcaaatatgtatccgctcatgagacaataaccctgataaatgcttcaataatattgaaaaaggaagagtatgagtattcaacatttccgtgtcgcccttattcccttttttgcggcattttgccttcctgtttttgctcacccagaaacgctggtgaaagtaaaagatgctgaagatcagttgggtgcacgagtgggttacatcgaactggatctcaacagcggtaagatccttgagagttttcgccccgaagaacgttttccaatgatgagcacttttaaagttctgctatgtggcgcggtattatcccgtattgacgccgggcaagagcaactcggtcgccgcatacactattctcagaatgacttggttgagtactcaccagtcacagaaaagcatcttacggatggcatgacagtaagagaattatgcagtgctgccataaccatgagtgataacactgcggccaacttacttctgacaacgatcggaggaccgaaggagctaaccgcttttttgcacaacatgggggatcatgtaactcgccttgatcgttgggaaccggagctgaatgaagccataccaaacgacgagcgtgacaccacgatgcctgtagcaatggcaacaacgttgcgcaaactattaactggcgaactacttactctagcttcccggcaacaattaatagactggatggaggcggataaagttgcaggaccacttctgcgctcggcccttccggctggctggtttattgctgataaatctggagccggtgagcgtggctctcgcggtatcattgcagcactggggccagatggtaagccctcccgtatcgtagttatctacacgacggggagtcaggcaactatggatgaacgaaatagacagatcgctgagataggtgcctcactgattaagcattggtaactgtcagaccaagtttactcatatatactttagattgatttaaaacttcatttttaatttaaaaggatctaggtgaagatcctttttgataatctcatgaccaaaatcccttaacgtgagttttcgttccactgagcgtcagaccccgtagaaaagatcaaaggatcttcttgagatcctttttttctgcgcgtaatctgctgcttgcaaacaaaaaaaccaccgctaccagcggtggtttgtttgccggatcaagagctaccaactctttttccgaaggtaactggcttcagcagagcgcagataccaaatactgttcttctagtgtagccgtagttaggccaccacttcaagaactctgtagcaccgcctacatacctcgctctgctaatcctgttaccagtggctgctgccagtggcgataagtcgtgtcttaccgggttggactcaagacgatagttaccggataaggcgcagcggtcgggctgaacggggggttcgtgcacacagcccagcttggagcgaacgacctacaccgaactgagatacctacagcgtgagctatgagaaagcgccacgcttcccgaagggagaaaggcggacaggtatccggtaagcggcagggtcggaacaggagagcgcacgagggagcttccagggggaaacgcctggtatctttatagtcctgtcgggtttcgccacctctgacttgagcgtcgatttttgtgatgctcgtcaggggggcggagcctatggaaaaacgccagcaacgcggcctttttacggttcctggccttttgctggccttttgctcacatgttctttcctgcgttatcccctgattctgtggataaccgtattaccgcctttgagtgagctgataccgctcgccgcagccgaacgaccgagcgcagcgagtcagtgagcgaggaagcggaagagcgcccaatacgcaaaccgcctctccccgcgcgttggccgattcattaatgcagctggcacgacaggtttcccgactggaaagcgggcagtgagcgcaacgcaattagctcactcattaggcaccccaggctttacactttatgcttccggctcgtatgttgtgtggaattgtgagcggataacaatttcacacaggaaacagctatgaccatgattacgcc

The maize ubiquitin promoter is shown as underlined **lowercase** letters. The CDS of humanized LbCas12a is shown as italic capital letters. The nucleus signal peptides coding sequences are shown in italic **lowercase** letters. The NOS terminator is shown as underlined capital letters. The Switch grass ubiquitin promoter and the intron are shown as blue capital letters when the 5’ UTR is shown in **lowercase** blue letters. The hammerhead ribozyme and the HDV ribozyme are shown as brown **lowercase** letters. The directed repeat of the LbCas12a crRNA are shown in red capital letters. The two BsaI cut sites for annealed guide sequence oligoes subcloning are shown as underlined blue capital letters. The 35S terminator is shown as magenta capital letters. The enzyme cut sites are all shown in lower case. KpnI is shown in red, EcoRI is shown in purple, XmaI is shown in yellow, SpeI is shown in orange. SacI is shown in green, BamHI is shown in light blue.

**pA9LbCas12acr-enhanced2 (9lcce2)**

aagcttGCATGCCTGCAGtgcagcgtgacccggtcgtgcccctctctagagataatgagcattgcatgtctaagttataaaaaattaccacatattttttttgtcacacttgtttgaagtgcagtttatctatctttatacatatatttaaactttactctacgaataatataatctatagtactacaataatatcagtgttttagagaatcatataaatgaacagttagacatggtctaaaggacaattgagtattttgacaacaggactctacagttttatctttttagtgtgcatgtgttctcctttttttttgcaaatagcttcacctatataatacttcatccattttattagtacatccatttagggtttagggttaatggtttttatagactaatttttttagtacatctattttattctattttagcctctaaattaagaaaactaaaactctattttagtttttttatttaataatttagatataaaatagaataaaataaagtgactaaaaattaaacaaataccctttaagaaattaaaaaaactaaggaaacatttttcttgtttcgagtagataatgccagcctgttaaacgccgtcgacgagtctaacggacaccaaccagcgaaccagcagcgtcgcgtcgggccaagcgaagcagacggcacggcatctctgtcgctgcctctggacccctctcgagagttccgctccaccgttggacttgctccgctgtcggcatccagaaattgcgtggcggagcggcagacgtgagccggcacggcaggcggcctcctcctcctctcacggcaccggcagctacgggggattcctttcccaccgctccttcgctttcccttcctcgcccgccgtaataaatagacaccccctccacaccctctttccccaacctcgtgttgttcggagcgcacacacacacaaccagatctcccccaaatccacccgtcggcacctccgcttcaaggtacgccgctcgtcctccccccccccccctctctaccttctctagatcggcgttccggtccatggttagggcccggtagttctacttctgttcatgtttgtgttagatccgtgtttgtgttagatccgtgctgctagcgttcgtacacggatgcgacctgtacgtcagacacgttctgattgctaacttgccagtgtttctctttggggaatcctgggatggctctagccgttccgcagacgggatcgatttcatgattttttttgtttcgttgcatagggtttggtttgcccttttcctttatttcaatatatgccgtgcacttgtttgtcgggtcatcttttcatgcttttttttgtcttggttgtgatgatgtggtctggttgggcggtcgttctagatcggagtagaattctgtttcaaactacctggtggatttattaattttggatctgtatgtgtgtgccatacatattcatagttacgaattgaagatgatggatggaaatatcgatctaggataggtatacatgttgatgcgggttttactgatgcatatacagagatgctttttgttcgcttggttgtgatgatgtggtgtggttgggcggtcgttcattcgttctagatcggagtagaatactgtttcaaactacctggtgtatttattaattttggaactgtatgtgtgtgtcatacatcttcatagttacgagtttaagatggatggaaatatcgatctaggataggtatacatgttgatgtgggttttactgatgcatatacatgatggcatatgcagcatctattcatatgctctaaccttgagtacctatctattataataaacaagtatgttttataattattttgatcttgatatacttggatgatggcatatgcagcagctatatgtggatttttttagccctgccttcatacgctatttatttgcttggtactgtttcttttgtcgatgctcaccctgttgtttggtgttacttctgcaggtcgacTCTAGAggatccTAggtaccgccaccATG*gccccaaagaagaagcggaaggtcggtatccacggagtcccagcagccAGCAAGCTGGAGAAGTTTACAAACTGCTACTCCCTGTCTAAGACCCTGAGGTTCAAGGCCATCCCTGTGGGCAAGACCCAGGAGAACATCGACAATAAGCGGCTGCTGGTGGAGGACGAGAAGAGAGCCGAGGATTATAAGGGCGTGAAGAAGCTGCTGGATCGCTACTATCTGTCTTTTATCAACGACGTGCTGCACAGCATCAAGCTGAAGAATCTGAACAATTACATCAGCCTGTTCCGGAAGAAAACCAGAACCGAGAAGGAGAATAAGGAGCTGGAGAACCTGGAGATCAATCTGCGGAAGGAGATCGCCAAGGCCTTCAAGGGCAACGAGGGCTACAAGTCCCTGTTTAAGAAGGATATCATCGAGACAATCCTGCCAGAGTTCCTGGACGATAAGGACGAGATCGCCCTGGTGAACAGCTTCAATGGCTTTACCACAGCCTTCACCGGCTTCTTTGATAACAGAGAGAATATGTTTTCCGAGGAGGCCAAGAGCACATCCATCGCCTTCAGGTGTATCAACGAGAATCTGACCCGCTACATCTCTAATATGGACATCTTCGAGAAGGTGGACGCCATCTTTGATAAGCACGAGGTGCAGGAGATCAAGGAGAAGATCCTGAACAGCGACTATGATGTGGAGGATTTCTTTGAGGGCGAGTTCTTTAACTTTGTGCTGACACAGGAGGGCATCGACGTGTATAACGCCATCATCGGCGGCTTCGTGACCGAGAGCGGCGAGAAGATCAAGGGCCTGAACGAGTACATCAACCTGTATAATCAGAAAACCAAGCAGAAGCTGCCTAAGTTTAAGCCACTGTATAAGCAGGTGCTGAGCGATCGGGAGTCTCTGAGCTTCTACGGCGAGGGCTATACATCCGATGAGGAGGTGCTGGAGGTGTTTAGAAACACCCTGAACAAGAACAGCGAGATCTTCAGCTCCATCAAGAAGCTGGAGAAGCTGTTCAAGAATTTTGACGAGTACTCTAGCGCCGGCATCTTTGTGAAGAACGGCCCCGCCATCAGCACAATCTCCAAGGATATCTTCGGCGAGTGGAACGTGATCCGGGACAAGTGGAATGCCGAGTATGACGATATCCACCTGAAGAAGAAGGCCGTGGTGACCGAGAAGTACGAGGACGATCGGAGAAAGTCCTTCAAGAAGATCGGCTCCTTTTCTCTGGAGCAGCTGCAGGAGTACGCCGACGCCGATCTGTCTGTGGTGGAGAAGCTGAAGGAGATCATCATCCAGAAGGTGGATGAGATCTACAAGGTGTATGGCTCCTCTGAGAAGCTGTTCGACGCCGATTTTGTGCTGGAGAAGAGCCTGAAGAAGAACGACGCCGTGGTGGCCATCATGAAGGACCTGCTGGATTCTGTGAAGAGCTTCGAGAATTACATCAAGGCCTTCTTTGGCGAGGGCAAGGAGACAAACAGGGACGAGTCCTTCTATGGCGATTTTGTGCTGGCCTACGACATCCTGCTGAAGGTGGACCACATCTACGATGCCATCCGCAATTATGTGACCCAGAAGCCCTACTCTAAGGATAAGTTCAAGCTGTATTTTCAGAACCCTCAGTTCATGGGCGGCTGGGACAAGGATAAGGAGACAGACTATCGGGCCACCATCCTGAGATACGGCTCCAAGTACTATCTGGCCATCATGGATAAGAAGTACGCCAAGTGCCTGCAGAAGATCGACAAGGACGATGTGAACGGCAATTACGAGAAGATCAACTATAAGCTGCTGCCCGGCCCTAATAAGATGCTGCCAAAGGTGTTCTTTTCTAAGAAGTGGATGGCCTACTATAACCCCAGCGAGGACATCCAGAAGATCTACAAGAATGGCACATTCAAGAAGGGCGATATGTTTAACCTGAATGACTGTCACAAGCTGATCGACTTCTTTAAGGATAGCATCTCCCGGTATCCAAAGTGGTCCAATGCCTACGATTTCAACTTTTCTGAGACAGAGAAGTATAAGGACATCGCCGGCTTTTACAGAGAGGTGGAGGAGCAGGGCTATAAGGTGAGCTTCGAGTCTGCCAGCAAGAAGGAGGTGGATAAGCTGGTGGAGGAGGGCAAGCTGTATATGTTCCAGATCTATAACAAGGACTTTTCCGATAAGTCTCACGGCACACCCAATCTGCACACCATGTACTTCAAGCTGCTGTTTGACGAGAACAATCACGGACAGATCAGGCTGAGCGGAGGAGCAGAGCTGTTCATGAGGCGCGCCTCCCTGAAGAAGGAGGAGCTGGTGGTGCACCCAGCCAACTCCCCTATCGCCAACAAGAATCCAGATAATCCCAAGAAAACCACAACCCTGTCCTACGACGTGTATAAGGATAAGAGGTTTTCTGAGGACCAGTACGAGCTGCACATCCCAATCGCCATCAATAAGTGCCCCAAGAACATCTTCAAGATCAATACAGAGGTGCGCGTGCTGCTGAAGCACGACGATAACCCCTATGTGATCGGCATCGATAGGGGCGAGCGCAATCTGCTGTATATCGTGGTGGTGGACGGCAAGGGCAACATCGTGGAGCAGTATTCCCTGAACGAGATCATCAACAACTTCAACGGCATCAGGATCAAGACAGATTACCACTCTCTGCTGGACAAGAAGGAGAAGGAGAGGTTCGAGGCCCGCCAGAACTGGACCTCCATCGAGAATATCAAGGAGCTGAAGGCCGGCTATATCTCTCAGGTGGTGCACAAGATCTGCGAGCTGGTGGAGAAGTACGATGCCGTGATCGCCCTGGAGGACCTGAACTCTGGCTTTAAGAATAGCCGCGTGAAGGTGGAGAAGCAGGTGTATCAGAAGTTCGAGAAGATGCTGATCGATAAGCTGAACTACATGGTGGACAAGAAGTCTAATCCTTGTGCAACAGGCGGCGCCCTGAAGGGCTATCAGATCACCAATAAGTTCGAGAGCTTTAAGTCCATGTCTACCCAGAACGGCTTCATCTTTTACATCCCTGCCTGGCTGACATCCAAGATCGATCCATCTACCGGCTTTGTGAACCTGCTGAAAACCAAGTATACCAGCATCGCCGATTCCAAGAAGTTCATCAGCTCCTTTGACAGGATCATGTACGTGCCCGAGGAGGATCTGTTCGAGTTTGCCCTGGACTATAAGAACTTCTCTCGCACAGACGCCGATTACATCAAGAAGTGGAAGCTGTACTCCTACGGCAACCGGATCAGAATCTTCCGGAATCCTAAGAAGAACAACGTGTTCGACTGGGAGGAGGTGTGCCTGACCAGCGCCTATAAGGAGCTGTTCAACAAGTACGGCATCAATTATCAGCAGGGCGATATCAGAGCCCTGCTGTGCGAGCAGTCCGACAAGGCCTTCTACTCTAGCTTTATGGCCCTGATGAGCCTGATGCTGCAGATGCGGAACAGCATCACAGGCCGCACCGACGTGGATTTTCTGATCAGCCCTGTGAAGAACTCCGACGGCATCTTCTACGATAGCCGGAACTATGAGGCCCAGGAGAATGCCATCCTGCCAAAGAACGCCGACGCCAATGGCGCCTATAACATCGCCAGAAAGGTGCTGTGGGCCATCGGCCAGTTCAAGAAGGCCGAGGACGAGAAGCTGGATAAGGTGAAGATCGCCATCTCTAACAAGGAGTGGCTGGAGTACGCCCAGACCAGCGTGAAGCACAAAAGGCCGGCGGCCACGAAAAAGGCCGGCCAGGCAAAAAAGAAAAAGGGATCCtacccatacgatgttccagattacgcttatccctacgacgtgcctgattatgcatacccatatgatgtccccgactatgccTAA*cccggg**GAATTTCCCCGATCGTTCAAACATTTGGCAATAAAGTTTCTTAAGATTGAATCCTGTTGCCGGTCTTGCGATGATTATCATATAATTTCTGTTGAATTACGTTAAGCATGTAATAATTAACATGTAATGCATGACGTTATTTATGAGATGGGTTTTTATGATTAGAGTCCCGCAATTATACATTTAATACGCGATAGAAAACAAAATATAGCGCGCAAACTAGGATAAATTATCGCGCGCGGTGTCATCTATGTTACTAGATCGG**actagtCACGTCAGTGTTTGGTTTCCACTAGCACGAGTAGCGCAATCAGAAAATTTTCAATGCATGAAGTACTAAACGAAGTTTATTTAGAAATTTTTTTAAGAAATGAGTGTAATTTTTTGCGACGAATTTAATGACAATAATTAATCGATGATTGCCTACAGTAATGCTACAGTAACCAACCTCTAATCATGCGTCGAATGCGTCATTAGATTCGTCTCGCAAAATAGCACAAGAATTATGAAATTAATTTTACAAACTATTTTTATTTAATACTAATAATTAACTGTCAAAGTTTGTGCTACTCGCAAGAGTAGCGCGAACCAAACACGGCCTGGAGGAGCACGGTAACGGCGTCGACAAACTAACGGCCACCACCCGCCAACGCAAAGGAGACGGATGAGAGTTGACTTCTTGACGGTTCTCCACCCCTCTGTCTCTCTGTCACTGGGCCCTGGGTCCCCCTCTCGAAAGTTCCTCTGGCCGAAATTGCGCGGCGGAGACGAGGCGGGCGGAACCGTCACGGCAGAGGATTCCTTCCCCACCCTGCCTGGCCCGGCCATATATAAACAGCCACCGCCCCTCCCCGTTCCCCatcgcgtctcgtctcgtgttgttcccagaacacaaccaaaatccaaatcctcctcctcctcccgagcctcgtcgatccctcacccgcttcaagGTACGGCGATCCTCCTCTCCCTTCTCCCCTCGATCGATTATGCGTGTTCCGTTTCCGTTTCCGATCGAGCGAATCGATGGTTAGGACCCATGGGGGACCCATGGGGTGTCGTGTGGTGGTCTGGTTTGATCCGCGATATTTCTCCGTTCGTAGTGTAGATCTGATCGAATCCCTGGTGAAATCGTTGATCGTGCTATTCGTGTGAGGGTTCTTAGGTTTGGAGTTGTGGAGGTAGTTCTGATCGGTTTGTAGGTGAGATTTTCCCCATGATTTTGCTTGGCTCGTTTGTCTTGGTTAGATTAGATCTGCCCGCATTTTGTTCGATATTTCTGATGCAGATATGATGAATAATTTCGTCCTTGTATCCCGCGTCCGTATGTGTATTAAGTTTGCAGGTCCTAGTTAGGTTTTTCCTACTGATTTGTCTTATCCATTCTGTTTAGCTTGCAAGGTTTGGTAATGGTCCGGCATGTTTGTCTCTATAGATTAGAGTAGAATAAGATTATCTCAACAAGCTGTTGGCTTATCAATTTTGGATCTGCATGTGTTTCGCATCTATATCTTTGCAATTAAGATGGTAGATGGACATATGCTCCTGTTGAGTTGATGTTGTACCTTTTACCTGAGGTCTGAGGAACATGCATCCTCCTGCTACTTTGTGCTTATACAGATCATCAAGATTATGTAGCTAATATTCGATCAGTTTCTAGTATCTACATGGTAAACTTGCATGCACTTGCTACTTATTTTTGATATACTTGGATGATAACATATGCTGCTGGTTGATTCCTACCTACATGATGAACATTTTACAGGCCATTAGTGTCTGTCTGTATGTGTTGTTCCTGTTTGCTTCAGTCTATTTCTGTTTCATTCCTAGTTTATTGGTTCTCTGCTAGATACTTACCCTGCTGGGCTTAGTTATCATCTTATCTCGAATGCATTTTCATGTTTATAGATGAATATACACTCAGATAGGTGTAGATGTATGCTACTGTTTCTCTACGTTGCTGTAGGTTTTACCTGTGGCAACTGCATACTCCTGTTGCTTCGCTAGATATGTATGTGCTTATATAGATTAAGATATGTGTGATGGTTCTTTAGTATATCTGATGATCATGTATGCTCTTTTAACTTCTTGCTACACTTGGTAACATGCTGTGATGCTGTTTGTTGATTCTGTAGCACTACCAATGATGACCTTATCTCTCTTTGTATATGATGTTTCTGTTTGTTTGAGGCTTGTGTTACTGCTAGTTACTTACCCTGTTGCCTGGCTAATCTTTAATTTCTACTAAGTGTAGAT**AGAGACCGAGAGAGGGTCTCA**TAATTTCTACTAAGTGTAGAT**CTCGAGTTTCTCCATAATAATGTGTGAGTAGTTCCCAGATAAGGGAATTAGGGTTCCTATAGGGTTTCGCTCATGTGTTGAGCATATAAGAAACCCTTAGTATGTATTTGTATTTGTAAAATACTTCTATCAATAAAATTTCTAATTCCTAAAACCAAAATCCAGTACTAAAATCCAGATCCCCCGAATTA**gagctcgaattcactggccgtcgttttacaacgtcgtgactgggaaaaccctggcgttacccaacttaatcgccttgcagcacatccccctttcgccagctggcgtaatagcgaagaggcccgcaccgatcgcccttcccaacagttgcgcagcctgaatggcgaatggcgcctgatgcggtattttctccttacgcatctgtgcggtatttcacaccgcatatggtgcactctcagtacaatctgctctgatgccgcatagttaagccagccccgacacccgccaacacccgctgacgcgccctgacgggcttgtctgctcccggcatccgcttacagacaagctgtgaccgtctccgggagctgcatgtgtcagaggttttcaccgtcatcaccgaaacgcgcgagacgaaagggcctcgtgatacgcctatttttataggttaatgtcatgataataatggtttcttagacgtcaggtggcacttttcggggaaatgtgcgcggaacccctatttgtttatttttctaaatacattcaaatatgtatccgctcatgagacaataaccctgataaatgcttcaataatattgaaaaaggaagagtatgagtattcaacatttccgtgtcgcccttattcccttttttgcggcattttgccttcctgtttttgctcacccagaaacgctggtgaaagtaaaagatgctgaagatcagttgggtgcacgagtgggttacatcgaactggatctcaacagcggtaagatccttgagagttttcgccccgaagaacgttttccaatgatgagcacttttaaagttctgctatgtggcgcggtattatcccgtattgacgccgggcaagagcaactcggtcgccgcatacactattctcagaatgacttggttgagtactcaccagtcacagaaaagcatcttacggatggcatgacagtaagagaattatgcagtgctgccataaccatgagtgataacactgcggccaacttacttctgacaacgatcggaggaccgaaggagctaaccgcttttttgcacaacatgggggatcatgtaactcgccttgatcgttgggaaccggagctgaatgaagccataccaaacgacgagcgtgacaccacgatgcctgtagcaatggcaacaacgttgcgcaaactattaactggcgaactacttactctagcttcccggcaacaattaatagactggatggaggcggataaagttgcaggaccacttctgcgctcggcccttccggctggctggtttattgctgataaatctggagccggtgagcgtggctctcgcggtatcattgcagcactggggccagatggtaagccctcccgtatcgtagttatctacacgacggggagtcaggcaactatggatgaacgaaatagacagatcgctgagataggtgcctcactgattaagcattggtaactgtcagaccaagtttactcatatatactttagattgatttaaaacttcatttttaatttaaaaggatctaggtgaagatcctttttgataatctcatgaccaaaatcccttaacgtgagttttcgttccactgagcgtcagaccccgtagaaaagatcaaaggatcttcttgagatcctttttttctgcgcgtaatctgctgcttgcaaacaaaaaaaccaccgctaccagcggtggtttgtttgccggatcaagagctaccaactctttttccgaaggtaactggcttcagcagagcgcagataccaaatactgttcttctagtgtagccgtagttaggccaccacttcaagaactctgtagcaccgcctacatacctcgctctgctaatcctgttaccagtggctgctgccagtggcgataagtcgtgtcttaccgggttggactcaagacgatagttaccggataaggcgcagcggtcgggctgaacggggggttcgtgcacacagcccagcttggagcgaacgacctacaccgaactgagatacctacagcgtgagctatgagaaagcgccacgcttcccgaagggagaaaggcggacaggtatccggtaagcggcagggtcggaacaggagagcgcacgagggagcttccagggggaaacgcctggtatctttatagtcctgtcgggtttcgccacctctgacttgagcgtcgatttttgtgatgctcgtcaggggggcggagcctatggaaaaacgccagcaacgcggcctttttacggttcctggccttttgctggccttttgctcacatgttctttcctgcgttatcccctgattctgtggataaccgtattaccgcctttgagtgagctgataccgctcgccgcagccgaacgaccgagcgcagcgagtcagtgagcgaggaagcggaagagcgcccaatacgcaaaccgcctctccccgcgcgttggccgattcattaatgcagctggcacgacaggtttcccgactggaaagcgggcagtgagcgcaacgcaattagctcactcattaggcaccccaggctttacactttatgcttccggctcgtatgttgtgtggaattgtgagcggataacaatttcacacaggaaacagctatgaccatgattacgcc

The maize ubiquitin promoter is shown as underlined **lowercase** letters. The CDS of humanized LbCas12a is shown as italic capital letters. The nucleus signal peptides coding sequences are shown in italic **lowercase** letters. The NOS terminator is shown as underlined capital letters. The Switch grass ubiquitin promoter and the intron are shown as blue capital letters when the 5’ UTR is shown in **lowercase** blue letters. The directed repeat of the LbCas12a crRNA are shown in red capital letters. The two BsaI cut sites for annealed guide sequence oligoes subcloning are shown as underlined blue capital letters. The 35S terminator is shown as magenta capital letters. The enzyme cut sites are all shown in lower case. KpnI is shown in red, EcoRI is shown in purple, XmaI is shown in yellow, SpeI is shown in orange. SacI is shown in green, BamHI is shown in light blue.

**pA9LbCas12acr-optimized (9lccop)**

aagcttGCATGCCTGCAGtgcagcgtgacccggtcgtgcccctctctagagataatgagcattgcatgtctaagttataaaaaattaccacatattttttttgtcacacttgtttgaagtgcagtttatctatctttatacatatatttaaactttactctacgaataatataatctatagtactacaataatatcagtgttttagagaatcatataaatgaacagttagacatggtctaaaggacaattgagtattttgacaacaggactctacagttttatctttttagtgtgcatgtgttctcctttttttttgcaaatagcttcacctatataatacttcatccattttattagtacatccatttagggtttagggttaatggtttttatagactaatttttttagtacatctattttattctattttagcctctaaattaagaaaactaaaactctattttagtttttttatttaataatttagatataaaatagaataaaataaagtgactaaaaattaaacaaataccctttaagaaattaaaaaaactaaggaaacatttttcttgtttcgagtagataatgccagcctgttaaacgccgtcgacgagtctaacggacaccaaccagcgaaccagcagcgtcgcgtcgggccaagcgaagcagacggcacggcatctctgtcgctgcctctggacccctctcgagagttccgctccaccgttggacttgctccgctgtcggcatccagaaattgcgtggcggagcggcagacgtgagccggcacggcaggcggcctcctcctcctctcacggcaccggcagctacgggggattcctttcccaccgctccttcgctttcccttcctcgcccgccgtaataaatagacaccccctccacaccctctttccccaacctcgtgttgttcggagcgcacacacacacaaccagatctcccccaaatccacccgtcggcacctccgcttcaaggtacgccgctcgtcctccccccccccccctctctaccttctctagatcggcgttccggtccatggttagggcccggtagttctacttctgttcatgtttgtgttagatccgtgtttgtgttagatccgtgctgctagcgttcgtacacggatgcgacctgtacgtcagacacgttctgattgctaacttgccagtgtttctctttggggaatcctgggatggctctagccgttccgcagacgggatcgatttcatgattttttttgtttcgttgcatagggtttggtttgcccttttcctttatttcaatatatgccgtgcacttgtttgtcgggtcatcttttcatgcttttttttgtcttggttgtgatgatgtggtctggttgggcggtcgttctagatcggagtagaattctgtttcaaactacctggtggatttattaattttggatctgtatgtgtgtgccatacatattcatagttacgaattgaagatgatggatggaaatatcgatctaggataggtatacatgttgatgcgggttttactgatgcatatacagagatgctttttgttcgcttggttgtgatgatgtggtgtggttgggcggtcgttcattcgttctagatcggagtagaatactgtttcaaactacctggtgtatttattaattttggaactgtatgtgtgtgtcatacatcttcatagttacgagtttaagatggatggaaatatcgatctaggataggtatacatgttgatgtgggttttactgatgcatatacatgatggcatatgcagcatctattcatatgctctaaccttgagtacctatctattataataaacaagtatgttttataattattttgatcttgatatacttggatgatggcatatgcagcagctatatgtggatttttttagccctgccttcatacgctatttatttgcttggtactgtttcttttgtcgatgctcaccctgttgtttggtgttacttctgcaggtcgacTCTAGAggatccTAggtaccgccaccATG*gctcctaagaagaagcggaagGTTGGTATTCACGGGGTGCCTGCGGCTTCAAAGCTCGAGAAATTCACCAACTGTTATTCGTTGAGCAAAACACTGCGGTTTAAAGCGATTCCAGTCGGCAAGACTCAAGAGAATATAGACAATAAGCGGCTGTTGGTGGAAGATGAAAAGCGCGCGGAAGACTACAAAGGGGTGAAGAAGTTGTTGGACAGATACTACCTCTCTTTTATCAATGATGTCTTGCACTCAATCAAATTGAAGAATCTGAACAACTACATCTCCCTCTTCAGAAAGAAAACAAGGACAGAAAAGGAGAATAAGGAACTTGAAAATTTGGAGATCAATCTGAGGAAAGAGATCGCGAAAGCCTTTAAAGGCAACGAAGGATACAAAAGTCTGTTCAAGAAGGATATAATTGAGACAATTTTGCCAGAGTTCCTCGATGACAAGGACGAGATTGCGCTGGTCAATTCGTTCAACGGATTCACAACAGCATTCACAGGCTTCTTTGATAATCGGGAAAATATGTTCTCTGAGGAGGCAAAGTCCACTTCTATTGCGTTCAGGTGTATCAATGAGAATCTCACTAGGTACATTTCCAACATGGATATCTTTGAGAAGGTTGACGCAATTTTTGACAAGCACGAAGTTCAGGAGATTAAGGAGAAGATCCTCAATTCCGATTATGACGTTGAGGACTTCTTCGAAGGTGAGTTTTTTAATTTCGTGCTCACTCAAGAGGGTATCGACGTGTATAATGCGATCATCGGTGGGTTCGTGACTGAGTCCGGTGAAAAGATTAAGGGATTGAACGAGTATATCAACCTTTACAACCAAAAGACGAAACAGAAGCTGCCAAAGTTCAAGCCTCTTTACAAACAGGTTCTTTCAGACCGCGAGTCACTCTCGTTCTATGGGGAGGGCTACACTTCGGATGAGGAAGTCCTGGAGGTGTTCAGGAATACTCTCAATAAGAATTCGGAGATTTTCTCTTCTATAAAAAAACTGGAAAAGTTGTTTAAGAATTTTGACGAATACTCTAGCGCCGGCATATTTGTGAAAAACGGCCCGGCCATATCAACGATAAGTAAAGATATCTTCGGCGAATGGAACGTGATCAGAGACAAATGGAACGCGGAGTATGACGATATTCACCTGAAGAAGAAGGCTGTCGTAACGGAGAAGTACGAGGATGATCGCAGGAAAAGCTTCAAAAAGATCGGAAGTTTCAGCCTGGAACAGTTGCAGGAGTATGCTGACGCCGATCTTAGCGTCGTCGAGAAGTTGAAGGAGATAATCATCCAAAAGGTCGACGAGATATATAAAGTCTATGGATCAAGTGAAAAACTGTTCGACGCCGACTTCGTTTTGGAGAAGTCCCTGAAGAAGAACGACGCTGTTGTTGCCATTATGAAGGATCTGCTCGACAGCGTGAAGAGTTTCGAGAACTATATTAAGGCTTTTTTCGGGGAGGGGAAGGAGACTAACAGAGATGAGTCCTTCTACGGAGACTTCGTCCTCGCGTACGATATACTCCTTAAGGTAGACCACATCTACGACGCAATCAGAAATTACGTGACACAAAAGCCGTACAGCAAGGACAAGTTCAAACTCTACTTCCAGAACCCCCAGTTCATGGGCGGCTGGGACAAGGACAAGGAAACGGATTACAGGGCTACGATCCTGAGGTATGGTTCAAAATACTACTTGGCGATTATGGACAAGAAGTACGCCAAGTGTCTCCAGAAGATTGACAAAGACGATGTCAATGGCAATTATGAGAAGATCAACTACAAGCTGCTTCCGGGTCCGAACAAGATGCTCCCAAAGGTTTTCTTCAGCAAGAAATGGATGGCCTACTATAACCCAAGCGAGGACATCCAGAAGATTTATAAGAACGGTACGTTCAAGAAGGGCGACATGTTCAATCTTAACGACTGTCACAAGCTGATCGACTTCTTCAAAGACTCAATTAGCCGGTACCCAAAGTGGTCTAACGCCTATGACTTCAACTTTTCGGAAACCGAGAAGTACAAGGATATAGCCGGATTTTATAGAGAGGTGGAAGAGCAGGGCTACAAGGTGTCATTCGAGTCCGCCAGCAAGAAGGAAGTGGACAAGCTCGTGGAAGAGGGTAAGCTCTACATGTTCCAGATTTATAATAAAGACTTTAGCGATAAGAGCCACGGGACACCTAATCTCCACACAATGTATTTCAAGCTGCTCTTCGACGAGAATAACCACGGCCAAATCAGGTTGTCAGGAGGGGCTGAACTCTTCATGCGGCGCGCTAGCCTTAAGAAGGAGGAGCTTGTAGTCCACCCTGCGAATAGTCCAATTGCGAATAAGAACCCGGACAATCCTAAAAAGACTACAACATTGAGCTACGACGTGTACAAGGATAAGAGGTTTTCCGAGGATCAGTACGAGCTCCACATCCCGATTGCGATCAACAAGTGCCCAAAGAATATTTTCAAGATAAACACAGAGGTGCGTGTACTCCTGAAGCATGACGACAATCCTTACGTCATTGGGATTGATCGGGGCGAGAGGAACCTCCTCTATATTGTGGTGGTGGACGGGAAGGGGAACATAGTCGAACAGTACTCCCTTAACGAAATAATTAACAATTTCAACGGCATCCGTATCAAGACCGACTACCATTCGTTGCTGGACAAGAAGGAGAAGGAGAGATTTGAGGCGCGGCAAAATTGGACAAGTATCGAGAACATCAAGGAACTCAAAGCAGGTTATATCTCTCAAGTTGTGCATAAGATATGCGAGCTGGTTGAGAAGTATGACGCAGTGATCGCTCTTGAGGACCTCAACTCGGGCTTTAAGAATTCTAGAGTTAAAGTGGAGAAGCAGGTCTATCAAAAGTTCGAGAAGATGCTTATAGATAAGCTCAACTACATGGTCGATAAGAAATCGAACCCATGTGCCACCGGCGGCGCACTCAAAGGTTACCAAATAACAAACAAATTCGAGTCCTTCAAATCGATGAGTACTCAGAATGGGTTCATATTTTATATACCGGCGTGGCTTACGTCTAAGATCGACCCGTCAACTGGTTTTGTCAACCTGTTGAAGACGAAATACACGTCCATTGCCGATTCGAAAAAGTTCATATCTAGTTTTGATCGTATTATGTACGTCCCAGAGGAAGATCTTTTCGAGTTTGCTCTCGACTACAAAAACTTTTCGCGGACCGATGCGGATTACATTAAAAAATGGAAACTCTATTCGTACGGCAACAGAATCAGGATTTTTCGCAACCCTAAGAAGAATAACGTCTTTGATTGGGAGGAAGTTTGCTTGACTAGCGCGTACAAGGAGCTCTTTAATAAGTATGGCATTAACTACCAACAGGGTGATATCAGAGCACTGCTTTGCGAACAATCTGACAAGGCTTTCTACTCATCCTTCATGGCTTTGATGAGCCTGATGCTCCAGATGAGAAATTCAATTACAGGCAGAACCGACGTGGATTTCTTGATCTCCCCGGTTAAAAATTCTGATGGCATCTTTTACGATAGCAGGAACTATGAAGCGCAAGAGAATGCGATTCTGCCAAAAAATGCAGACGCCAACGGTGCCTATAACATCGCCAGGAAAGTCCTGTGGGCGATCGGCCAGTTCAAAAAGGCCGAAGACGAAAAATTGGACAAGGTCAAAATCGCTATCAGCAACAAAGAGTGGCTGGAGTATGCTCAGACATCCGTAAAGCATaagcgtcctgctgccaccaaaaaggccggacaggctaagaaaaagaagTGA*cccggg**GAATTTCCCCGATCGTTCAAACATTTGGCAATAAAGTTTCTTAAGATTGAATCCTGTTGCCGGTCTTGCGATGATTATCATATAATTTCTGTTGAATTACGTTAAGCATGTAATAATTAACATGTAATGCATGACGTTATTTATGAGATGGGTTTTTATGATTAGAGTCCCGCAATTATACATTTAATACGCGATAGAAAACAAAATATAGCGCGCAAACTAGGATAAATTATCGCGCGCGGTGTCATCTATGTTACTAGATCGG**actagtCACGTCAGTGTTTGGTTTCCACTAGCACGAGTAGCGCAATCAGAAAATTTTCAATGCATGAAGTACTAAACGAAGTTTATTTAGAAATTTTTTTAAGAAATGAGTGTAATTTTTTGCGACGAATTTAATGACAATAATTAATCGATGATTGCCTACAGTAATGCTACAGTAACCAACCTCTAATCATGCGTCGAATGCGTCATTAGATTCGTCTCGCAAAATAGCACAAGAATTATGAAATTAATTTTACAAACTATTTTTATTTAATACTAATAATTAACTGTCAAAGTTTGTGCTACTCGCAAGAGTAGCGCGAACCAAACACGGCCTGGAGGAGCACGGTAACGGCGTCGACAAACTAACGGCCACCACCCGCCAACGCAAAGGAGACGGATGAGAGTTGACTTCTTGACGGTTCTCCACCCCTCTGTCTCTCTGTCACTGGGCCCTGGGTCCCCCTCTCGAAAGTTCCTCTGGCCGAAATTGCGCGGCGGAGACGAGGCGGGCGGAACCGTCACGGCAGAGGATTCCTTCCCCACCCTGCCTGGCCCGGCCATATATAAACAGCCACCGCCCCTCCCCGTTCCCCatcgcgtctcgtctcgtgttgttcccagaacacaaccaaaatccaaatcctcctcctcctcccgagcctcgtcgatccctcacccgcttcaagGTACGGCGATCCTCCTCTCCCTTCTCCCCTCGATCGATTATGCGTGTTCCGTTTCCGTTTCCGATCGAGCGAATCGATGGTTAGGACCCATGGGGGACCCATGGGGTGTCGTGTGGTGGTCTGGTTTGATCCGCGATATTTCTCCGTTCGTAGTGTAGATCTGATCGAATCCCTGGTGAAATCGTTGATCGTGCTATTCGTGTGAGGGTTCTTAGGTTTGGAGTTGTGGAGGTAGTTCTGATCGGTTTGTAGGTGAGATTTTCCCCATGATTTTGCTTGGCTCGTTTGTCTTGGTTAGATTAGATCTGCCCGCATTTTGTTCGATATTTCTGATGCAGATATGATGAATAATTTCGTCCTTGTATCCCGCGTCCGTATGTGTATTAAGTTTGCAGGTCCTAGTTAGGTTTTTCCTACTGATTTGTCTTATCCATTCTGTTTAGCTTGCAAGGTTTGGTAATGGTCCGGCATGTTTGTCTCTATAGATTAGAGTAGAATAAGATTATCTCAACAAGCTGTTGGCTTATCAATTTTGGATCTGCATGTGTTTCGCATCTATATCTTTGCAATTAAGATGGTAGATGGACATATGCTCCTGTTGAGTTGATGTTGTACCTTTTACCTGAGGTCTGAGGAACATGCATCCTCCTGCTACTTTGTGCTTATACAGATCATCAAGATTATGTAGCTAATATTCGATCAGTTTCTAGTATCTACATGGTAAACTTGCATGCACTTGCTACTTATTTTTGATATACTTGGATGATAACATATGCTGCTGGTTGATTCCTACCTACATGATGAACATTTTACAGGCCATTAGTGTCTGTCTGTATGTGTTGTTCCTGTTTGCTTCAGTCTATTTCTGTTTCATTCCTAGTTTATTGGTTCTCTGCTAGATACTTACCCTGCTGGGCTTAGTTATCATCTTATCTCGAATGCATTTTCATGTTTATAGATGAATATACACTCAGATAGGTGTAGATGTATGCTACTGTTTCTCTACGTTGCTGTAGGTTTTACCTGTGGCAACTGCATACTCCTGTTGCTTCGCTAGATATGTATGTGCTTATATAGATTAAGATATGTGTGATGGTTCTTTAGTATATCTGATGATCATGTATGCTCTTTTAACTTCTTGCTACACTTGGTAACATGCTGTGATGCTGTTTGTTGATTCTGTAGCACTACCAATGATGACCTTATCTCTCTTTGTATATGATGTTTCTGTTTGTTTGAGGCTTGTGTTACTGCTAGTTACTTACCCTGTTGCCTGGCTAATCTTCTGCAGaaattactgatgagtccgtgaggacgaaacgagtaagctcgtcTAATTTCTACTAAGTGTAGAT**AGAGACCGAGAGAGGGTCTCA**ggccggcatggtcccagcctcctcgctggcgccggctgggcaacatgcttcggcatggcgaatgggac**TCGAGTTTCTCCATAATAATGTGTGAGTAGTTCCCAGATAAGGGAATTAGGGTTCCTATAGGGTTTCGCTCATGTGTTGAGCATATAAGAAACCCTTAGTATGTATTTGTATTTGTAAAATACTTCTATCAATAAAATTTCTAATTCCTAAAACCAAAATCCAGTACTAAAATCCAGATCCCCCGAATTA**gagctcgaattcactggccgtcgttttacaacgtcgtgactgggaaaaccctggcgttacccaacttaatcgccttgcagcacatccccctttcgccagctggcgtaatagcgaagaggcccgcaccgatcgcccttcccaacagttgcgcagcctgaatggcgaatggcgcctgatgcggtattttctccttacgcatctgtgcggtatttcacaccgcatatggtgcactctcagtacaatctgctctgatgccgcatagttaagccagccccgacacccgccaacacccgctgacgcgccctgacgggcttgtctgctcccggcatccgcttacagacaagctgtgaccgtctccgggagctgcatgtgtcagaggttttcaccgtcatcaccgaaacgcgcgagacgaaagggcctcgtgatacgcctatttttataggttaatgtcatgataataatggtttcttagacgtcaggtggcacttttcggggaaatgtgcgcggaacccctatttgtttatttttctaaatacattcaaatatgtatccgctcatgagacaataaccctgataaatgcttcaataatattgaaaaaggaagagtatgagtattcaacatttccgtgtcgcccttattcccttttttgcggcattttgccttcctgtttttgctcacccagaaacgctggtgaaagtaaaagatgctgaagatcagttgggtgcacgagtgggttacatcgaactggatctcaacagcggtaagatccttgagagttttcgccccgaagaacgttttccaatgatgagcacttttaaagttctgctatgtggcgcggtattatcccgtattgacgccgggcaagagcaactcggtcgccgcatacactattctcagaatgacttggttgagtactcaccagtcacagaaaagcatcttacggatggcatgacagtaagagaattatgcagtgctgccataaccatgagtgataacactgcggccaacttacttctgacaacgatcggaggaccgaaggagctaaccgcttttttgcacaacatgggggatcatgtaactcgccttgatcgttgggaaccggagctgaatgaagccataccaaacgacgagcgtgacaccacgatgcctgtagcaatggcaacaacgttgcgcaaactattaactggcgaactacttactctagcttcccggcaacaattaatagactggatggaggcggataaagttgcaggaccacttctgcgctcggcccttccggctggctggtttattgctgataaatctggagccggtgagcgtggctctcgcggtatcattgcagcactggggccagatggtaagccctcccgtatcgtagttatctacacgacggggagtcaggcaactatggatgaacgaaatagacagatcgctgagataggtgcctcactgattaagcattggtaactgtcagaccaagtttactcatatatactttagattgatttaaaacttcatttttaatttaaaaggatctaggtgaagatcctttttgataatctcatgaccaaaatcccttaacgtgagttttcgttccactgagcgtcagaccccgtagaaaagatcaaaggatcttcttgagatcctttttttctgcgcgtaatctgctgcttgcaaacaaaaaaaccaccgctaccagcggtggtttgtttgccggatcaagagctaccaactctttttccgaaggtaactggcttcagcagagcgcagataccaaatactgttcttctagtgtagccgtagttaggccaccacttcaagaactctgtagcaccgcctacatacctcgctctgctaatcctgttaccagtggctgctgccagtggcgataagtcgtgtcttaccgggttggactcaagacgatagttaccggataaggcgcagcggtcgggctgaacggggggttcgtgcacacagcccagcttggagcgaacgacctacaccgaactgagatacctacagcgtgagctatgagaaagcgccacgcttcccgaagggagaaaggcggacaggtatccggtaagcggcagggtcggaacaggagagcgcacgagggagcttccagggggaaacgcctggtatctttatagtcctgtcgggtttcgccacctctgacttgagcgtcgatttttgtgatgctcgtcaggggggcggagcctatggaaaaacgccagcaacgcggcctttttacggttcctggccttttgctggccttttgctcacatgttctttcctgcgttatcccctgattctgtggataaccgtattaccgcctttgagtgagctgataccgctcgccgcagccgaacgaccgagcgcagcgagtcagtgagcgaggaagcggaagagcgcccaatacgcaaaccgcctctccccgcgcgttggccgattcattaatgcagctggcacgacaggtttcccgactggaaagcgggcagtgagcgcaacgcaattagctcactcattaggcaccccaggctttacactttatgcttccggctcgtatgttgtgtggaattgtgagcggataacaatttcacacaggaaacagctatgaccatgattacgcc

The maize ubiquitin promoter is shown as underlined **lowercase** letters. The CDS of rice codon optimized LbCas12a is shown as italic capital letters. The nucleus signal peptides coding sequences are shown in italic **lowercase** letters. The NOS terminator is shown as underlined capital letters. The Switch grass ubiquitin promoter and the intron are shown as blue capital letters when the 5’ UTR is shown in **lowercase** blue letters. The hammerhead ribozyme and the HDV ribozyme are shown as brown **lowercase** letters. The directed repeat of the LbCas12a crRNA are shown in red capital letters. The two BsaI cut sites for annealed guide sequence oligoes subcloning are shown as underlined blue capital letters. The 35S terminator is shown as magenta capital letters. The enzyme cut sites are all shown in lower case. KpnI is shown in red, EcoRI is shown in purple, XmaI is shown in yellow, SpeI is shown in orange. SacI is shown in green, BamHI is shown in light blue.

**pA9LbCas12acr-optimized2 (9lccop2)**

aagcttGCATGCCTGCAGtgcagcgtgacccggtcgtgcccctctctagagataatgagcattgcatgtctaagttataaaaaattaccacatattttttttgtcacacttgtttgaagtgcagtttatctatctttatacatatatttaaactttactctacgaataatataatctatagtactacaataatatcagtgttttagagaatcatataaatgaacagttagacatggtctaaaggacaattgagtattttgacaacaggactctacagttttatctttttagtgtgcatgtgttctcctttttttttgcaaatagcttcacctatataatacttcatccattttattagtacatccatttagggtttagggttaatggtttttatagactaatttttttagtacatctattttattctattttagcctctaaattaagaaaactaaaactctattttagtttttttatttaataatttagatataaaatagaataaaataaagtgactaaaaattaaacaaataccctttaagaaattaaaaaaactaaggaaacatttttcttgtttcgagtagataatgccagcctgttaaacgccgtcgacgagtctaacggacaccaaccagcgaaccagcagcgtcgcgtcgggccaagcgaagcagacggcacggcatctctgtcgctgcctctggacccctctcgagagttccgctccaccgttggacttgctccgctgtcggcatccagaaattgcgtggcggagcggcagacgtgagccggcacggcaggcggcctcctcctcctctcacggcaccggcagctacgggggattcctttcccaccgctccttcgctttcccttcctcgcccgccgtaataaatagacaccccctccacaccctctttccccaacctcgtgttgttcggagcgcacacacacacaaccagatctcccccaaatccacccgtcggcacctccgcttcaaggtacgccgctcgtcctccccccccccccctctctaccttctctagatcggcgttccggtccatggttagggcccggtagttctacttctgttcatgtttgtgttagatccgtgtttgtgttagatccgtgctgctagcgttcgtacacggatgcgacctgtacgtcagacacgttctgattgctaacttgccagtgtttctctttggggaatcctgggatggctctagccgttccgcagacgggatcgatttcatgattttttttgtttcgttgcatagggtttggtttgcccttttcctttatttcaatatatgccgtgcacttgtttgtcgggtcatcttttcatgcttttttttgtcttggttgtgatgatgtggtctggttgggcggtcgttctagatcggagtagaattctgtttcaaactacctggtggatttattaattttggatctgtatgtgtgtgccatacatattcatagttacgaattgaagatgatggatggaaatatcgatctaggataggtatacatgttgatgcgggttttactgatgcatatacagagatgctttttgttcgcttggttgtgatgatgtggtgtggttgggcggtcgttcattcgttctagatcggagtagaatactgtttcaaactacctggtgtatttattaattttggaactgtatgtgtgtgtcatacatcttcatagttacgagtttaagatggatggaaatatcgatctaggataggtatacatgttgatgtgggttttactgatgcatatacatgatggcatatgcagcatctattcatatgctctaaccttgagtacctatctattataataaacaagtatgttttataattattttgatcttgatatacttggatgatggcatatgcagcagctatatgtggatttttttagccctgccttcatacgctatttatttgcttggtactgtttcttttgtcgatgctcaccctgttgtttggtgttacttctgcaggtcgacTCTAGAggatccTAggtaccgccacc*ATGgctcctaagaagaagcggaagGTTGGTATTCACGGGGTGCCTGCGGCTTCAAAGCTCGAGAAATTCACCAACTGTTATTCGTTGAGCAAAACACTGCGGTTTAAAGCGATTCCAGTCGGCAAGACTCAAGAGAATATAGACAATAAGCGGCTGTTGGTGGAAGATGAAAAGCGCGCGGAAGACTACAAAGGGGTGAAGAAGTTGTTGGACAGATACTACCTCTCTTTTATCAATGATGTCTTGCACTCAATCAAATTGAAGAATCTGAACAACTACATCTCCCTCTTCAGAAAGAAAACAAGGACAGAAAAGGAGAATAAGGAACTTGAAAATTTGGAGATCAATCTGAGGAAAGAGATCGCGAAAGCCTTTAAAGGCAACGAAGGATACAAAAGTCTGTTCAAGAAGGATATAATTGAGACAATTTTGCCAGAGTTCCTCGATGACAAGGACGAGATTGCGCTGGTCAATTCGTTCAACGGATTCACAACAGCATTCACAGGCTTCTTTGATAATCGGGAAAATATGTTCTCTGAGGAGGCAAAGTCCACTTCTATTGCGTTCAGGTGTATCAATGAGAATCTCACTAGGTACATTTCCAACATGGATATCTTTGAGAAGGTTGACGCAATTTTTGACAAGCACGAAGTTCAGGAGATTAAGGAGAAGATCCTCAATTCCGATTATGACGTTGAGGACTTCTTCGAAGGTGAGTTTTTTAATTTCGTGCTCACTCAAGAGGGTATCGACGTGTATAATGCGATCATCGGTGGGTTCGTGACTGAGTCCGGTGAAAAGATTAAGGGATTGAACGAGTATATCAACCTTTACAACCAAAAGACGAAACAGAAGCTGCCAAAGTTCAAGCCTCTTTACAAACAGGTTCTTTCAGACCGCGAGTCACTCTCGTTCTATGGGGAGGGCTACACTTCGGATGAGGAAGTCCTGGAGGTGTTCAGGAATACTCTCAATAAGAATTCGGAGATTTTCTCTTCTATAAAAAAACTGGAAAAGTTGTTTAAGAATTTTGACGAATACTCTAGCGCCGGCATATTTGTGAAAAACGGCCCGGCCATATCAACGATAAGTAAAGATATCTTCGGCGAATGGAACGTGATCAGAGACAAATGGAACGCGGAGTATGACGATATTCACCTGAAGAAGAAGGCTGTCGTAACGGAGAAGTACGAGGATGATCGCAGGAAAAGCTTCAAAAAGATCGGAAGTTTCAGCCTGGAACAGTTGCAGGAGTATGCTGACGCCGATCTTAGCGTCGTCGAGAAGTTGAAGGAGATAATCATCCAAAAGGTCGACGAGATATATAAAGTCTATGGATCAAGTGAAAAACTGTTCGACGCCGACTTCGTTTTGGAGAAGTCCCTGAAGAAGAACGACGCTGTTGTTGCCATTATGAAGGATCTGCTCGACAGCGTGAAGAGTTTCGAGAACTATATTAAGGCTTTTTTCGGGGAGGGGAAGGAGACTAACAGAGATGAGTCCTTCTACGGAGACTTCGTCCTCGCGTACGATATACTCCTTAAGGTAGACCACATCTACGACGCAATCAGAAATTACGTGACACAAAAGCCGTACAGCAAGGACAAGTTCAAACTCTACTTCCAGAACCCCCAGTTCATGGGCGGCTGGGACAAGGACAAGGAAACGGATTACAGGGCTACGATCCTGAGGTATGGTTCAAAATACTACTTGGCGATTATGGACAAGAAGTACGCCAAGTGTCTCCAGAAGATTGACAAAGACGATGTCAATGGCAATTATGAGAAGATCAACTACAAGCTGCTTCCGGGTCCGAACAAGATGCTCCCAAAGGTTTTCTTCAGCAAGAAATGGATGGCCTACTATAACCCAAGCGAGGACATCCAGAAGATTTATAAGAACGGTACGTTCAAGAAGGGCGACATGTTCAATCTTAACGACTGTCACAAGCTGATCGACTTCTTCAAAGACTCAATTAGCCGGTACCCAAAGTGGTCTAACGCCTATGACTTCAACTTTTCGGAAACCGAGAAGTACAAGGATATAGCCGGATTTTATAGAGAGGTGGAAGAGCAGGGCTACAAGGTGTCATTCGAGTCCGCCAGCAAGAAGGAAGTGGACAAGCTCGTGGAAGAGGGTAAGCTCTACATGTTCCAGATTTATAATAAAGACTTTAGCGATAAGAGCCACGGGACACCTAATCTCCACACAATGTATTTCAAGCTGCTCTTCGACGAGAATAACCACGGCCAAATCAGGTTGTCAGGAGGGGCTGAACTCTTCATGCGGCGCGCTAGCCTTAAGAAGGAGGAGCTTGTAGTCCACCCTGCGAATAGTCCAATTGCGAATAAGAACCCGGACAATCCTAAAAAGACTACAACATTGAGCTACGACGTGTACAAGGATAAGAGGTTTTCCGAGGATCAGTACGAGCTCCACATCCCGATTGCGATCAACAAGTGCCCAAAGAATATTTTCAAGATAAACACAGAGGTGCGTGTACTCCTGAAGCATGACGACAATCCTTACGTCATTGGGATTGATCGGGGCGAGAGGAACCTCCTCTATATTGTGGTGGTGGACGGGAAGGGGAACATAGTCGAACAGTACTCCCTTAACGAAATAATTAACAATTTCAACGGCATCCGTATCAAGACCGACTACCATTCGTTGCTGGACAAGAAGGAGAAGGAGAGATTTGAGGCGCGGCAAAATTGGACAAGTATCGAGAACATCAAGGAACTCAAAGCAGGTTATATCTCTCAAGTTGTGCATAAGATATGCGAGCTGGTTGAGAAGTATGACGCAGTGATCGCTCTTGAGGACCTCAACTCGGGCTTTAAGAATTCTAGAGTTAAAGTGGAGAAGCAGGTCTATCAAAAGTTCGAGAAGATGCTTATAGATAAGCTCAACTACATGGTCGATAAGAAATCGAACCCATGTGCCACCGGCGGCGCACTCAAAGGTTACCAAATAACAAACAAATTCGAGTCCTTCAAATCGATGAGTACTCAGAATGGGTTCATATTTTATATACCGGCGTGGCTTACGTCTAAGATCGACCCGTCAACTGGTTTTGTCAACCTGTTGAAGACGAAATACACGTCCATTGCCGATTCGAAAAAGTTCATATCTAGTTTTGATCGTATTATGTACGTCCCAGAGGAAGATCTTTTCGAGTTTGCTCTCGACTACAAAAACTTTTCGCGGACCGATGCGGATTACATTAAAAAATGGAAACTCTATTCGTACGGCAACAGAATCAGGATTTTTCGCAACCCTAAGAAGAATAACGTCTTTGATTGGGAGGAAGTTTGCTTGACTAGCGCGTACAAGGAGCTCTTTAATAAGTATGGCATTAACTACCAACAGGGTGATATCAGAGCACTGCTTTGCGAACAATCTGACAAGGCTTTCTACTCATCCTTCATGGCTTTGATGAGCCTGATGCTCCAGATGAGAAATTCAATTACAGGCAGAACCGACGTGGATTTCTTGATCTCCCCGGTTAAAAATTCTGATGGCATCTTTTACGATAGCAGGAACTATGAAGCGCAAGAGAATGCGATTCTGCCAAAAAATGCAGACGCCAACGGTGCCTATAACATCGCCAGGAAAGTCCTGTGGGCGATCGGCCAGTTCAAAAAGGCCGAAGACGAAAAATTGGACAAGGTCAAAATCGCTATCAGCAACAAAGAGTGGCTGGAGTATGCTCAGACATCCGTAAAGCATaagcgtcctgctgccaccaaaaaggccggacaggctaagaaaaagaagTGAcccggg****GAATTTCCCCGATCGTTCAAACATTTGGCAATAAAGTTTCTTAAGATTGAATCCTGTTGCCGGTCTTGCGATGATTATCATATAATTTCTGTTGAATTACGTTAAGCATGTAATAATTAACATGTAATGCATGACGTTATTTATGAGATGGGTTTTTATGATTAGAGTCCCGCAATTATACATTTAATACGCGATAGAAAACAAAATATAGCGCGCAAACTAGGATAAATTATCGCGCGCGGTGTCATCTATGTTACTAGATCGG****actagtCACGTCAGTGTTTGGTTTCCACTAGCACGAGTAGCGCAATCAGAAAATTTTCAATGCATGAAGTACTAAACGAAGTTTATTTAGAAATTTTTTTAAGAAATGAGTGTAATTTTTTGCGACGAATTTAATGACAATAATTAATCGATGATTGCCTACAGTAATGCTACAGTAACCAACCTCTAATCATGCGTCGAATGCGTCATTAGATTCGTCTCGCAAAATAGCACAAGAATTATGAAATTAATTTTACAAACTATTTTTATTTAATACTAATAATTAACTGTCAAAGTTTGTGCTACTCGCAAGAGTAGCGCGAACCAAACACGGCCTGGAGGAGCACGGTAACGGCGTCGACAAACTAACGGCCACCACCCGCCAACGCAAAGGAGACGGATGAGAGTTGACTTCTTGACGGTTCTCCACCCCTCTGTCTCTCTGTCACTGGGCCCTGGGTCCCCCTCTCGAAAGTTCCTCTGGCCGAAATTGCGCGGCGGAGACGAGGCGGGCGGAACCGTCACGGCAGAGGATTCCTTCCCCACCCTGCCTGGCCCGGCCATATATAAACAGCCACCGCCCCTCCCCGTTCCCCatcgcgtctcgtctcgtgttgttcccagaacacaaccaaaatccaaatcctcctcctcctcccgagcctcgtcgatccctcacccgcttcaagGTACGGCGATCCTCCTCTCCCTTCTCCCCTCGATCGATTATGCGTGTTCCGTTTCCGTTTCCGATCGAGCGAATCGATGGTTAGGACCCATGGGGGACCCATGGGGTGTCGTGTGGTGGTCTGGTTTGATCCGCGATATTTCTCCGTTCGTAGTGTAGATCTGATCGAATCCCTGGTGAAATCGTTGATCGTGCTATTCGTGTGAGGGTTCTTAGGTTTGGAGTTGTGGAGGTAGTTCTGATCGGTTTGTAGGTGAGATTTTCCCCATGATTTTGCTTGGCTCGTTTGTCTTGGTTAGATTAGATCTGCCCGCATTTTGTTCGATATTTCTGATGCAGATATGATGAATAATTTCGTCCTTGTATCCCGCGTCCGTATGTGTATTAAGTTTGCAGGTCCTAGTTAGGTTTTTCCTACTGATTTGTCTTATCCATTCTGTTTAGCTTGCAAGGTTTGGTAATGGTCCGGCATGTTTGTCTCTATAGATTAGAGTAGAATAAGATTATCTCAACAAGCTGTTGGCTTATCAATTTTGGATCTGCATGTGTTTCGCATCTATATCTTTGCAATTAAGATGGTAGATGGACATATGCTCCTGTTGAGTTGATGTTGTACCTTTTACCTGAGGTCTGAGGAACATGCATCCTCCTGCTACTTTGTGCTTATACAGATCATCAAGATTATGTAGCTAATATTCGATCAGTTTCTAGTATCTACATGGTAAACTTGCATGCACTTGCTACTTATTTTTGATATACTTGGATGATAACATATGCTGCTGGTTGATTCCTACCTACATGATGAACATTTTACAGGCCATTAGTGTCTGTCTGTATGTGTTGTTCCTGTTTGCTTCAGTCTATTTCTGTTTCATTCCTAGTTTATTGGTTCTCTGCTAGATACTTACCCTGCTGGGCTTAGTTATCATCTTATCTCGAATGCATTTTCATGTTTATAGATGAATATACACTCAGATAGGTGTAGATGTATGCTACTGTTTCTCTACGTTGCTGTAGGTTTTACCTGTGGCAACTGCATACTCCTGTTGCTTCGCTAGATATGTATGTGCTTATATAGATTAAGATATGTGTGATGGTTCTTTAGTATATCTGATGATCATGTATGCTCTTTTAACTTCTTGCTACACTTGGTAACATGCTGTGATGCTGTTTGTTGATTCTGTAGCACTACCAATGATGACCTTATCTCTCTTTGTATATGATGTTTCTGTTTGTTTGAGGCTTGTGTTACTGCTAGTTACTTACCCTGTTGCCTGGCTAATCTTTAATTTCTACTAAGTGTAGAT****AGAGACCGAGAGAGGGTCTCA****TAATTTCTACTAAGTGTAGAT****CTCGAGTTTCTCCATAATAATGTGTGAGTAGTTCCCAGATAAGGGAATTAGGGTTCCTATAGGGTTTCGCTCATGTGTTGAGCATATAAGAAACCCTTAGTATGTATTTGTATTTGTAAAATACTTCTATCAATAAAATTTCTAATTCCTAAAACCAAAATCCAGTACTAAAATCCAGATCCCCCGAATTA****gagctcgaattcactggccgtcgttttacaacgtcgtgactgggaaaaccctggcgttacccaacttaatcgccttgcagcacatccccctttcgccagctggcgtaatagcgaagaggcccgcaccgatcgcccttcccaacagttgcgcagcctgaatggcgaatggcgcctgatgcggtattttctccttacgcatctgtgcggtatttcacaccgcatatggtgcactctcagtacaatctgctctgatgccgcatagttaagccagccccgacacccgccaacacccgctgacgcgccctgacgggcttgtctgctcccggcatccgcttacagacaagctgtgaccgtctccgggagctgcatgtgtcagaggttttcaccgtcatcaccgaaacgcgcgagacgaaagggcctcgtgatacgcctatttttataggttaatgtcatgataataatggtttcttagacgtcaggtggcacttttcggggaaatgtgcgcggaacccctatttgtttatttttctaaatacattcaaatatgtatccgctcatgagacaataaccctgataaatgcttcaataatattgaaaaaggaagagtatgagtattcaacatttccgtgtcgcccttattcccttttttgcggcattttgccttcctgtttttgctcacccagaaacgctggtgaaagtaaaagatgctgaagatcagttgggtgcacgagtgggttacatcgaactggatctcaacagcggtaagatccttgagagttttcgccccgaagaacgttttccaatgatgagcacttttaaagttctgctatgtggcgcggtattatcccgtattgacgccgggcaagagcaactcggtcgccgcatacactattctcagaatgacttggttgagtactcaccagtcacagaaaagcatcttacggatggcatgacagtaagagaattatgcagtgctgccataaccatgagtgataacactgcggccaacttacttctgacaacgatcggaggaccgaaggagctaaccgcttttttgcacaacatgggggatcatgtaactcgccttgatcgttgggaaccggagctgaatgaagccataccaaacgacgagcgtgacaccacgatgcctgtagcaatggcaacaacgttgcgcaaactattaactggcgaactacttactctagcttcccggcaacaattaatagactggatggaggcggataaagttgcaggaccacttctgcgctcggcccttccggctggctggtttattgctgataaatctggagccggtgagcgtggctctcgcggtatcattgcagcactggggccagatggtaagccctcccgtatcgtagttatctacacgacggggagtcaggcaactatggatgaacgaaatagacagatcgctgagataggtgcctcactgattaagcattggtaactgtcagaccaagtttactcatatatactttagattgatttaaaacttcatttttaatttaaaaggatctaggtgaagatcctttttgataatctcatgaccaaaatcccttaacgtgagttttcgttccactgagcgtcagaccccgtagaaaagatcaaaggatcttcttgagatcctttttttctgcgcgtaatctgctgcttgcaaacaaaaaaaccaccgctaccagcggtggtttgtttgccggatcaagagctaccaactctttttccgaaggtaactggcttcagcagagcgcagataccaaatactgttcttctagtgtagccgtagttaggccaccacttcaagaactctgtagcaccgcctacatacctcgctctgctaatcctgttaccagtggctgctgccagtggcgataagtcgtgtcttaccgggttggactcaagacgatagttaccggataaggcgcagcggtcgggctgaacggggggttcgtgcacacagcccagcttggagcgaacgacctacaccgaactgagatacctacagcgtgagctatgagaaagcgccacgcttcccgaagggagaaaggcggacaggtatccggtaagcggcagggtcggaacaggagagcgcacgagggagcttccagggggaaacgcctggtatctttatagtcctgtcgggtttcgccacctctgacttgagcgtcgatttttgtgatgctcgtcaggggggcggagcctatggaaaaacgccagcaacgcggcctttttacggttcctggccttttgctggccttttgctcacatgttctttcctgcgttatcccctgattctgtggataaccgtattaccgcctttgagtgagctgataccgctcgccgcagccgaacgaccgagcgcagcgagtcagtgagcgaggaagcggaagagcgcccaatacgcaaaccgcctctccccgcgcgttggccgattcattaatgcagctggcacgacaggtttcccgactggaaagcgggcagtgagcgcaacgcaattagctcactcattaggcaccccaggctttacactttatgcttccggctcgtatgttgtgtggaattgtgagcggataacaatttcacacaggaaacagctatgaccatgattacgcc*

The maize ubiquitin promoter is shown as underlined **lowercase** letters. The CDS of rice codon optimized LbCas12a is shown as italic capital letters. The nucleus signal peptides coding sequences are shown in italic **lowercase** letters. The NOS terminator is shown as underlined capital letters. The Switch grass ubiquitin promoter and the intron are shown as blue capital letters when the 5’ UTR is shown in **lowercase** blue letters. The directed repeat of the LbCas12a crRNA are shown in red capital letters. The two BsaI cut sites for annealed guide sequence oligoes subcloning are shown as underlined blue capital letters. The 35S terminator is shown as magenta capital letters. The enzyme cut sites are all shown in lower case. KpnI is shown in red, EcoRI is shown in purple, XmaI is shown in yellow, SpeI is shown in orange. SacI is shown in green, BamHI is shown in light blue.

**pBUN421x**

taaacgctcttttctcttaggtttacccgccaatatatcctgtcaaacactgatagtttaaactgaaggcgggaaacgacaatctgatccaagctcaagctaagctcacgtgacggaattaagcttcatgaatccaaaccacacggagttcaaattcccacagattaaggctcgtccgtcgcacaaggtaatgtgtgaatattatatctgtcgtgcaaaattgcctggcctgcacaattgctgttatagttggcggcagggagagttttaacattgactagcgtgctgataatttgtgagaaataataattgacaagtagatactgacatttgagaagagcttctgaactgttattagtaacaaaaatggaaagctgatgcacggaaaaaggaaagaaaaagccatacttttttttaggtaggaaaagaaaaagccatacgagactgatgtctctcagatgggccgggatctgtctatctagcaggcagcagcccaccaacctcacgggccagcaattacgagtccttctaaaagctcccgccgaggggcgctggcgctgctgtgcagcagcacgtctaacattagtcccacctcgccagtttacagggagcagaaccagcttataagcggaggcgcggcaccaagaagcgtgagaccaacccagtggacataagcctgttcggttcgtaagctgtaatgcaagtagcgtatgcgctcacgcaactggtccagaaccttgaccgaacgcagcggtggtaacggcgcagtggcggttttcatggcttgttatgactgtttttttggggtacagtctatgcctcgggcatccaagcagcaagcgcgttacgccgtgggtcgatgtttgatgttatggagcagcaacgatgttacgcagcagggcagtcgccctaaaacaaagttaaacatcATGGGGGAAGCGGTGATCGCCGAAGTATCGACTCAACTATCAGAGGTAGTTGGCGTCATCGAGCGCCATCTCGAACCGACGTTGCTGGCCGTACATTTGTACGGCTCCGCAGTGGATGGCGGCCTGAAGCCACACAGTGATATTGATTTGCTGGTTACGGTGACCGTAAGGCTTGATGAAACAACGCGGCGAGCTTTGATCAACGACCTTTTGGAAACTTCGGCTTCCCCTGGAGAGAGCGAGATTCTCCGCGCTGTAGAAGTCACCATTGTTGTGCACGACGACATCATTCCGTGGCGTTATCCAGCTAAGCGCGAACTGCAATTTGGAGAATGGCAGCGCAATGACATTCTTGCAGGTATCTTCGAGCCAGCCACGATCGACATTGATCTGGCTATCTTGCTGACAAAAGCAAGAGAACATAGCGTTGCCTTGGTAGGTCCAGCGGCGGAGGAACTCTTTGATCCGGTTCCTGAACAGGATCTATTTGAGGCGCTAAATGAAACCTTAACGCTATGGAACTCGCCGCCCGACTGGGCTGGCGATGAGCGAAATGTAGTGCTTACGTTGTCCCGCATTTGGTACAGCGCAGTAACCGGCAAAATCGCGCCGAAGGATGTCGCTGCCGACTGGGCAATGGAGCGCCTGCCGGCCCAGTATCAGCCCGTCATACTTGAAGCTAGACAGGCTTATCTTGGACAAGAAGAAGATCGCTTGGCCTCGCGCGCAGATCAGTTGGAAGAATTTGTCCACTACGTGAAAGGCGAGATCACCAAGGTAGTCGGCAAATAAtgtctagctagaaattcgttcaagccgacgccgcttcgcggcgcggcttaactcaagcgttagatgcactaagcacataattgctcacagccaaactatcaggtcaagtctgcttttattatttttaagcgtgcataataagccggtctcggttttagagctagaaatagcaagttaaaataaggctagtccgttatcaacttgaaaaagtggcaccgagtcggtgcttttttttttgtccttctgtttttttagtcagtctcttttttcagaagtacaacatcttttttttgtccttctgtttttttagtcagtctcttttttcagaagtacaacatcttttttttgtccttctgtttttttagtcagtctcttttttcagaagtacaacatcttttttttgtccttctgtttttttagtcagtctcttttttcagaagtactctatgatgtatcgttctgggaaatgtctgtctgtctacaaccccataatctatatttgcaatcacacatctaatattctctgtgacaagacagccgaacaagcttgatatcgaattcctgcagtgcagcgtgacccggtcgtgcccctctctagagataatgagcattgcatgtctaagttataaaaaattaccacatattttttttgtcacacttgtttgaagtgcagtttatctatctttatacatatatttaaactttactctacgaataatataatctatagtactacaataatatcagtgttttagagaatcatataaatgaacagttagacatggtctaaaggacaattgagtattttgacaacaggactctacagttttatctttttagtgtgcatgtgttctcctttttttttgcaaatagcttcacctatataatacttcatccattttattagtacatccatttagggtttagggttaatggtttttatagactaatttttttagtacatctattttattctattttagcctctaaattaagaaaactaaaactctattttagtttttttatttaataatttagatataaaatagaataaaataaagtgactaaaaattaaacaaataccctttaagaaattaaaaaaactaaggaaacatttttcttgtttcgagtagataatgccagcctgttaaacgccgtcgacgagtctaacggacaccaaccagcgaaccagcagcgtcgcgtcgggccaagcgaagcagacggcacggcatctctgtcgctgcctctggacccctctcgagagttccgctccaccgttggacttgctccgctgtcggcatccagaaatgcgtggcggagcggcagacgtgagccggcacggcaggcggcctcctcctcctctcacggcacggcagctacgggggattcctttcccaccgctccttcgctttcccttcctcgcccgccgtaataaatagacaccccctccacaccctctttccccaacctcgtgttgttcggagcgcacacacacacaaccagatctcccccaaatccacccgtcggcacctccgcttcaaggtacgccgctcgtcctccccccccccccctctctaccttctctagatcggcgttccggtccatggttagggcccggtagttctacttctgttcatgtttgtgttagatccgtgtttgtgttagatccgtgctgctagcgttcgtacacggatgcgacctgtacgtcagacacgttctgattgctaacttgccagtgtttctctttggggaatcctgggatggctctagccgttccgcagacgggatcgatttcatgattttttttgtttcgttgcatagggtttggtttgcccttttcctttatttcaatatatgccgtgcacttgtttgtcgggtcatcttttcatgcttttttttgtcttggttgtgatgatgtggtctggttgggcggtcgttctagatcggagtagaattctgtttcaaactacctggtggatttattaattttggatctgtatgtgtgtgccatacatattcatagttacgaattgaagatgatggatggaaatatcgatctaggataggtatacatgttgatgcgggttttactgatgcatatacagagatgctttttgttcgcttggttgtgatgatgtggtgtggttgggcggtcgttcattcgttctagatcggagtagaatactgtttcaaactacctggtgtatttattaattttggaactgtatgtgtgtgtcatacatcttcatagttacgagtttaagatggatggaaatatcgatctaggataggtatacatgttgatgtgggttttactgatgcatatacatgatggcatatgcagcatctattcatatgctctaaccttgagtacctatctattataataaacaagtatgttttataattattttgatcttgatatacttggatgatggcatatgcagcagctatatgtggatttttttagccctgccttcatacgctatttatttgcttggtactgtttcttttgtcgatgctcaccctgttgtttggtgttacttctgcagcccgggatggattacaaggaccacgacggggattacaaggaccacgacattgattacaaggatgatgatgacaagATGGCTCCGAAGAAGAAGAGGAAGGTTGGCATCCACGGGGTGCCAGCTGCT*gacaagaagtactcgatcggcctcgatattgggactaactctgttggctgggccgtgatcaccgacgagtacaaggtgccctcaaagaagttcaaggtcctgggcaacaccgatcggcattccatcaagaagaatctcattggcgctctcctgttcgacagcggcgagacggctgaggctacgcggctcaagcgcaccgcccgcaggcggtacacgcgcaggaagaatcgcatctgctacctgcaggagattttctccaacgagatggcgaaggttgacgattctttcttccacaggctggaggagtcattcctcgtggaggaggataagaagcacgagcggcatccaatcttcggcaacattgtcgacgaggttgcctaccacgagaagtaccctacgatctaccatctgcggaagaagctcgtggactccacagataaggcggacctccgcctgatctacctcgctctggcccacatgattaagttcaggggccatttcctgatcgagggggatctcaacccggacaatagcgatgttgacaagctgttcatccagctcgtgcagacgtacaaccagctcttcgaggagaaccccattaatgcgtcaggcgtcgacgcgaaggctatcctgtccgctaggctctcgaagtctcggcgcctcgagaacctgatcgcccagctgccgggcgagaagaagaacggcctgttcgggaatctcattgcgctcagcctggggctcacgcccaacttcaagtcgaatttcgatctcgctgaggacAccaagctgcagctctccaaggacacatacgacgatgacctggataacctcctggcccagatcggcgatcagtacgcggacctgttcctcgctgccaagaatctgtcggacgccatcctcctgtctgatattctcagggtgaacaccgagattacgaaggctccgctctcagcctccatgatcaagcTctacgacgagcaccatcaggatctgaccctcctgaaggcgctggtcaggcagcagctccccgagaagtacaaggagatcttcttcgatcagtcgaagaacggctacgctgggtacattgacggcggggcctctcaggaggagttctacaagttcatcaagccgattctggagaagatggacggcacggaggagctgctggtgaagctcaatcgcgaggacctcctgaggaagcagcggacattcgataacggcaTcatcccacaccagattcatctcggggagctgcacgctatcctgaggaggcaggaggacttctaccctttcctcaaggataaccgcgagaagatcgagaagattctgactttcaggatcccgtactacgtcggcccactcgctaggggcaactcccgcttcgcttggatgacccgcaagtcagaggagacgatcacgccgtggaacttcgagAaggtggtcgacaagggcgctagcgctcagtcgttcatcgagaggatgacgaatttcgacaagaacctgccaaatgagaaggtgctccctaagcactcgctcctgtacgagtacttcacagtctacaacgagctgactaaggtgaagtatgtgaccgagggcatgaggaagccggctttcctgtctggggaCcagaagaaggccatcgtggacctcctgttcaagaccaaccggaaggtcacggttaagcagctcaaggaggactacttcaagaagattgagtgcttcgattcggtcgagatctctggcgttgaggaccgcttcaacgcctccctggggacctaccacgatctcctgaagatcattaaggataaggacttcctggacaacgaggagaatgaggatatcctcgaggacattgtgctgacactcactctgttcgaggaccgggagatgatcgaggagcgcctgaagacttacgcccatctcttcgatgacaaggtcatgaagcagctcaagaggaggaggtacaccggctgggggaggctgagcaggaagctcatcaacggcattcgggacaagcagtccgggaagacgatcctcgacttcctgaagagcgatggcttcgcgaaccgcaatttcatCcagctgattcacgatgacagcctcacattcaaggaggatatccagaaggctcaggtgagcggccagggggactcgctgcacgagcatatcgcgaacctcgctggctcgccagctatcaagaaggggattctgcagaccgtgaaggttgtggacgagctggtgaaggtcatgggcaggcacaagcctgagaacatcgtcattgagatggcccgggagaatcagaccacgcagaagggccagaagaactcacgcgagaggatgaagaggatcgaggagggcattaaggagctggggtcccagatcctcaaggagcacccggtggagaacacgcagctgcagaatgagaagctctacctgtactacctccagaatggccgcgatatgtatgtggaccaggagctggatattaacaggctcagcgattacgacgtcgatcatatcgttccacagtcattcctgaaggatgactccattgacaacaaggtcctcaccaggtcggacaagaaccggggcaagtctgataatgttccttcagaggaggtcgttaagaagatgaagaactactggcgccagctcctgaatgccaagctgatcacgcagcggaagttcgataacctcacaaaggctgagaggggcgggctctctgagctggacaaggcgggcttcatcaagaggcagctggtcgagacacggcagatcactaagcacgttgcgcagattctcgactcacggatgaacactaagtacgatgagaatgacaagctgatccgcgaggtgaaggtcatcaccctgaagtcaaagctcgtctccgacttcaggaaggatttccagttctacaaggttcgggagatcaacaattaccaccatgcccatgacgcgtacctgaacgcggtggtcggcacagctctgatcaagaagtacccaaagctcgagagcgagttcgtgtacggggactacaaggtttacgatgtgaggaagatgatcgccaagtcggagcaggagattggcaaggctaccgccaagtacttcttctactctaacattatgaatttcttcaagacagagatcactctggccaatggcgagatccggaagcgccccctcatcgagacgaacggcgagacgggggagatcgtgtgggacaagggcagggatttcgcgaccgtcaggaaggttctctccatgccacaagtgaatatcgtcaagaagacagaggtccagactggcgggttctctaaggagtcaattctgcctaagcggaacagcgacaagctcatcgcccgcaagaaggactgggatccgaagaagtacggcgggttcgacagccccactgtggcctactcggtcctggttgtggcgaaggttgagaagggcaagtccaagaagctcaagagcgtgaaggagctgctggggatcacgattatggagcgctccagcttcgagaagaacccgatcgatttcctggaggcgaagggctacaaggaggtgaagaaggacctgatcattaagctccccaagtactcactcttcgagctggagaacggcaggaagcggatgctggcttccgctggcgTgctgcagaaggggaacgagctggctctgccgtccaagtatgtgaacttcctctacctggcctcccactacgagaagctcaagggcagccccgaggacaacgagcagaagcagctgttcgtcgagcagcacaagcattacctcgacgagatcattgagcagatttccgagttctccaagcgcgtgatcctggccgacgcgaatctggataaggtcctctccgcgtacaacaagcaccgcgacaagccaatcagggagcaggctgagaatatcattcatctcttcaccctgacgaacctcggcgcccctgctgctttcaagtacttcgacacaactatcgatcgcaagaggtacacaagcactaaggaggtcctggacgcgaccctcatccaccagtcgattaccggcctctacgagacgcgcatcgacctgtctcagctcgggggcgac*AAGCGGCCAGCGGCGACGAAGAAGGCGGGGCAGGCGAAGAAGAAGAAGtgaaggcctccatacaagtattggggatcc*GAATTTCCCCGATCGTTCAAACATTTGGCAATAAAGTTTCTTAAGATTGAATCCTGTTGCCGGTCTTGCGATGATTATCATATAATTTCTGTTGAATTACGTTAAGCATGTAATAATTAACATGTAATGCATGACGTTATTTATGAGATGGGTTTTTATGATTAGAGTCCCGCAATTATACATTTAATACGCGATAGAAAACAAAATATAGCGCGCAAACTAGGATAAATTATCGCGCGCGGTGTCATCTATGTTACTAGATCGG*gaattcactggccgtcgttttacaacgtcgtgactgggaaaaccctggcgttacccaacttaatcgccttgcagcacatccccctttcgccaggggtaccgagctctagaattcgaggcggtttgcgtattggctagagcagcttgccaacatggtggagcacgacactctcgtctactccaagaatatcaaagatacagtctcagaagaccaaagggctattgagacttttcaacaaagggtaatatcgggaaacctcctcggattccattgcccagctatctgtcacttcatcaaaaggacagtagaaaaggaaggtggcacctacaaatgccatcattgcgataaaggaaaggctatcgttcaagatgcctctgccgacagtggtcccaaagatggacccccacccacgaggagcatcgtggaaaaagaagacgttccaaccacgtcttcaaagcaagtggattgatgtgataacatggtggagcacgacactctcgtctactccaagaatatcaaagatacagtctcagaagaccaaagggctattgagacttttcaacaaagggtaatatcgggaaacctcctcggattccattgcccagctatctgtcacttcatcaaaaggacagtagaaaaggaaggtggcacctacaaatgccatcattgcgataaaggaaaggctatcgttcaagatgcctctgccgacagtggtcccaaagatggacccccacccacgaggagcatcgtggaaaaagaagacgttccaaccacgtcttcaaagcaagtggattgatgtgatatctccactgacgtaagggatgacgcacaatcccactatccttcgcaagaccttcctctatataaggaagttcatttcatttggagaggacacgctgaaatcaccagtctctctctacaaatctatctctctcgagtctaccatgagcccagaacgacgcccggccgacatccgccgtgccaccgaggcggacatgccggcggtctgcaccatcgtcaaccactacatcgagacaagcacggtcaacttccgtaccgagccgcaggaaccgcaggagtggacggacgacctcgtccgtctgcgggagcgctatccctggctcgtcgccgaggtggacggcgaggtcgccggcatcgcctacgcgggcccctggaaggcacgcaacgcctacgactggacggccgagtcgaccgtgtacgtctccccccgccaccagcggacgggactgggctccacgctctacacccacctgctgaagtccctggaggcacagggcttcaagagcgtggtcgctgtcatcgggctgcccaacgacccgagcgtgcgcatgcacgaggcgctcggatatgccccccgcggcatgctgcgggcggccggcttcaagcacgggaactggcatgacgtgggtttctggcagctggacttcagcctgccggtaccgccccgtccggtcctgcccgtcaccgagatttgactcgagtttctccataataatgtgtgagtagttcccagataagggaattagggttcctatagggtttcgctcatgtgttgagcatataagaaacccttagtatgtatttgtatttgtaaaatacttctatcaataaaatttctaattcctaaaaccaaaatccagtactaaaatccagatcccccgaattaattcggcgttaattcagtacattaaaaacgtccgcaatgtgttattaagttgtcactagtcaggttaactcaattcggcgttaattcagtacattaaaaacgtccgcaatgtgttattaagttgtctaagcgtcaatttgtttacaccacaatatatcctgccaccagccagccaacagctccccgaccggcagctcggcacaaaatcaccactcgatacaggcagcccatcagtccgggacggcgtcagcgggagagccgttgtaaggcggcagactttgctcatgttaccgatgctattcggaagaacggcaactaagctgccgggtttgaaacacggatgatctcgcggagggtagcatgttgattgtaacgatgacagagcgttgctgcctgtgatcacttaagtaactaactaacaggaagagtttgtagaaacgcaaaaaggccatccgtcaggatggccttctgcttagtttgatgcctggcagtttatggcgggcgtcctgcccgccacttcaccgacaaacaacagataaaacgaaaggcccagtcttccgactgagcctttcgttttatttgatgcctggcagttccctactctcgcttagtagttagacgtccccgagatccatgctagaccatgaatccagaagcccgagaggttgccgcctttcgggctttttctttttcaaaaaaaaaaatttataaaacgatctgttgcggccggccgccgggttgtgggcaaaggcgctcgacggtgggcaaccgcttgcggttgtccacgggcggagccggtgcgcgtagcgcattgtccacaagccaagggcgaccaataattgatatatatattcataattgaaaagctaattgaacatactacttgctgtaactacttgccggagcgaggggtgtttgcaagctgttgatctgaaagggctattagcgttctcacgtgcctttttgattagcgatttcacgtgaccttattagcgatttcacgtactccgattagcgatttcacgtaccctgattagcgatttcacgtggatagtttttggagcgggccggaaagccccgtgaatcaaggctttgcggggcattagcggtttcacgtggataactaccctctatccacaggcttccggggataaaaaagcccgctcgacggcgggctgttggatggggatctagcggtaatacggttatccacagaatcaggggataacgcaggaaagaacatgtgagcaaaaggccagcaaaaggccaggaaccgtaaaaaggccgcgttgctggcgtttttccataggctccgcccccctgacgagcatcacaaaaatcgacgctcaagtcagaggtggcgaaacccgacaggactataaagataccaggcgtttccccctggaagctccctcgtgcgctctcctgttccgaccctgccgcttaccggatacctgtccgcctttctcccttcgggaagcgtggcgctttctcatagctcacgctgtaggtatctcagttcggtgtaggtcgttcgctccaagctgggctgtgtgcacgaaccccccgttcagcccgaccgctgcgccttatccggtaactatcgtcttgagtccaacccggtaagacacgacttatcgccactggcagcagccactggtaacaggattagcagagcgaggtatgtaggcggtgctacagagttcttgaagtggtggcctaactacggctacactagaagaacagtatttggtatctgcgctctgctgaagccagttaccttcggaaaaagagttggtagctcttgatccggcaaacaaaccaccgctggtagcggtggtttttttgtttgcaagcagcagattacgcgcagaaaaaaaggatctcaagaagatcctttgatcttttctaccgggtctgacgctcagtggaacggggcccaatctgaataatgttacaaccaattaaccaattctgattagaaaaactcatcgagcatcaaatgaaactgcaatttattcatatcaggattatcaataccatatttttgaaaaagccgtttctgtaatgaaggagaaaactcaccgaggcagttccataggatggcaagatcctggtatcggtctgcgattccgactcgtccaacatcaatacaacctattaatttcccctcgtcaaaaataaggttatcaagtgagaaatcaccatgagtgacgactgaatccggtgagaatggcaaaagtttatgcatttctttccagacttgttcaacaggccagccattacgctcgtcatcaaaatcactcgcatcaaccaaaccgttattcattcgtgattgcgcctgagcgagacgaaatacgcgatcgctgttaaaaggacaattacaaacaggaatcgaatgcaaccggcgcagggacactgccagcgcatcaacaatattttcacctgaatcaggatattcttctaatacctggaatgctgtttttccggggatcgcagtggtgagtaaccatgcatcatcaggagtacggataaaatgcttgatggtcggaagaggcataaattccgtcagccagtttagtctgaccatctcatctgtaacatcattggcaacgctacctttgccatgtttcagaaacaactctggcgcatcgggcttcccatacaagcgatagattgtcgcacctgattgcccgacattatcgcgagcccatttatacccatataaatcagcatccatgttggaatttaatcgcggcctcgacgtttcccgttgaatatggctcataacaccccttgtattactgtttatgtaagcagacagttttattgttcatgatgatatatttttatcttgtgcaatgtaacatcagagattttgagacacgggccagagctgcagtttgatcccgaggggaaccctgtggttgacatgcacatacaaatggacgaacggataaaccttttcacgcccttttaaatatccgttattctaa

The two T-DNA borders are highlighted with purple color. The wheat U3 promoter is shown in purple letters, the wheat U3 terminator is underlined and highlighted with grey color. The two BsaI cut sites for annealed guide sequence oligoes subcloning are highlighted with dark green. The CDS of spectinomycin-resistance gene is shown in grey highlighted capital letters. The maize ubiquitin promoter is highlighted with yellow color. The CDS of maize codon optimized SpCas9 is shown as italic lowercase letters when the mutated nucleotides for getting xCas9 are shown in red capital letters. The 3xflag tag is highlighted with green color. The nucleus signal peptides coding sequences are shown in underlined capital letters. The NOS terminator is shown as italic capital letters. The Bar gene expression cassettes are highlighted with grey color. The enzyme cut site XmaI is shown in light blue, StuI is shown in brown.

**pBUN421NG**

taaacgctcttttctcttaggtttacccgccaatatatcctgtcaaacactgatagtttaaactgaaggcgggaaacgacaatctgatccaagctcaagctaagctcacgtgacggaattaagcttcatgaatccaaaccacacggagttcaaattcccacagattaaggctcgtccgtcgcacaaggtaatgtgtgaatattatatctgtcgtgcaaaattgcctggcctgcacaattgctgttatagttggcggcagggagagttttaacattgactagcgtgctgataatttgtgagaaataataattgacaagtagatactgacatttgagaagagcttctgaactgttattagtaacaaaaatggaaagctgatgcacggaaaaaggaaagaaaaagccatacttttttttaggtaggaaaagaaaaagccatacgagactgatgtctctcagatgggccgggatctgtctatctagcaggcagcagcccaccaacctcacgggccagcaattacgagtccttctaaaagctcccgccgaggggcgctggcgctgctgtgcagcagcacgtctaacattagtcccacctcgccagtttacagggagcagaaccagcttataagcggaggcgcggcaccaagaagcgtgagaccaacccagtggacataagcctgttcggttcgtaagctgtaatgcaagtagcgtatgcgctcacgcaactggtccagaaccttgaccgaacgcagcggtggtaacggcgcagtggcggttttcatggcttgttatgactgtttttttggggtacagtctatgcctcgggcatccaagcagcaagcgcgttacgccgtgggtcgatgtttgatgttatggagcagcaacgatgttacgcagcagggcagtcgccctaaaacaaagttaaacatcATGGGGGAAGCGGTGATCGCCGAAGTATCGACTCAACTATCAGAGGTAGTTGGCGTCATCGAGCGCCATCTCGAACCGACGTTGCTGGCCGTACATTTGTACGGCTCCGCAGTGGATGGCGGCCTGAAGCCACACAGTGATATTGATTTGCTGGTTACGGTGACCGTAAGGCTTGATGAAACAACGCGGCGAGCTTTGATCAACGACCTTTTGGAAACTTCGGCTTCCCCTGGAGAGAGCGAGATTCTCCGCGCTGTAGAAGTCACCATTGTTGTGCACGACGACATCATTCCGTGGCGTTATCCAGCTAAGCGCGAACTGCAATTTGGAGAATGGCAGCGCAATGACATTCTTGCAGGTATCTTCGAGCCAGCCACGATCGACATTGATCTGGCTATCTTGCTGACAAAAGCAAGAGAACATAGCGTTGCCTTGGTAGGTCCAGCGGCGGAGGAACTCTTTGATCCGGTTCCTGAACAGGATCTATTTGAGGCGCTAAATGAAACCTTAACGCTATGGAACTCGCCGCCCGACTGGGCTGGCGATGAGCGAAATGTAGTGCTTACGTTGTCCCGCATTTGGTACAGCGCAGTAACCGGCAAAATCGCGCCGAAGGATGTCGCTGCCGACTGGGCAATGGAGCGCCTGCCGGCCCAGTATCAGCCCGTCATACTTGAAGCTAGACAGGCTTATCTTGGACAAGAAGAAGATCGCTTGGCCTCGCGCGCAGATCAGTTGGAAGAATTTGTCCACTACGTGAAAGGCGAGATCACCAAGGTAGTCGGCAAATAAtgtctagctagaaattcgttcaagccgacgccgcttcgcggcgcggcttaactcaagcgttagatgcactaagcacataattgctcacagccaaactatcaggtcaagtctgcttttattatttttaagcgtgcataataagccggtctcggttttagagctagaaatagcaagttaaaataaggctagtccgttatcaacttgaaaaagtggcaccgagtcggtgcttttttttttgtccttctgtttttttagtcagtctcttttttcagaagtacaacatcttttttttgtccttctgtttttttagtcagtctcttttttcagaagtacaacatcttttttttgtccttctgtttttttagtcagtctcttttttcagaagtacaacatcttttttttgtccttctgtttttttagtcagtctcttttttcagaagtactctatgatgtatcgttctgggaaatgtctgtctgtctacaaccccataatctatatttgcaatcacacatctaatattctctgtgacaagacagccgaacaagcttgatatcgaattcctgcagtgcagcgtgacccggtcgtgcccctctctagagataatgagcattgcatgtctaagttataaaaaattaccacatattttttttgtcacacttgtttgaagtgcagtttatctatctttatacatatatttaaactttactctacgaataatataatctatagtactacaataatatcagtgttttagagaatcatataaatgaacagttagacatggtctaaaggacaattgagtattttgacaacaggactctacagttttatctttttagtgtgcatgtgttctcctttttttttgcaaatagcttcacctatataatacttcatccattttattagtacatccatttagggtttagggttaatggtttttatagactaatttttttagtacatctattttattctattttagcctctaaattaagaaaactaaaactctattttagtttttttatttaataatttagatataaaatagaataaaataaagtgactaaaaattaaacaaataccctttaagaaattaaaaaaactaaggaaacatttttcttgtttcgagtagataatgccagcctgttaaacgccgtcgacgagtctaacggacaccaaccagcgaaccagcagcgtcgcgtcgggccaagcgaagcagacggcacggcatctctgtcgctgcctctggacccctctcgagagttccgctccaccgttggacttgctccgctgtcggcatccagaaatgcgtggcggagcggcagacgtgagccggcacggcaggcggcctcctcctcctctcacggcacggcagctacgggggattcctttcccaccgctccttcgctttcccttcctcgcccgccgtaataaatagacaccccctccacaccctctttccccaacctcgtgttgttcggagcgcacacacacacaaccagatctcccccaaatccacccgtcggcacctccgcttcaaggtacgccgctcgtcctccccccccccccctctctaccttctctagatcggcgttccggtccatggttagggcccggtagttctacttctgttcatgtttgtgttagatccgtgtttgtgttagatccgtgctgctagcgttcgtacacggatgcgacctgtacgtcagacacgttctgattgctaacttgccagtgtttctctttggggaatcctgggatggctctagccgttccgcagacgggatcgatttcatgattttttttgtttcgttgcatagggtttggtttgcccttttcctttatttcaatatatgccgtgcacttgtttgtcgggtcatcttttcatgcttttttttgtcttggttgtgatgatgtggtctggttgggcggtcgttctagatcggagtagaattctgtttcaaactacctggtggatttattaattttggatctgtatgtgtgtgccatacatattcatagttacgaattgaagatgatggatggaaatatcgatctaggataggtatacatgttgatgcgggttttactgatgcatatacagagatgctttttgttcgcttggttgtgatgatgtggtgtggttgggcggtcgttcattcgttctagatcggagtagaatactgtttcaaactacctggtgtatttattaattttggaactgtatgtgtgtgtcatacatcttcatagttacgagtttaagatggatggaaatatcgatctaggataggtatacatgttgatgtgggttttactgatgcatatacatgatggcatatgcagcatctattcatatgctctaaccttgagtacctatctattataataaacaagtatgttttataattattttgatcttgatatacttggatgatggcatatgcagcagctatatgtggatttttttagccctgccttcatacgctatttatttgcttggtactgtttcttttgtcgatgctcaccctgttgtttggtgttacttctgcagcccgggatggattacaaggaccacgacggggattacaaggaccacgacattgattacaaggatgatgatgacaagATGGCTCCGAAGAAGAAGAGGAAGGTTGGCATCCACGGGGTGCCAGCTGCT*gacaagaagtactcgatcggcctcgatattgggactaactctgttggctgggccgtgatcaccgacgagtacaaggtgccctcaaagaagttcaaggtcctgggcaacaccgatcggcattccatcaagaagaatctcattggcgctctcctgttcgacagcggcgagacggctgaggctacgcggctcaagcgcaccgcccgcaggcggtacacgcgcaggaagaatcgcatctgctacctgcaggagattttctccaacgagatggcgaaggttgacgattctttcttccacaggctggaggagtcattcctcgtggaggaggataagaagcacgagcggcatccaatcttcggcaacattgtcgacgaggttgcctaccacgagaagtaccctacgatctaccatctgcggaagaagctcgtggactccacagataaggcggacctccgcctgatctacctcgctctggcccacatgattaagttcaggggccatttcctgatcgagggggatctcaacccggacaatagcgatgttgacaagctgttcatccagctcgtgcagacgtacaaccagctcttcgaggagaaccccattaatgcgtcaggcgtcgacgcgaaggctatcctgtccgctaggctctcgaagtctcggcgcctcgagaacctgatcgcccagctgccgggcgagaagaagaacggcctgttcgggaatctcattgcgctcagcctggggctcacgcccaacttcaagtcgaatttcgatctcgctgaggacgccaagctgcagctctccaaggacacatacgacgatgacctggataacctcctggcccagatcggcgatcagtacgcggacctgttcctcgctgccaagaatctgtcggacgccatcctcctgtctgatattctcagggtgaacaccgagattacgaaggctccgctctcagcctccatgatcaagcgctacgacgagcaccatcaggatctgaccctcctgaaggcgctggtcaggcagcagctccccgagaagtacaaggagatcttcttcgatcagtcgaagaacggctacgctgggtacattgacggcggggcctctcaggaggagttctacaagttcatcaagccgattctggagaagatggacggcacggaggagctgctggtgaagctcaatcgcgaggacctcctgaggaagcagcggacattcgataacggcagcatcccacaccagattcatctcggggagctgcacgctatcctgaggaggcaggaggacttctaccctttcctcaaggataaccgcgagaagatcgagaagattctgactttcaggatcccgtactacgtcggcccactcgctaggggcaactcccgcttcgcttggatgacccgcaagtcagaggagacgatcacgccgtggaacttcgaggaggtggtcgacaagggcgctagcgctcagtcgttcatcgagaggatgacgaatttcgacaagaacctgccaaatgagaaggtgctccctaagcactcgctcctgtacgagtacttcacagtctacaacgagctgactaaggtgaagtatgtgaccgagggcatgaggaagccggctttcctgtctggggagcagaagaaggccatcgtggacctcctgttcaagaccaaccggaaggtcacggttaagcagctcaaggaggactacttcaagaagattgagtgcttcgattcggtcgagatctctggcgttgaggaccgcttcaacgcctccctggggacctaccacgatctcctgaagatcattaaggataaggacttcctggacaacgaggagaatgaggatatcctcgaggacattgtgctgacactcactctgttcgaggaccgggagatgatcgaggagcgcctgaagacttacgcccatctcttcgatgacaaggtcatgaagcagctcaagaggaggaggtacaccggctgggggaggctgagcaggaagctcatcaacggcattcgggacaagcagtccgggaagacgatcctcgacttcctgaagagcgatggcttcgcgaaccgcaatttcatgcagctgattcacgatgacagcctcacattcaaggaggatatccagaaggctcaggtgagcggccagggggactcgctgcacgagcatatcgcgaacctcgctggctcgccagctatcaagaaggggattctgcagaccgtgaaggttgtggacgagctggtgaaggtcatgggcaggcacaagcctgagaacatcgtcattgagatggcccgggagaatcagaccacgcagaagggccagaagaactcacgcgagaggatgaagaggatcgaggagggcattaaggagctggggtcccagatcctcaaggagcacccggtggagaacacgcagctgcagaatgagaagctctacctgtactacctccagaatggccgcgatatgtatgtggaccaggagctggatattaacaggctcagcgattacgacgtcgatcatatcgttccacagtcattcctgaaggatgactccattgacaacaaggtcctcaccaggtcggacaagaaccggggcaagtctgataatgttccttcagaggaggtcgttaagaagatgaagaactactggcgccagctcctgaatgccaagctgatcacgcagcggaagttcgataacctcacaaaggctgagaggggcgggctctctgagctggacaaggcgggcttcatcaagaggcagctggtcgagacacggcagatcactaagcacgttgcgcagattctcgactcacggatgaacactaagtacgatgagaatgacaagctgatccgcgaggtgaaggtcatcaccctgaagtcaaagctcgtctccgacttcaggaaggatttccagttctacaaggttcgggagatcaacaattaccaccatgcccatgacgcgtacctgaacgcggtggtcggcacagctctgatcaagaagtacccaaagctcgagagcgagttcgtgtacggggactacaaggtttacgatgtgaggaagatgatcgccaagtcggagcaggagattggcaaggctaccgccaagtacttcttctactctaacattatgaatttcttcaagacagagatcactctggccaatggcgagatccggaagcgccccctcatcgagacgaacggcgagacgggggagatcgtgtgggacaagggcagggatttcgcgaccgtcaggaaggttctctccatgccacaagtgaatatcgtcaagaagacagaggtccagactggcgggttctctaaggagtcaattcGgcctaagcggaacagcgacaagctcatcgcccgcaagaaggactgggatccgaagaagtacggcgggttcgTcagccccactgtggcctactcggtcctggttgtggcgaaggttgagaagggcaagtccaagaagctcaagagcgtgaaggagctgctggggatcacgattatggagcgctccagcttcgagaagaacccgatcgatttcctggaggcgaagggctacaaggaggtgaagaaggacctgatcattaagctccccaagtactcactcttcgagctggagaacggcaggaagcggatgctggcttccgctCgcTTCctgcagaaggggaacgagctggctctgccgtccaagtatgtgaacttcctctacctggcctcccactacgagaagctcaagggcagccccgaggacaacgagcagaagcagctgttcgtcgagcagcacaagcattacctcgacgagatcattgagcagatttccgagttctccaagcgcgtgatcctggccgacgcgaatctggataaggtcctctccgcgtacaacaagcaccgcgacaagccaatcagggagcaggctgagaatatcattcatctcttcaccctgacgaacctcggcgcccctCGtgctttcaagtacttcgacacaactatcgatcgcaagGTgtacaGaagcactaaggaggtcctggacgcgaccctcatccaccagtcgattaccggcctctacgagacgcgcatcgacctgtctcagctcgggggcgac*AAGCGGCCAGCGGCGACGAAGAAGGCGGGGCAGGCGAAGAAGAAGAAGtgaaggcctccatacaagtattggggatcc*GAATTTCCCCGATCGTTCAAACATTTGGCAATAAAGTTTCTTAAGATTGAATCCTGTTGCCGGTCTTGCGATGATTATCATATAATTTCTGTTGAATTACGTTAAGCATGTAATAATTAACATGTAATGCATGACGTTATTTATGAGATGGGTTTTTATGATTAGAGTCCCGCAATTATACATTTAATACGCGATAGAAAACAAAATATAGCGCGCAAACTAGGATAAATTATCGCGCGCGGTGTCATCTATGTTACTAGATCGG*gaattcactggccgtcgttttacaacgtcgtgactgggaaaaccctggcgttacccaacttaatcgccttgcagcacatccccctttcgccaggggtaccgagctctagaattcgaggcggtttgcgtattggctagagcagcttgccaacatggtggagcacgacactctcgtctactccaagaatatcaaagatacagtctcagaagaccaaagggctattgagacttttcaacaaagggtaatatcgggaaacctcctcggattccattgcccagctatctgtcacttcatcaaaaggacagtagaaaaggaaggtggcacctacaaatgccatcattgcgataaaggaaaggctatcgttcaagatgcctctgccgacagtggtcccaaagatggacccccacccacgaggagcatcgtggaaaaagaagacgttccaaccacgtcttcaaagcaagtggattgatgtgataacatggtggagcacgacactctcgtctactccaagaatatcaaagatacagtctcagaagaccaaagggctattgagacttttcaacaaagggtaatatcgggaaacctcctcggattccattgcccagctatctgtcacttcatcaaaaggacagtagaaaaggaaggtggcacctacaaatgccatcattgcgataaaggaaaggctatcgttcaagatgcctctgccgacagtggtcccaaagatggacccccacccacgaggagcatcgtggaaaaagaagacgttccaaccacgtcttcaaagcaagtggattgatgtgatatctccactgacgtaagggatgacgcacaatcccactatccttcgcaagaccttcctctatataaggaagttcatttcatttggagaggacacgctgaaatcaccagtctctctctacaaatctatctctctcgagtctaccatgagcccagaacgacgcccggccgacatccgccgtgccaccgaggcggacatgccggcggtctgcaccatcgtcaaccactacatcgagacaagcacggtcaacttccgtaccgagccgcaggaaccgcaggagtggacggacgacctcgtccgtctgcgggagcgctatccctggctcgtcgccgaggtggacggcgaggtcgccggcatcgcctacgcgggcccctggaaggcacgcaacgcctacgactggacggccgagtcgaccgtgtacgtctccccccgccaccagcggacgggactgggctccacgctctacacccacctgctgaagtccctggaggcacagggcttcaagagcgtggtcgctgtcatcgggctgcccaacgacccgagcgtgcgcatgcacgaggcgctcggatatgccccccgcggcatgctgcgggcggccggcttcaagcacgggaactggcatgacgtgggtttctggcagctggacttcagcctgccggtaccgccccgtccggtcctgcccgtcaccgagatttgactcgagtttctccataataatgtgtgagtagttcccagataagggaattagggttcctatagggtttcgctcatgtgttgagcatataagaaacccttagtatgtatttgtatttgtaaaatacttctatcaataaaatttctaattcctaaaaccaaaatccagtactaaaatccagatcccccgaattaattcggcgttaattcagtacattaaaaacgtccgcaatgtgttattaagttgtcactagtcaggttaactcaattcggcgttaattcagtacattaaaaacgtccgcaatgtgttattaagttgtctaagcgtcaatttgtttacaccacaatatatcctgccaccagccagccaacagctccccgaccggcagctcggcacaaaatcaccactcgatacaggcagcccatcagtccgggacggcgtcagcgggagagccgttgtaaggcggcagactttgctcatgttaccgatgctattcggaagaacggcaactaagctgccgggtttgaaacacggatgatctcgcggagggtagcatgttgattgtaacgatgacagagcgttgctgcctgtgatcacttaagtaactaactaacaggaagagtttgtagaaacgcaaaaaggccatccgtcaggatggccttctgcttagtttgatgcctggcagtttatggcgggcgtcctgcccgccacttcaccgacaaacaacagataaaacgaaaggcccagtcttccgactgagcctttcgttttatttgatgcctggcagttccctactctcgcttagtagttagacgtccccgagatccatgctagaccatgaatccagaagcccgagaggttgccgcctttcgggctttttctttttcaaaaaaaaaaatttataaaacgatctgttgcggccggccgccgggttgtgggcaaaggcgctcgacggtgggcaaccgcttgcggttgtccacgggcggagccggtgcgcgtagcgcattgtccacaagccaagggcgaccaataattgatatatatattcataattgaaaagctaattgaacatactacttgctgtaactacttgccggagcgaggggtgtttgcaagctgttgatctgaaagggctattagcgttctcacgtgcctttttgattagcgatttcacgtgaccttattagcgatttcacgtactccgattagcgatttcacgtaccctgattagcgatttcacgtggatagtttttggagcgggccggaaagccccgtgaatcaaggctttgcggggcattagcggtttcacgtggataactaccctctatccacaggcttccggggataaaaaagcccgctcgacggcgggctgttggatggggatctagcggtaatacggttatccacagaatcaggggataacgcaggaaagaacatgtgagcaaaaggccagcaaaaggccaggaaccgtaaaaaggccgcgttgctggcgtttttccataggctccgcccccctgacgagcatcacaaaaatcgacgctcaagtcagaggtggcgaaacccgacaggactataaagataccaggcgtttccccctggaagctccctcgtgcgctctcctgttccgaccctgccgcttaccggatacctgtccgcctttctcccttcgggaagcgtggcgctttctcatagctcacgctgtaggtatctcagttcggtgtaggtcgttcgctccaagctgggctgtgtgcacgaaccccccgttcagcccgaccgctgcgccttatccggtaactatcgtcttgagtccaacccggtaagacacgacttatcgccactggcagcagccactggtaacaggattagcagagcgaggtatgtaggcggtgctacagagttcttgaagtggtggcctaactacggctacactagaagaacagtatttggtatctgcgctctgctgaagccagttaccttcggaaaaagagttggtagctcttgatccggcaaacaaaccaccgctggtagcggtggtttttttgtttgcaagcagcagattacgcgcagaaaaaaaggatctcaagaagatcctttgatcttttctaccgggtctgacgctcagtggaacggggcccaatctgaataatgttacaaccaattaaccaattctgattagaaaaactcatcgagcatcaaatgaaactgcaatttattcatatcaggattatcaataccatatttttgaaaaagccgtttctgtaatgaaggagaaaactcaccgaggcagttccataggatggcaagatcctggtatcggtctgcgattccgactcgtccaacatcaatacaacctattaatttcccctcgtcaaaaataaggttatcaagtgagaaatcaccatgagtgacgactgaatccggtgagaatggcaaaagtttatgcatttctttccagacttgttcaacaggccagccattacgctcgtcatcaaaatcactcgcatcaaccaaaccgttattcattcgtgattgcgcctgagcgagacgaaatacgcgatcgctgttaaaaggacaattacaaacaggaatcgaatgcaaccggcgcagggacactgccagcgcatcaacaatattttcacctgaatcaggatattcttctaatacctggaatgctgtttttccggggatcgcagtggtgagtaaccatgcatcatcaggagtacggataaaatgcttgatggtcggaagaggcataaattccgtcagccagtttagtctgaccatctcatctgtaacatcattggcaacgctacctttgccatgtttcagaaacaactctggcgcatcgggcttcccatacaagcgatagattgtcgcacctgattgcccgacattatcgcgagcccatttatacccatataaatcagcatccatgttggaatttaatcgcggcctcgacgtttcccgttgaatatggctcataacaccccttgtattactgtttatgtaagcagacagttttattgttcatgatgatatatttttatcttgtgcaatgtaacatcagagattttgagacacgggccagagctgcagtttgatcccgaggggaaccctgtggttgacatgcacatacaaatggacgaacggataaaccttttcacgcccttttaaatatccgttattctaa

**Figure S1.** **The sequences of plasmids used in this study.** The two T-DNA borders are highlighted with purple color. The wheat U3 promoter is shown in purple letters, the wheat U3 terminator is underlined and highlighted with grey color. The two BsaI cut sites for annealed guide sequence oligoes subcloning are highlighted with dark green. The CDS of spectinomycin-resistance gene is shown in grey highlighted capital letters. The maize ubiquitin promoter is highlighted with yellow color. The CDS of maize codon optimized SpCas9 is shown as italic lowercase letters when the mutated nucleotides for getting Cas9-NG are shown in red capital letters. The 3xflag tag is highlighted with green color. The nucleus signal peptides coding sequences are shown in underlined capital letters. The NOS terminator is shown as italic capital letters. The Bar gene expression cassettes are highlighted with grey color. The enzyme cut site XmaI is shown in light blue, StuI is shown in brown.
