# Supplementary material for: Expanding the range of editable targets in the wheat genome using the variants of the Cas12a and Cas9 nucleases
Source: Plant Biotechnol J. 2021 Jul 28;19(12):2428–41. doi: 10.1111/pbi.13669 (PMC8633491; doi:10.1111/pbi.13669)
Supplement: Supplementary file 2 — Figure S2 Gene editing efficiency comparison between the single‐target and multiple‐target CRISPR‐LbCas12a constructs. [file PBI-19-2428-s007.pptx]

## Slide 1
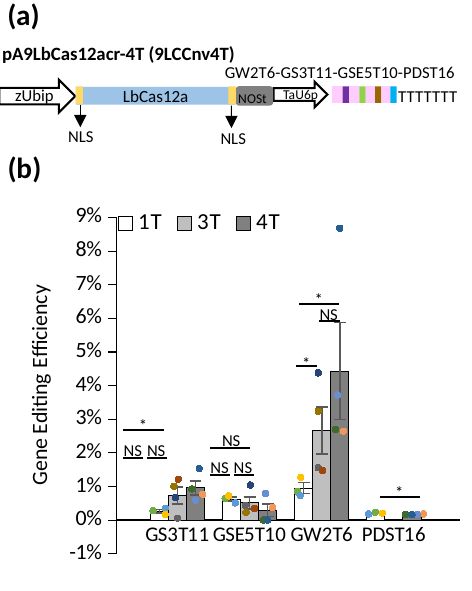

(a)
pA9LbCas12acr-4T (9LCCnv4T)
GW2T6-GS3T11-GSE5T10-PDST16
zUbip
TaU6p
NOSt
LbCas12a
TTTTTTT
NLS
NLS
(b)
### Chart
| Category | 1T | 3T | 4T | 1Tr1 | 1Tr2 | 1Tr3 | 3Tr1 | 3Tr2 | 3Tr3 | 3Tr4 | 4Tr1 | 4Tr2 | 4Tr3 | 4Tr4 |
|---|---|---|---|---|---|---|---|---|---|---|---|---|---|---|
| GS3T11 | 0.00264863480410873 | 0.007369774607215973 | 0.00955665016555743 | 0.0016663889351774706 | 0.0035168810289389067 | 0.00276263444820981 | 0.006687391555812609 | 0.012143782383419689 | 0.0005672793283412752 | 0.010080645161290322 | 0.015314414301787723 | 0.009248554913294797 | 0.005966587112171838 | 0.00769704433497537 |
| GSE5T10 | 0.00624285036057137 | 0.005137263039549063 | 0.0029338365960789575 | 0.007215007215007215 | 0.005122950819672132 | 0.006390593047034765 | 0.01042752867570386 | 0.0034482758620689655 | 0.0043859649122807015 | 0.0022872827081427266 | 0.0 | 0.0 | 0.007987220447284345 | 0.0037481259370314842 |
| GW2T6 | 0.009507993048526916 | 0.026630151483005474 | 0.044334159944579815 | 0.012698950220115138 | 0.0072780203784570605 | 0.008547008547008548 | 0.043792840822543797 | 0.014749262536873156 | 0.015574383254423124 | 0.03240411931818182 | 0.08680555555555555 | 0.02689486552567237 | 0.03718393653941497 | 0.02645228215767635 |
| PDST16 | 0.0020801581983841814 | None | 0.001700529047790013 | 0.0020709140126917444 | 0.0018248670561727257 | 0.002344693526288075 | None | None | None | None | 0.0016155948855589338 | 0.0016283421723086635 | 0.0017163799863362693 | 0.0018417991469561845 |*
NS
*
*
NS
NS
NS
NS
NS
*
