# Supplementary figures and images for: Expanding the range of editable targets in the wheat genome using the variants of the Cas12a and Cas9 nucleases
Source: Plant Biotechnol J. 2021 Jul 28;19(12):2428–41. doi: 10.1111/pbi.13669 (PMC8633491; doi:10.1111/pbi.13669)

## Slide 1
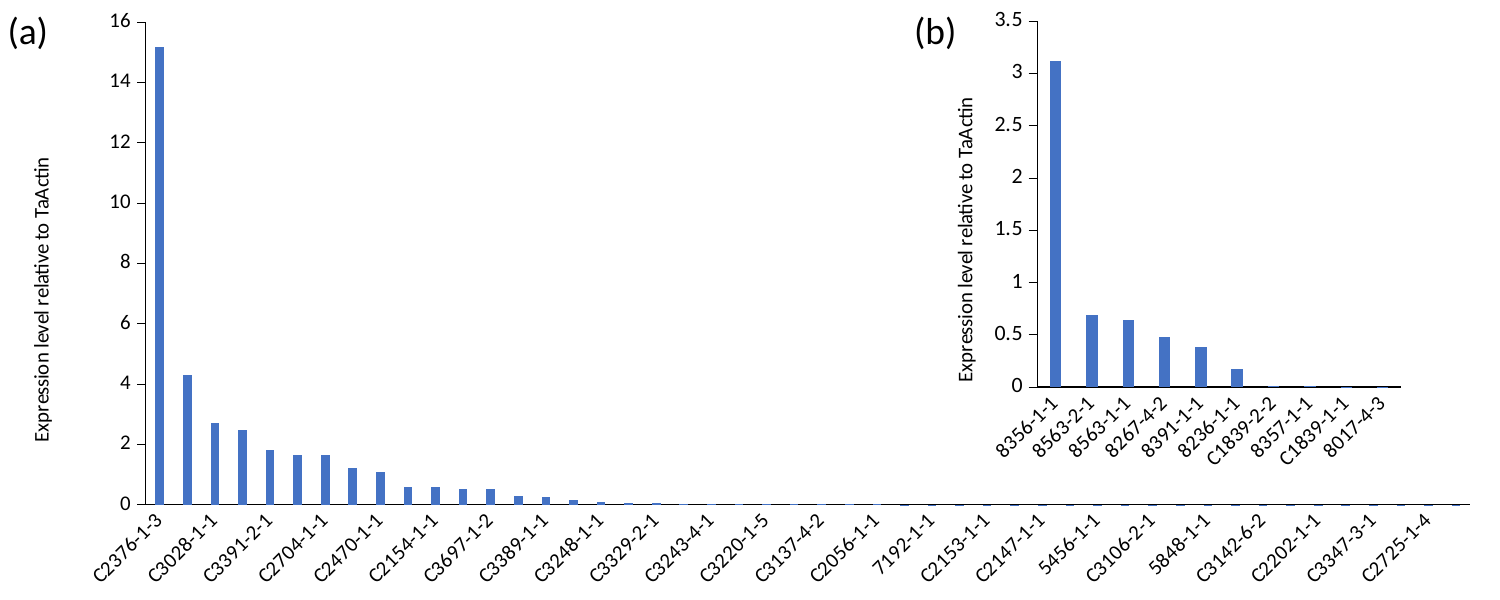

### Chart
| Category | |
|---|---|
| C2376-1-3 | 15.17450729791179 |
| C3216-6-2 | 4.291850463341432 |
| C3028-1-1 | 2.716269699868222 |
| 6920-1-3 | 2.4679075742104337 |
| C3391-2-1 | 1.8002037019889232 |
| C3216-1-1 | 1.6617032678422017 |
| C2704-1-1 | 1.6611573107393858 |
| C3387-1-1 | 1.2233373946252215 |
| C2470-1-1 | 1.091439623067515 |
| C3215-5-1 | 0.5875525622786264 |
| C2154-1-1 | 0.5792593561757499 |
| C3409-2-1 | 0.5346960386253253 |
| C3697-1-2 | 0.5070695893603633 |
| C2514-1-1 | 0.2807178089981424 |
| C3389-1-1 | 0.2610713883084203 |
| C2200-1-4 | 0.1513699713756312 |
| C3248-1-1 | 0.08885653228631812 |
| C3248-4-1 | 0.048550578313371194 |
| C3329-2-1 | 0.04454331133020654 |
| C3329-1-1 | 0.034403707682114024 |
| C3243-4-1 | 0.022661024939089277 |
| 6930-1-4 | 0.014649014439730022 |
| C3220-1-5 | 0.008899575666375078 |
| 5848-2-1 | 0.007420973264797288 |
| C3137-4-2 | 0.007234142032135073 |
| C3248-2-2 | 0.006808487994941625 |
| C2056-1-1 | 0.006667064817251649 |
| 5679-1-1 | 0.005389612598605578 |
| 7192-1-1 | 0.005273267780610007 |
| C3220-4-1 | 0.005161975929744147 |
| C2153-1-1 | 0.004881472532797469 |
| C2705-1-1 | 0.004797965065013632 |
| C2147-1-1 | 0.0037557289676586323 |
| C3407-1-1 | 0.003384544007440804 |
| 5456-1-1 | 0.002466393604507954 |
| C2999-5-1 | 0.0023623761227773935 |
| C3106-2-1 | 0.0020524172825460026 |
| C3220-1-1 | 0.001946891454540406 |
| 5848-1-1 | 0.0019445954102883533 |
| C3351-4-1 | 0.001932417421810778 |
| C3142-6-2 | 0.0018352406952665075 |
| C2726-1-1 | 0.0018213140676146071 |
| C2202-1-1 | 0.0018152606256735071 |
| C2999-2-1 | 0.0016172488881142258 |
| C3347-3-1 | 0.001048445502464828 |
| C3347-2-1 | 0.0008310188289598146 |
| C2725-1-4 | 0.000811771817907303 |
| 5765-1-3 | 0.0004271935429475173 |(a)
(b)
### Chart
| Category | |
|---|---|
| 8356-1-1 | 3.124105217694781 |
| 8563-2-1 | 0.6901966769402106 |
| 8563-1-1 | 0.637201451802665 |
| 8267-4-2 | 0.48269783035823094 |
| 8391-1-1 | 0.38249938716651194 |
| 8236-1-1 | 0.171351986976795 |
| C1839-2-2 | 0.009523534641528733 |
| 8357-1-1 | 0.008121525463034358 |
| C1839-1-1 | 0.004674883990520012 |
| 8017-4-3 | 0.0012119885841760417 |

Supplement: Supplementary file 3 — Figure S3 LbCas12a expression level in T1 progeny of (a) 9LCCnv8T and (b) 9LCCnvGS3T11 transgenic plants. [file PBI-19-2428-s013.pptx]

## Slide 1
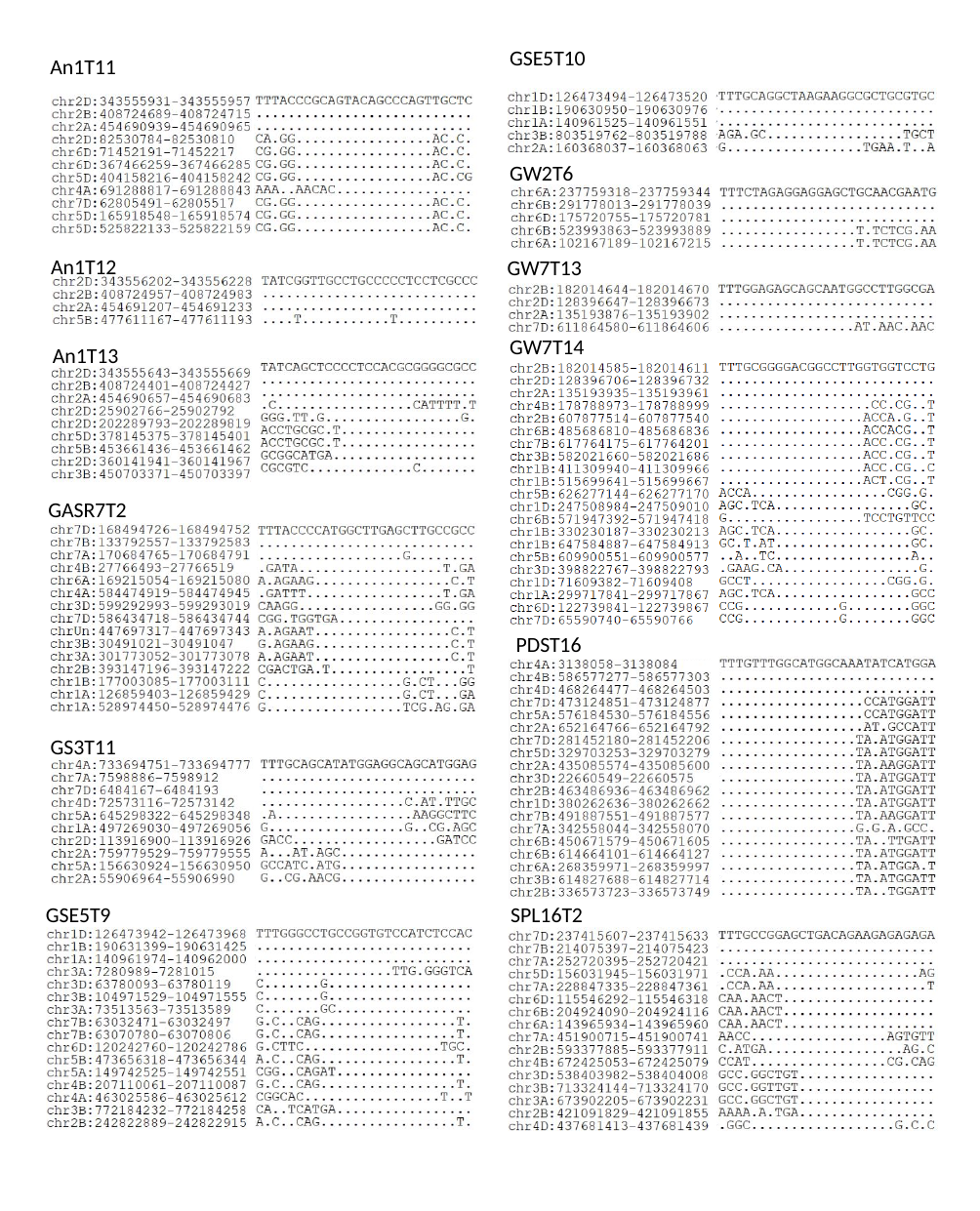

GSE5T10
An1T11
GW2T6
An1T12
GW7T13
GW7T14
An1T13
GASR7T2
PDST16
GS3T11
GSE5T9
SPL16T2

Supplement: Supplementary file 5 — Figure S5 The alignment of off‐target sequences with 12 LbCpf1 targets selected based on Chinese Spring RefSeqv1.0. [file PBI-19-2428-s015.pptx]
