# Supplementary material for: Expanding the range of editable targets in the wheat genome using the variants of the Cas12a and Cas9 nucleases
Source: Plant Biotechnol J. 2021 Jul 28;19(12):2428–41. doi: 10.1111/pbi.13669 (PMC8633491; doi:10.1111/pbi.13669)
Supplement: Supplementary file 4 — Figure S4 The gene editing efficiency in MGE transgenic plants with and without high‐temperature treatment. [file PBI-19-2428-s009.pptx]

## Slide 1
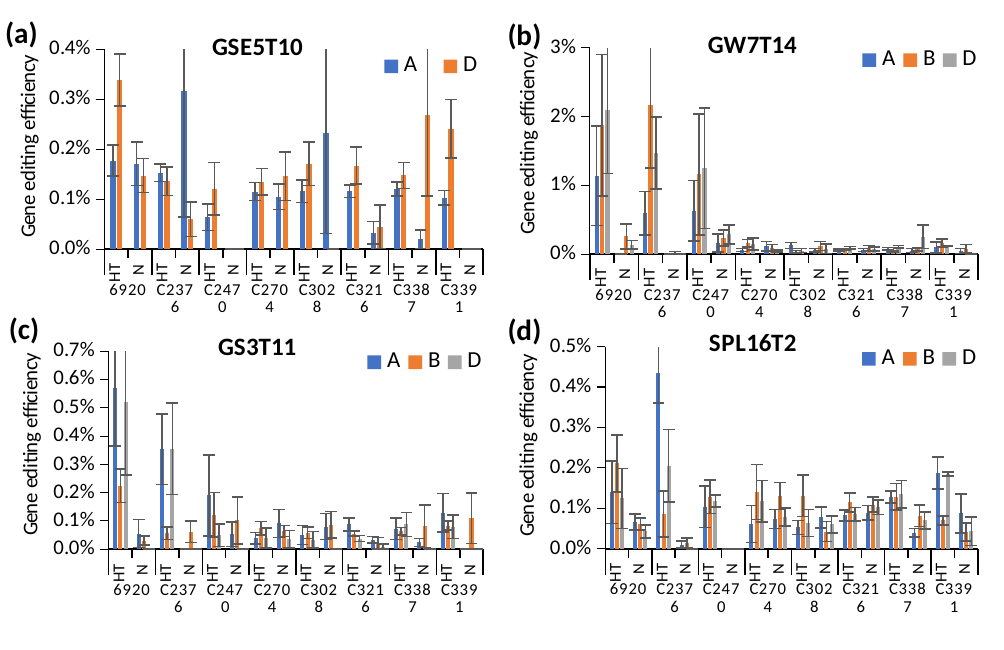

(a)
(b)
### Chart: GSE5T10
| Category | A | D |
|---|---|---|
| HT | 0.00177261263921688 | 0.003379643136873352 |
| N | 0.001705452624270115 | 0.001471558542092648 |
| HT | 0.001526912233275895 | 0.001354890216420758 |
| N | 0.00316206723977222 | 0.0005988382988954865 |
| HT | 0.0006382436926709872 | 0.0012040368357431666 |
| N | 0.0 | 0.0 |
| HT | 0.0011485055622862692 | 0.0013493305471471214 |
| N | 0.001046986212743856 | 0.0014611102989519529 |
| HT | 0.0011560606319775658 | 0.0017057745094540616 |
| N | 0.002314814814814815 | 0.0 |
| HT | 0.0011584878410947398 | 0.0016695528353059628 |
| N | 0.0003279457324471609 | 0.0004492362982929019 |
| HT | 0.0012027764954351016 | 0.0014728045033736575 |
| N | 0.00019924287706714428 | 0.0026826759847837158 |
| HT | 0.0010282436081627967 | 0.0024098854387068465 |
| N | 0.0 | 0.0 |
### Chart: GW7T14
| Category | A | B | D |
|---|---|---|---|
| HT | 0.01141414498104237 | 0.018728599598411423 | 0.02101502947377189 |
| N | 0.0 | 0.002561802232854872 | 0.001363042407468632 |
| HT | 0.005951313334030305 | 0.02163663198821573 | 0.01469914840899843 |
| N | 0.0 | 0.000195924764890282 | 0.0 |
| HT | 0.00631030224440537 | 0.011596506988953424 | 0.012466612935307717 |
| N | 0.0015948963317384366 | 0.0023707282161388433 | 0.002858822058936944 |
| HT | 0.0005857186064030832 | 0.0015952942532647771 | 0.001445991847231468 |
| N | 0.001133353865106252 | 0.0008688333885127408 | 0.0004350402412039942 |
| HT | 0.0012497491030600788 | 0.00033715981493204173 | 0.0005194457753607915 |
| N | 0.0005610255897640949 | 0.0011860938143429375 | 0.00079113924050633 |
| HT | 0.0005779869920868272 | 0.0007004086578365143 | 0.0009083335789209725 |
| N | 0.0006052405055110631 | 0.000883418026103081 | 0.0007376293278440975 |
| HT | 0.0007938821496268656 | 0.0004103142474758899 | 0.0010957495303992836 |
| N | 0.0005099360469167971 | 0.0006981331922861104 | 0.002520130256287969 |
| HT | 0.0009737098344693267 | 0.0015965841034678895 | 0.0011658615212810433 |
| N | 0.00046620046620046663 | 0.0007716049382716033 | 0.0 |(c)
(d)
### Chart: SPL16T2
| Category | A | B | D |
|---|---|---|---|
| HT | 0.001395982743802524 | 0.0021096299413603478 | 0.001243123465345688 |
| N | 0.0006597149077621336 | 0.0006121026726837433 | 0.0004252122453960226 |
| HT | 0.004351916347704582 | 0.0008582992729094393 | 0.0020530983268020352 |
| N | 9.968102073365225e-05 | 0.0001450957632037145 | 0.0 |
| HT | 0.0010367754251257204 | 0.0012865365076653893 | 0.0011758937892425915 |
| N | 0.0 | 0.0 | 0.0 |
| HT | 0.0006128060818024864 | 0.001401313731102522 | 0.001175844131846552 |
| N | 0.0007309676199583427 | 0.0013019684621279452 | 0.000789758261275203 |
| HT | 0.0005248889346530935 | 0.0013080599829450885 | 0.0006345850915353286 |
| N | 0.0007735577529941763 | 0.0004238748087547632 | 0.0006002160253571569 |
| HT | 0.0008217331957945513 | 0.0011453578181965722 | 0.0008510483875425886 |
| N | 0.0008872896780286479 | 0.001090162041039831 | 0.0010245050178156767 |
| HT | 0.0012828656176329006 | 0.0012856847062418704 | 0.00134222471425702 |
| N | 0.0003944650014607558 | 0.0008126473477554349 | 0.0007019350699400438 |
| HT | 0.0018728441657572682 | 0.000700025404664166 | 0.0018444567203918232 |
| N | 0.0008739546851916157 | 0.00041726233375473067 | 0.0004304778303917333 |
### Chart: GS3T11
| Category | A | B | D |
|---|---|---|---|
| HT | 0.005715446375950846 | 0.002243785606428632 | 0.005193627384145255 |
| N | 0.000549954170485792 | 0.0003043480633265458 | 0.0 |
| HT | 0.0035347606720549647 | 0.0005611197888438664 | 0.003548639987542997 |
| N | 0.0 | 0.0006183703889079133 | 0.0 |
| HT | 0.0019047488972109639 | 0.0012214670736710783 | 0.0004887585532746833 |
| N | 0.0005270092226613966 | 0.0010193679918450566 | 0.0 |
| HT | 0.0003836973704308268 | 0.0007395902434404881 | 0.000394736842105264 |
| N | 0.0009095878938736579 | 0.0006432723546816404 | 0.0003457216940363 |
| HT | 0.0005027149530224565 | 0.00057951826873634 | 0.00033068783068783 |
| N | 0.0008001201328528027 | 0.0008603097791815174 | 0.0 |
| HT | 0.0008733845694602295 | 0.0005521547713000061 | 0.00037436753694553915 |
| N | 0.00033940259070807787 | 0.00021070375052675955 | 8.769622029290524e-05 |
| HT | 0.0007236954600763123 | 0.0006387201301270641 | 0.0008768509130897084 |
| N | 0.0002365037854161481 | 0.0008082440897150942 | 0.0 |
| HT | 0.001282432573755967 | 0.0008360735776374414 | 0.000790564381586826 |
| N | 0.0 | 0.0010893246187363833 | 0.0 |
