# Supplementary material for: Expanding the range of editable targets in the wheat genome using the variants of the Cas12a and Cas9 nucleases
Source: Plant Biotechnol J. 2021 Jul 28;19(12):2428–41. doi: 10.1111/pbi.13669 (PMC8633491; doi:10.1111/pbi.13669)
Supplement: Supplementary file 18 — Legends S1 [file PBI-19-2428-s012.docx]

**Supplementary Figure Legends**

**Figure S1.** **The sequences of plasmids used in this study.** The two T-DNA borders are highlighted with purple color. The wheat U3 promoter is shown in purple letters, the wheat U3 terminator is underlined and highlighted with grey color. The two BsaI cut sites for annealed guide sequence oligoes subcloning are highlighted with dark green. The CDS of spectinomycin-resistance gene is shown in grey highlighted capital letters. The maize ubiquitin promoter is highlighted with yellow color. The CDS of maize codon optimized SpCas9 is shown as italic lowercase letters when the mutated nucleotides for getting Cas9-NG are shown in red capital letters. The 3xflag tag is highlighted with green color. The nucleus signal peptides coding sequences are shown in underlined capital letters. The NOS terminator is shown as italic capital letters. The Bar gene expression cassettes are highlighted with grey color. The enzyme cut site XmaI is shown in light blue, StuI is shown in brown.

**Figure S2. Gene editing efficiency comparison between the single target and multiple target CRISPR-LbCas12a constructs.** (a) Schematic illustration of the multiplex gene editing (MGE) CRISPR-LbCas12a constructs. Compared to the single target constructs, the MGE constructs have multiple tandemly arranged crRNAs driven by a single TaU6 promoter. The MGE construct targeting 4 regions is named as pA9LbCas12acr-4T (9LCCnv4T). The order of guide sequences is shown above the construct depiction. (b) The bar plots of gene editing efficiency for the CRISPR-LbCas12a constructs simultaneously targeting 1, 3 and 4 targets. The constructs are labeled as 1T, 3T and 4T, respectively. The gene editing efficiency was normalized by the protoplast transformation efficiency. The data are shown as mean ± standard error. Each construct has 3-5 biological replicates; the value of each replicate is shown as a dot. Student’s *t*-test was used to assess the significance of differences in gene editing efficiency between guides from the constructs with one, three or four crRNAs. * - *P* ≤ 0.05; ** - *P* ≤ 0.01; *** - *P* ≤ 0.001; NS - *P* > 0.05.

**Figure S3. The LbCas12a expression level in T1 progeny of (a) 9LCCnv8T and (b) 9LCCnvGS3T11 transgenic plants.** The quantitative RT-PCR of *Cas12a* was conducted with primers LB_25_F and LB_197_R (Table S12). The *TaActin* gene was used as reference. The expression level of LbCas12a was checked in only single replicate of each T1 plant. The plants are named with T0 transgenic line name followed by the tiller identity after the first dash, and then the progeny identity after the second dash.

**Figure S4.** **The gene editing efficiency in the MGE transgenic plants with and without high-temperature treatment.** The T1 progeny 9LCCnv8T transgenic plants after and before high-temperature treatment were genotyped by NGS. The gene editing efficiency of each target in the MGE construct was calculated. The data are shown as bar plots with mean ± standard error. The high-temperature treatment lasted for two weeks at 35 °C during the day (16 hours) and 30 °C during the night (8 hours). HT stands for high-temperature treatment; N stands for the normal condition. Each mean value was calculated using data from 3 to 21 biological replicates.

**Figure S5. The alignment of off-target sequences with 12 LbCpf1 targets selected based on Chinese Spring RefSeqv1.0.** The top three sequences for each target correspond to three homoeologous genomes. The PAM sequence TTTV is included on the 5’ end. The consensus nucleotides compared to the first designed target are shown as “.”. Only those sequences that contain the TTTV PAM are shown for targets PDST16, GW7T13, GW2T6 and An1T12.
